# Reprogramming the breast tumor immune microenvironment: cold-to-hot transition for enhanced immunotherapy

**DOI:** 10.1186/s13046-025-03394-8

**Published:** 2025-04-25

**Authors:** Saber Imani, Reyhaneh Farghadani, Ghazaal Roozitalab, Mazaher Maghsoudloo, Mahdieh Emadi, Atefeh Moradi, Behnaz Abedi, Parham Jabbarzadeh Kaboli

**Affiliations:** 1 Shulan International Medical College, Zhejiang Shuren University, Hangzhou, Zhejiang China; 2 Jeffrey Cheah School of Medicine and Health Sciences, Monash University Malaysia, Jalan Lagoon Selatan, Bandar Sunway, Subang Jaya, 47500 Selangor Darul Ehsan Malaysia; 3 Noncommunicable Diseases Research Center, Fasa University of Medical Sciences, Fasa, Iran; 4 Key Laboratory of Epigenetics and Oncology, The Research Center for Preclinical Medicine, Southwest Medical University, Luzhou, 646000 Sichuan China; 5 Department of Biology, Science and Research Branch, Islamic Azad University, Tehran, Iran; 6 Department of Life Sciences and System Biology, University of Turin, Turin, Italy; 7 Department of Basic Sciences, Faculty of Veterinary Medicine, University of Tabriz, Tabriz, Iran; 8 Department of Biochemistry, Faculty of Medicine, Medical University of Warsaw, Warsaw, 02-091 Poland

**Keywords:** Breast cancer, Hot tumor, Cold tumor, Immunotherapy, Cancer vaccine, Immune checkpoint

## Abstract

This review discusses reprogramming the breast tumor immune microenvironment from an immunosuppressive cold state to an immunologically active hot state. A complex interplay is revealed, in which the accumulation of metabolic byproducts—such as lactate, reactive oxygen species (ROS), and ammonia—is shown to impair T-cell function and promote tumor immune escape. It is demonstrated that the tumor microenvironment (TME) is dominated by immunosuppressive cytokines, including interleukin-10 (IL-10), transforming growth factorβ (TGFβ), and IL-35. Notably, IL-35 is produced by regulatory T cells and breast cancer cells. The conversion of conventional T cells into IL-35-producing induced regulatory T cells, along with the inhibition of pro-inflammatory cytokine secretion, contributes to the suppression of anti-tumor immunity. It is further demonstrated that key immune checkpoint molecules—such as PD-1, PDL1, CTLA-4, TIM-3, LAG-3, and TIGIT—are upregulated within the TME, leading to Tcell exhaustion and diminished immune responses. The blockade of these checkpoints is shown to restore T-cell functionality and is proposed as a strategy to convert cold tumors into hot ones with robust effector cell infiltration. The therapeutic potential of chimeric antigen receptor (CAR)T cell therapy is also explored, and targeting specific tumor-associated antigens, such as glycoproteins and receptor tyrosine kinases, is highlighted. It is suggested that CART cell efficacy can be enhanced by combining these cells with immune checkpoint inhibitors and other immunomodulatory agents, thereby overcoming the barriers imposed by the immunosuppressive TME. Moreover, the role of the microbiome in regulating estrogen metabolism and systemic inflammation is reviewed. Alterations in the gut microbiota are shown to affect the TME, and microbiome-based interventions are proposed as an additional means to facilitate the cold-to-hot transition. It is concluded that by targeting the metabolic and immunological pathways that underpin immune suppression—through combination strategies involving checkpoint blockade, CART cell therapies, and microbiome modulation—the conversion of the breast TME from cold to hot can be achieved. This reprogramming is anticipated to enhance immune cell infiltration and function, thereby improving the overall efficacy of immunotherapies and leading to better clinical outcomes for breast cancer patients.

**Supplementary Information:**

The online version contains supplementary material available at 10.1186/s13046-025-03394-8.

## Introduction

Breast cancer (BC) continues to be the most frequently diagnosed cancer among women, with over 2.3 million new cases estimated in 2020, according to Globocan. The incidence of BC is anticipated to rise, with more than 3 million new cases expected by 2040 [1]. This growing incidence highlights the urgent need for more effective treatment strategies [2].

In recent years, immunotherapy has emerged as a revolutionary approach in the battle against cancer, fundamentally changing treatment paradigms. This therapeutic strategy harnesses the body’s immune system to identify and eliminate cancer cells, gaining significant attention from the research community. Immunotherapy includes modalities such as adoptive cell transfer therapies, vaccines, cytokines, oncolytic viruses, small molecule drugs, and immune checkpoint inhibitors (ICIs) [3–6]. Among these, ICIs targeting programmed cell death protein 1 (PD-1), programmed death-ligand 1 (PD-L1), and cytotoxic T-lymphocyte-associated antigen 4 (CTLA-4) have been extensively studied in BC, showing promising results in specific subtypes [7, 8].

However, despite the initial success of ICIs in various solid tumors, their effectiveness in BC has been limited by tumor-intrinsic resistance mechanisms. This resistance reduces response rates and poses a significant challenge to the broader application of ICIs [5]. Contributing factors to this resistance include immunosuppressive cells within the tumor microenvironment (TME), the loss of immunogenic neoantigens, and the upregulation of additional immune checkpoints that hinder effective T-cell responses [5]. Specific subtypes of BC are characterized by limited T-cell infiltration and a low mutational burden, classifying them as immunologically cold tumors [9–11]. These cold tumors are less likely to elicit a robust immune response, making them less responsive to immunotherapy.

Recent studies have focused on investigating additional immune checkpoints within the TME to overcome the limitations of existing ICIs and improve the efficacy of immunotherapy for BC. New targets such as T-cell immunoglobulin and mucin domain 3 (TIM-3) [12], T-cell immunoglobulin (IgV) and immunoreceptor tyrosine-based inhibition motifs (ITIM) domain (TIGIT) [13], lymphocyte activation gene-3 (LAG-3) [14], and cluster of differentiation 47 (CD47) [15] are under investigation. New therapeutic approaches like cancer vaccines [16], oncolytic viruses [17], and bispecific antibodies (BsAbs) [18] are also being developed to enhance antitumor responses. These innovative immunotherapy options offer promising new treatment strategies, particularly for unresponsive tumors to traditional ICIs [19].

Transforming cold-to-hot tumors is an essential goal in BC immunotherapy. Hot tumors—characterized by robust immune cell infiltration and elevated expression of immunogenic markers—are more likely to respond to ICIs and other immunotherapeutic strategies [20, 21]. However, BC presents unique challenges, particularly in reversing the immunosuppressive microenvironment and addressing the generally low mutational burden contributing to immune resistance. For instance, cold tumors often lack neoantigens and pro-inflammatory signals that would otherwise recruit cytotoxic T-cells. Modifying these tumors to express higher levels of neoantigens or chemokines that attract immune cells may stimulate a more effective immune response and sensitize these tumors to immunotherapy [22].

This need for transforming cold tumors is especially relevant across the distinct subtypes of BC, each with its immune-related characteristics. Triple-negative BC (TNBC), known for its relatively high immunogenicity, still frequently demonstrates resistance due to factors like high levels of myeloid-derived suppressor cells (MDSCs) and regulatory T-cells (Tregs) that dampen immune activation [23]. On the other hand, hormone receptor-positive (HR^+^) or estrogen receptor-positive (ER^+^) BCs often exhibit minimal immune infiltration, requiring different approaches to induce immune activation, such as targeting tumor-associated macrophages (TAMs) or using cytokine-based therapies to recruit immune cells [24].

While immunotherapy, particularly ICIs, has revolutionized cancer treatment, its effectiveness in BC remains limited due to resistance mechanisms within the TME. Several key research gaps must be addressed to improve treatment outcomes [25]. One major challenge is overcoming immunosuppressive mechanisms within the TME, which restrict T-cell activation and infiltration, ultimately limiting the effectiveness of ICIs [26]. Tregs, MDSCs, and TAMs hinder immune responses, making it essential to develop strategies that either deplete these cells or reprogram them to promote antitumor immunity [27]. Potential approaches include targeting immunosuppressive factors with small molecules, using cytokines to enhance immune cell recruitment, and improving antigen presentation to boost T-cell activation.

Additionally, the potential of emerging immune checkpoints, such as TIM-3, TIGIT, LAG-3, and CD47, in BC remains unexplored, raising the need for further investigation into their roles and therapeutic applications. While ICIs targeting PD-1, PD-L1, and CTLA-4 have had limited success in BC, newer targets may provide alternative pathways to enhance immune responses [27, 28]. However, understanding how these novel checkpoints contribute to immune evasion in BC is crucial to determining their therapeutic potential. Future research should focus on how blocking these pathways can improve antitumor immunity and whether they can be effectively combined with existing immunotherapies to overcome resistance [29].

Another critical challenge is designing personalized immunotherapy strategies tailored to distinct BC subtypes, each presenting unique immune-related characteristics [30]. For example, TNBC has relatively high immunogenicity but still exhibits resistance due to abundant suppressive immune cells, necessitating combination therapies that enhance T-cell activity [25]. In contrast, hormone receptor-positive (HR^+^) BC typically shows minimal immune infiltration, requiring different approaches to stimulate immune responses, such as targeting tumor-associated macrophages or using cytokine-based therapies to attract immune cells to the tumor site. Addressing these gaps is essential for advancing BC immunotherapy, particularly in transforming immunologically cold-to-hot tumors that elicit stronger immune responses and improve patient outcomes [31].

The review delves into methods for transforming cold-to-hot tumors exhibiting a heightened immune reaction. By grasping how tumors evade the immune system and exploring new immunotherapy methods, BC’s receptiveness to treatment and clinical results can be enhanced. By understanding the specific immune barriers within each subtype, there is potential to develop tailored immunotherapy combinations—such as pairing ICIs with agents that disrupt immunosuppressive cells or enhance antigen presentation—that could convert cold tumors into hot, responsive ones.

### Tumor immune phenotypes and cold-to-hot tumor transition

Immunosuppressive factors in cold tumors, including Tregs, MDSCs, and anti-inflammatory cytokines, significantly hinder T-cell activation and function, promoting immune evasion. Conversely, hot tumors display dense immune cell infiltration, particularly cytotoxic CD8^+^ T-cells and activated macrophages, crucial in initiating effective anti-tumor responses. The increased expression of co-stimulatory molecules and pro-inflammatory cytokines in these tumors supports a more potent immune response against tumor cells [20, 32]. Interestingly, cold tumors provide a conducive environment for inducing ferroptosis, a form of cell death that may exploit the benefits of immunogenic cell death (ICD) in cancer cells while simultaneously counteracting immune suppression [32]. ICD kills cancer cells directly and stimulates an anti-tumor immune response. This process involves various regulatory mediators that drive cell death while reinforcing cancer immunosurveillance. Over time, ICD has been recognized in multiple cell death modalities—including pyroptosis, ferroptosis, cuproptosis, and PANoptosis—and can be harnessed therapeutically to address tumor antigen deficiencies, ultimately enhancing CAR-T therapy efficacy [33].

**Fig. 1:**
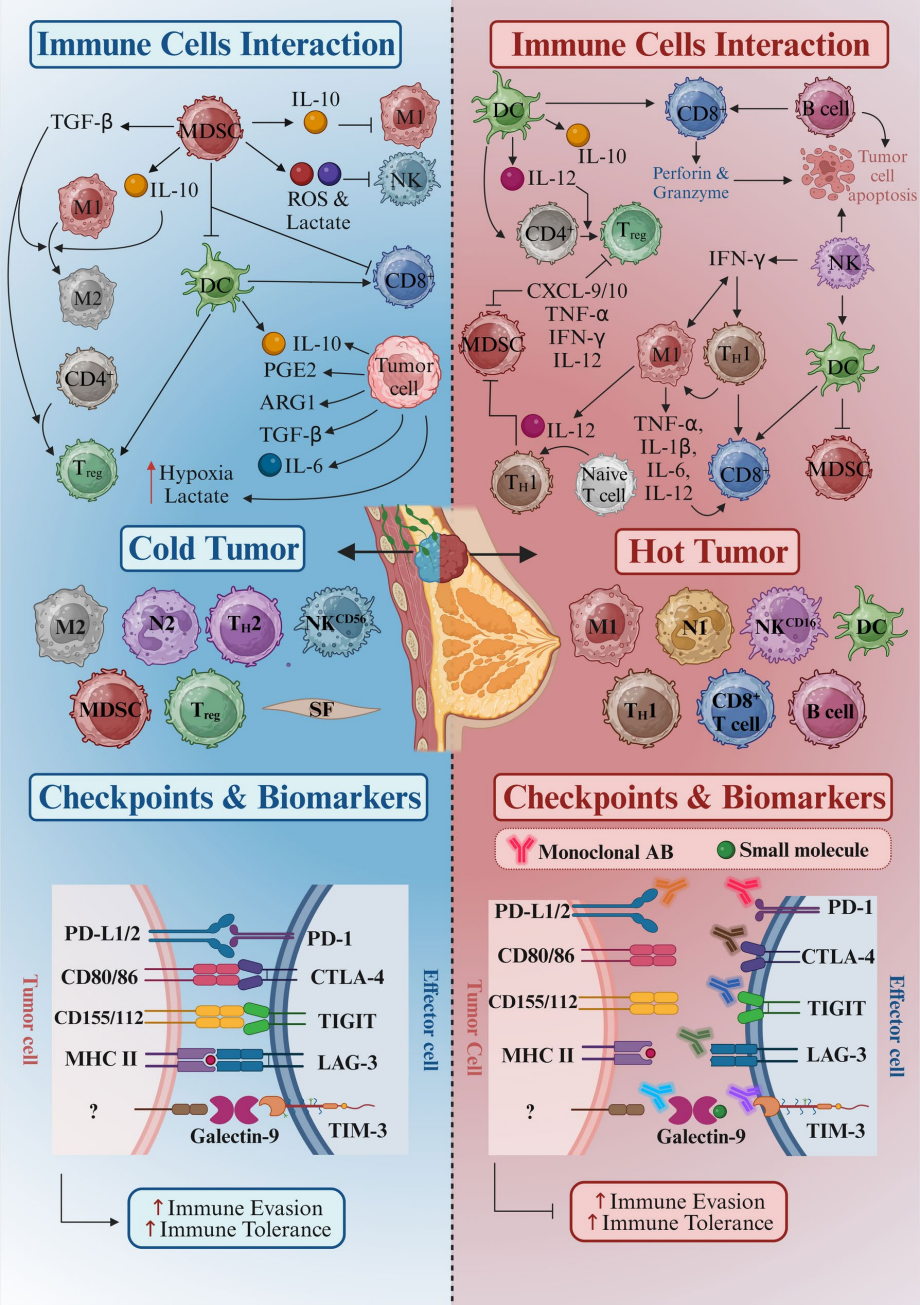
illustrates the critical transition of tumor immune phenotypes from cold to hot states in BC, emphasizing the intricate mechanisms of the cancer–immunity cycle that govern immune responses. This cycle commences with the release of tumor-associated antigens (TAAs) from dying cancer cells, which are subsequently taken up and processed by antigen-presenting cells (APCs), particularly dendritic cells (DCs). These APCs present the processed antigens with major histocompatibility complex (MHC) molecules to naive T-cells in secondary lymphoid organs. This leads to T-cell activation, clonal expansion, and the generation of effector T-cells

Figure 1. Tumor Immune Phenotypes and Cold-to-Hot Tumor Transition in BC Progression. This figure illustrates the transition of tumor immune phenotypes from cold to hot in BC progression, highlighting the differential immune profiles associated with these states. It features two main panels: the first panel depicts a schematic representation of cold tumors, characterized by low immune cell infiltration and the predominance of immunosuppressive cells (such as Tregs and MDSCs), illustrating their role in immune evasion. The second panel contrasts this with hot tumors, showcasing a rich immune landscape with high densities of effector T-cells, particularly CD8^+^ cytotoxic T-cells, and activated macrophages associated with improved anti-tumor responses. Arrows and annotations connect key immune cells and signaling pathways that facilitate this transition, emphasizing mechanisms such as cytokine production, immune checkpoint expression, and the influence of the TME on shaping immune responses. This comprehensive depiction will inform researchers about the dynamic immune landscape in BC, illustrating the potential for therapeutic interventions to enhance immune infiltration in cold tumors. *Created in BioRender. Jabbarzadeh Kaboli*,* P. (2024)*https://BioRender.com/m07h201.

On the other hand, hepatocyte growth factor (HGF), produced by cancer-associated fibroblasts (CAFs) and mesenchymal stromal cells (MSCs), triggers the mesenchymal-epithelial transition (c-MET) receptor tyrosine kinase (RTK) predominantly found on c-MET^+^ cancer cells and MDSCs. This activation of c-MET leads to increased secretion of transforming growth factor (TGF)-β, a cytokine critical for immune regulation. Higher levels of TGF-β cause M1 macrophages, typically pro-inflammatory and anti-tumor, to convert into M2 macrophages with immunosuppressive and pro-tumor traits. Furthermore, TGF-β helps transform CD4^+^ T helper cells into Tregs, known for their immune-suppressive properties. This immunosuppressive environment, influenced by M2 macrophages and Tregs, significantly weakens the body’s anti-cancer immune responses, enabling tumors to evade immune surveillance and destruction. The hypoxic TME is marked by high levels of reactive oxygen species (ROS), indoleamine 2,3-dioxygenase (IDO), arginase-1 (ARG-1), and TGF-β, along with an increased presence of MDSCs, M2 macrophages, Tregs, CAFs, and MSCs. These conditions create an environment that supports tumor immune evasion. This distinct immune landscape highlights the crucial role of c-MET expression levels in shaping the TME and affecting the immune system’s ability to detect and destroy cancer cells [34].

### Tumor-associated macrophages (TAMs)

Shifting the focus to another aspect of the TME, TAMs are identified as crucial. TAMs can be divided into two main phenotypes: M1 TAMs, which possess pro-inflammatory and anti-tumor properties, and M2 TAMs, which exhibit anti-inflammatory and pro-tumor characteristics. In this context, RNA sequencing has revealed a connection between macrophage-derived IL-10 and activating the c-MET/signal transducer and activator of transcription 3 (STAT3) signaling pathways. Pro-regenerative M2 macrophages are associated with markers like IL-10 and TGF-β, whereas pro-inflammatory M1 macrophages are linked with iNOS, TNF-α, IL-1, IL-12, IL-18, and IFN-γ markers [35]. Notably, HGF/c-MET signaling activates the phosphoinositide 3-kinase (PI3K)/protein kinase B (AKT) pathway while simultaneously inhibiting nuclear factor kappa-light-chain-enhancer of activated B cells (NF-κB) signaling in M1 macrophages, releasing IL-10 and TGF-β. Interestingly, treatment with HGF decreased the expression of iNOS, TNF-α, and IL-6 in these macrophages, indicating that c-MET signaling promotes the transition from M1 to M2 TAMs [36]. Conversely, inhibiting HGF/c-MET signaling with PHA-665,752 reversed these effects, maintaining the M1 macrophage phenotype by increasing levels of IL-1β and iNOS [37]. In BC, PD-1/PD-L1 signaling plays a critical role in suppressing T-cell activation, thereby hindering anti-tumor immunity. This pathway’s activation in the TME contributes to establishing a cold tumor characterized by low immune cell infiltration and poor response to immunotherapy [32]. Similarly, STAT3 signaling is frequently upregulated in both TNBC and HR^+^ BC, where it promotes tumor progression by driving the accumulation of immunosuppressive cell types such as Tregs and MDSCs, which further inhibit effective immune responses.

Additionally, STAT3 supports tumor cell survival and enhances the recruitment of other immune suppressive elements into the TME [38]. In contrast, the HGF/c-MET pathway is crucial in shaping the immune landscape, particularly in TNBC. HGF/c-MET signaling fosters the transition of TAMs from the M1 phenotype (anti-tumor, pro-inflammatory) to the M2 phenotype (pro-tumor, immunosuppressive). M2 TAMs create a pro-tumorigenic environment by secreting cytokines like IL-10 and TGF-β, inhibiting the activation of cytotoxic T-cells and promoting tumor growth and metastasis [39].

A balanced exploration of these pathways-PD-1/PD-L1, STAT3, and HGF/c-MET-reveals their interconnected roles in maintaining an immunosuppressive TME in BC. Effective therapeutic strategies could involve using colony-stimulating factor 1 receptor (CSF-1R) inhibitors to reprogram TAMs from the M2 phenotype to the M1 phenotype, thereby increasing pro-inflammatory cytokine production and antigen presentation. Additionally, PD-1/PD-L1 inhibitors can block immune checkpoint signaling, while STAT3 inhibitors can disrupt the immunosuppressive signaling pathways. By targeting these multiple pathways simultaneously, it becomes possible to convert cold tumors into hot tumors, characterized by increased immune cell infiltration and enhanced anti-tumor immune responses, thus improving the effectiveness of immunotherapy [40].

Besides, TAMs play a critical role in shaping the immune landscape of BC, frequently exhibiting a pro-tumorigenic M2 phenotype that fosters tumor growth and immune evasion [4, 42]. In TME, M2 TAMs secrete immunosuppressive factors such as IL-10 and vascular endothelial growth factor (VEGF), along with matrix metalloproteinases, which facilitate tumor progression and metastatic spread while simultaneously inhibiting the activation and proliferation of effector T-cells [43]. This accumulation of M2 TAMs is particularly pronounced in cold tumors, contributing to an immunosuppressive milieu that prevents effective cytotoxic T-cell recruitment and action, as illustrated in Fig. 1. Conversely, there is a growing interest in strategies to repolarize TAMs from the M2 phenotype to the M1 phenotype, characterized by the production of pro-inflammatory cytokines and enhanced antigen presentation capabilities [44]. This shift promotes a more inflammatory TME and boosts CD8^+^ T-cell responses against the tumor [42]. In TNBC, TAMs predominantly exhibit an M2 phenotype, contributing to an immunosuppressive TME and inhibiting cytotoxic T-cell activity, promoting tumor progression [45]. This is exacerbated by PD-1/PD-L1, STAT3, and HGF/c-MET signaling, which favors the transition from M1 to M2 macrophages.

In contrast, HR^+^ BC typically has a less immune-infiltrated TME, but M2 TAMs still play a role in suppressing immune responses by secreting IL-10 and TGF-β. However, in HR^+^ BC, estrogen signaling may also enhance M2 macrophage polarization, reinforcing the cold tumor state. Both subtypes share the potential to benefit from CSF-1R inhibitors and other strategies aimed at repolarizing TAMs to the M1 phenotype, which could shift the tumor from cold to hot, enhancing the effectiveness of immunotherapies [46, 47]. Emerging therapeutic approaches, such as CSF-1R inhibitors and immune-modulating agents, aim to reprogram TAMs to support anti-tumor immunity, facilitating the transition from a cold-to-hot tumor transition in BC [47, 48].

### Myeloid-derived suppressor cells (MDSCs)

MDSCs are a heterogeneous population of immune cells that include two main subsets: monocytic MDSCs (mMDSCs) and granulocytic MDSCs (gMDSCs) [49]. Both subsets contribute to the immunosuppressive landscape in BC progression and reinforce the cold tumor phenotype [49]. mMDSCs, which resemble classical monocytes, inhibit T-cell activation and proliferation by secreting ROS, ARG, and IDO within the TME. This depletion of crucial resources can induce T-cell apoptosis and prevent the activation of cytotoxic CD8^+^ T-cells, which is critical for effective anti-tumor immunity [50]. The presence of these gMDSCs creates an immunosuppressive microenvironment that hinders the activity of cytotoxic CD8^+^ T-cells. The accumulation of gMDSCs exacerbates the cold tumor phenotype and forms an immunological barrier that limits effective treatment responses (Fig. 1).

In HR^+^ BC, MDSCs contribute significantly to the immunosuppressive TME, limiting the effectiveness of immunotherapy [51, 52]. HR^+^ BC is typically characterized by low immune infiltration, but MDSCs accumulate in the TME, suppressing the function of immune cells such as CD8^+^ T-cells and DCs [26, 51]. These molecules deplete key resources like L-arginine, inducing oxidative stress and preventing T-cell activation. Additionally, MDSCs in HR^+^ BC can secrete cytokines, IL-6, and chemokine ligands (CCLs) like CCL2, suppressing T-cell function and recruiting more immune-suppressive cells to the TME, reinforcing the cold tumor phenotype. These mechanisms contribute to poor prognosis and reduced response to immunotherapy in HR^+^ BC. Targeting MDSCs, either by inhibiting their recruitment to the tumor site, promoting their differentiation into mature immune cells, or combining MDSC-targeting strategies with ICIs, holds potential for improving immune responses and enhancing the efficacy of immunotherapy in HR^+^ BC [53].

Elevated levels of gMDSCs in TNBC have been associated with poor prognosis and reduced responsiveness to immunotherapy [54, 55]. Many in vitro and in vivo studies are focused on therapeutic strategies to target MDSCs and promote this transition [56]. Approaches include inhibiting the recruitment of MDSCs to the tumor site and promoting their differentiation into mature myeloid cells that exhibit anti-tumor functions. Additionally, combining MDSC-targeting therapies with ICIs aims to enhance CD8^+^ T-cell responses by reversing the immunosuppressive effects of MDSCs. These strategies are crucial for transforming BC’s immunological landscape and improving immunotherapy’s efficacy.

### Regulatory T cells (Tregs)

Tregs are pivotal in suppressing various immune cells, including CD8^+^ and CD4^+^ T-cells, DCs, and natural killer (NK) cells. Targeting key markers such as CD25, forkhead box p3 (FoxP3), TGF-β receptor, IDO-1, ARG-1, and glutaminase (GLS) shows promise inducing antitumor immunity [57]. In colorectal cancer, liver metastasis, CD4^+^FOXP3^+^ Tregs, and increased levels of α-smooth muscle actin, HGF, and c-MET indicate potential therapeutic targets for this metastatic form of colorectal cancer. Moreover, in gastric cancer, HGF and c-MET have been implicated in the accumulation of Tregs in peripheral blood. Notably, treatment with an anti-HGF antibody reduced the number of circulating Tregs expressing c-MET in gastric cancer patients, underscoring HGF’s role in promoting Treg accumulation indirectly through c-MET-expressing monocytes. These findings suggest that therapies targeting HGF/c-MET, potentially in combination with immune checkpoint inhibitors, could benefit cancer treatment.

In TNBC, the TME is often characterized by a high level of immune cell infiltration, yet immune suppression remains a significant challenge. Tregs, particularly those expressing FoxP3, are frequently enriched in the TME of TNBC and are crucial players in the immunosuppressive landscape. These Tregs suppress the activity of effector T cells, such as CD8^+^ cytotoxic T-lymphocytes (CTLs) and CD4^+^ helper T cells, reducing their ability to mount effective immune responses against tumor cells [58]. The presence of Tregs in TNBC is often associated with poor prognosis, as their suppression of immune responses can significantly limit the efficacy of ICIs. Molecularly, Tregs in the TME of TNBC secrete immunosuppressive cytokines such as TGF-β and IL-10, which further enhance the immunosuppressive environment and promote tumor growth. Additionally, Tregs can induce metabolic changes that further contribute to immune evasion, such as upregulating adenosine production via CD73 to inhibit T-cell function. Targeting Tregs in TNBC using strategies such as CD25 or FoxP3 inhibitors or blocking key signaling molecules like TGF-β has shown promise in preclinical studies by disrupting these immunosuppressive pathways and boosting the effectiveness of ICIs [45, 59].

In HR^+^ BC, the TME typically displays low levels of immune infiltration compared to TNBC. However, Tregs still play a significant role in maintaining an immunosuppressive TME that inhibits the function of CTLs and other immune effectors. HR^+^ tumors tend to have a higher proportion of TAMs and MDSCs, which work synergistically with Tregs to create a microenvironment that favors tumor immune escape. Tregs in HR^+^ BC have been shown to suppress the activation of CD8^+^ T cells and promote the accumulation of immunosuppressive cytokines, such as TGF-β and IL-10, within the TME [60]. Additionally, HR^+^ tumors often rely on estrogen and progesterone signaling to promote tumor growth, which can also influence Treg recruitment and function. Estrogen has been shown to enhance Treg expansion and suppress CTL function in HR^+^ BC, making it a key player in immune modulation in this subtype. Molecularly, strategies targeting TGF-β, which plays a central role in Treg induction and function, and GLS and other metabolic pathways, may effectively reverse the immunosuppressive effects of Tregs in HR^+^ BC. By modulating the TME to reduce Treg accumulation or enhance immune cell recruitment, HR^+^ tumors can become more responsive to immunotherapy, including ICIs and combination therapies [61].

In addition, Tregs play a pivotal role in the TME of BC, with distinct subsets influencing immune responses and cold-to-hot tumor transition [62]. When Tregs are depleted or their function inhibited, CD8^+^ T-cell activity can be restored, creating a more inflammatory microenvironment that facilitates the recruitment of pro-inflammatory helper T-cells (Th1) [63, 64]. Targeting these Treg subsets represents a promising therapeutic approach in BC. Strategies aimed at reducing naturally occurring Tregs (nTreg) populations or blocking their suppressive functions could enhance the effectiveness of immunotherapies. nTregs typically accumulate in cold tumors and suppress the activation of CTLs by releasing immunosuppressive cytokines like IL-10 and TGF-β. This action dampens the immune response, reinforcing the cold tumor phenotype [65].

Conversely, induced Tregs (iTregs) differentiate from naive T-cells in response to TAAs within the TME. Although they share immunosuppressive functions with nTregs, iTregs exhibit plasticity, allowing them to adapt to the local cytokine environment. Under pro-inflammatory conditions, iTregs can adopt a phenotype that supports anti-tumor activity [66].

### Tumor-associated neutrophils (TANs)

Tumor-associated neutrophils (TANs) are pivotal immune cells that populate the tumor microenvironment (TME) and can be drawn in by IL-8 through CXCR1/CXCR2 signaling. They can suppress CD8^+^ T-cell function via TNF-α–mediated nitric oxide production. Similar to TAMs, TANs can shift between an anti-tumoral (N1) and a pro-tumoral (N2) phenotype, and Type I interferons (IFN-α and IFN-β) can promote their differentiation toward the N1 subset, reducing the pro-tumor impact of neutrophil extracellular traps and TNF-α expression [67]. Clinically, elevated TAN levels or a high neutrophil-to-lymphocyte ratio are frequently correlated with a poor prognosis, highlighting the significant influence of neutrophils on tumor progression. TGF-β has emerged as a pivotal regulator of neutrophil plasticity, orchestrating the transition from the antitumor N1 state to the immunosuppressive N2 phenotype [68]. This cytokine-driven polarization is underpinned by complex signal transduction pathways involving SMAD proteins and other transcription factors, ultimately reshaping neutrophil functional outputs and reinforcing an immunosuppressive TME [69]. In parallel, IL-10 reduces the production of pro-inflammatory cytokines such as IL-1β and TNF-α in neutrophils, further stabilizing the N2 phenotype and dampening anti-tumor immune responses [70].

In TNBC, TANs predominantly adopt the N2 phenotype, contributing to an immune-evasive milieu. TNBC frequently presents with extensive immune cell infiltration. Yet, its response to immunotherapy remains limited, partly because N2 TANs secrete immunosuppressive mediators like IL-10, TGF-β, and ROS, which curtail T-cell functions [71]. The CXCL8/CXCR1/2 axis has been identified as a key signaling pathway driving neutrophil recruitment to the tumor site; once recruited, TANs are exposed to local cytokines, including TGF-β, that further reinforce their immunosuppressive characteristics [59, 71]. These observations offer a rationale for targeting TGF-β receptors or CXCR2 signaling—approaches shown in preclinical studies to reduce TAN density and bolster the effectiveness of ICIs [72].

Additionally, c-MET signaling holds considerable relevance for TAN biology in TNBC. Activated by its ligand HGF, c-MET fosters TAN recruitment and activation, often amplifying downstream pathways linked to proliferation and invasive potential. Inhibiting c-MET diminishes neutrophil infiltration and retards tumor progression, demonstrating that c-MET inhibitors can potentially counteract neutrophil-mediated suppression of T cells, thereby synergizing with ICIs [34]. Beyond c-MET, other crucial targets include PD-1/PD-L1 and STAT3, integral to dampening immune surveillance. PD-1/PD-L1 blockade restores T-cell functionality, whereas STAT3 inhibition diminishes the accumulation of various immunosuppressive cells (e.g., Tregs, MDSCs, TANs) [32, 73]. The coordinated disruption of multiple signaling axes could thus reprogram the TME from cold to hot.

Although HR^+^ BC exhibits generally lower immune cell infiltration, TANs remain impactful. Like TNBC, TANs in HR^+^ BC adopt the N2 phenotype, imposing immunosuppression by releasing TGF-β, IL-10, and other inhibitory factors [74]. TGF-β secretion by tumor cells and immune components is particularly abundant in HR^+^ BC. It promotes not only N2 TAN polarization but also the expansion of MDSCs and Tregs. These cell populations further dampen effector T-cell responses and sustain immune evasion. Additionally, IL-6 significantly influences TAN function in HR^+^ BC by driving neutrophils toward the N2 state.

Meanwhile, CCL2 chemokine production attracts monocytes and neutrophils to collaborate in tumor-supportive processes, including vascular remodeling, metastasis, and immune suppression [75]. High IL-6 and CCL2 levels frequently correlate with worse clinical outcomes, reflecting a synergy among TANs, tumor cells, and other suppressive immune populations that ultimately restrict T-cell-mediated clearance. Of particular interest in HR^+^ BC is the role of estrogen signaling: emerging data suggest estrogen can boost the expression of CXCL8 and CCL2, which in turn enhances neutrophil trafficking into the TME, thus reinforcing the N2 phenotype [76]. Such mechanisms may underlie why certain HR^+^ BC tumors display therapy resistance, particularly when faced with immunotherapeutic strategies that do not address TAN-mediated suppression.

Therefore, the interplay between TANs and the local TME varies by breast cancer subtype. In TNBC, TGF-β, CXCR2, and c-MET/HGF axes dominate TAN recruitment and immunosuppressive polarization, whereas in HR^+^ BC, TGF-β, IL-6, CCL2, and estrogen signaling help shape a similarly suppressive TME [61]. Despite these differences, both subtypes share the pathophysiologic hallmark of TAN-driven immune evasion. Consequently, therapies aimed at reprogramming TANs from an N2 to an N1 phenotype have garnered considerable attention. Clinically, these interventions focus on (i) limiting TAN infiltration (e.g., CXCR2 blockade), (ii) inhibiting critical signaling mediators such as TGF-β and c-MET, and (iii) combining these strategies with ICIs (e.g., PD-1/PD-L1 inhibitors) or STAT3 inhibitors to alleviate the broader immune suppression within the TME. Such combination regimens can potentially enhance effector T-cell activity, reverse immunosuppression, and ultimately transform cold tumors into hot, inflamed ones more susceptible to immunotherapy. These strategies may substantially improve response rates and clinical outcomes in TNBC and HR^+^ BC by mitigating TAN-mediated immunosuppression.

### Natural killer (NK) cells

NK cells constitute a critical component of the innate immune system, capable of recognizing and eliminating tumor cells independently of antigen presentation. Their role in BC has garnered increasing attention as a potential therapeutic target, not only for their direct cytotoxicity but also for their interplay with other immune cells [67, 77]. Although NK cells are typically associated with “hot” tumors—that is, those with abundant immune cell infiltration—their function in “cold” tumors, including many types of BC, remains less well elucidated. In these cold tumors, the tumor immune microenvironment (TIME) often suppresses NK cell effector functions. For instance, upregulation of inhibitory receptors such as PD-1 and TIGIT and tumor-driven immune evasion strategies can stifle NK cell cytotoxicity [77].

The importance of NK cells in reshaping BC from cold to hot tumors is increasingly apparent. Targeting immune checkpoints, such as PD-1, modulates the IFN-γ–CXCL9/10–CXCR3 axis, a signaling pathway imperative for infiltrating and activating numerous immune cells within the breast TME [78]. CXCL9, a crucial chemokine in this network, facilitates the influx of CTLs, M1 macrophages, and CD56^dim^ NK cells while exhibiting an inverse relationship with immunosuppressive M2 macrophages [79]. This highlights CXCL9’s central role in promoting a more pro-inflammatory and immune-permissive BC microenvironment. Recent studies have shown that BC enriched with NK cells demonstrates concomitant upregulation of CCL5/IFNG–CXCL9/10 transcripts. IFN-γ–producing CD16^+^ NK cells can, in turn, stimulate BC cells to secrete CXCL9/10, driving additional immune cell infiltration. These processes facilitate tumor growth control and induce the differentiation of CD16^+^ NK cells into tissue-resident CD16^−^ CD103^+^ NK cells, prolonging immune surveillance and BC suppression [80].

In line with these findings, serum CCL5/CXCL9 levels appear to be promising predictive biomarkers for identifying NK cell–rich breast tumors that exhibit better clinical responses to anti-HER2 therapies, underlining the therapeutic value of harnessing NK cell-mediated immune modulation. Recently, a next-generation immunocytokine, LH05—a tumor-conditional anti-PD-L1/IL-15 prodrug—has been developed to minimize systemic toxicity by masking IL-15 until it is enzymatically activated within the TME [81]. The subsequent release of an IL-15 superagonist enhances CD8^+^ T-cell and NK cell infiltration by stimulating CXCL9/10 production, thereby converting cold tumors into hot ones. Combining LH05 with other modalities, including oncolytic viruses or checkpoint inhibitors, amplifies antitumor effects in advanced and metastatic models while mitigating toxicities [81].

Additional complexity arises from different NK cell subsets within BC. NK1 cells, which circulate in the blood, possess robust cytotoxic capabilities characterized by high expression levels of CD16, CD161, CD38, granzyme B (GZMB), perforin (PRF1), and NKG7, and are thus closely associated with antitumor immunity [82]. Conversely, NK2 cells reside preferentially in tissues and exhibit reduced cytotoxic potential, expressing CD56, CD27, CD44, CD314 (NKG2D), and CD335 (NKp46), but lower levels of GZMB and perforin. They appear more involved in tissue remodeling and immune regulation, reflecting a less potent direct antitumor function [82]. An elevated NK2 profile within the TME may correlate with a cold tumor phenotype and poor prognosis, emphasizing how the NK1–NK2 balance could influence therapeutic responses.

Subtype-specific factors also modulate NK cell function in BC. In TNBC, an intensely inflammatory TME paradoxically coexists with heightened immunosuppression, often driven by cytokines such as TGF-β that blunt NK cell activity [83]. Moreover, TNBC frequently engages CXCR2 and c-MET signaling pathways for NK cell trafficking and activation [84], suggesting that targeting these pathways—alongside PD-1/PD-L1 and STAT3 blockade—may be especially valuable for reinvigorating NK function in TNBC [85]. HR^+^ BC, in contrast, generally exhibits lower immune infiltration but still experiences NK cell suppression via TGF-β, IL-6, CCL2, and estrogen signaling, each of which reduces NK cell cytotoxicity and infiltration [86, 87]. Disrupting these immunosuppressive loops is critical to unleashing NK cell-mediated tumor clearance in HR^+^ BC.

Immunotherapy strategies that harness NK cells can be synergistic with existing checkpoint inhibitors [88]. NK cells express PD-1; in specific BC contexts, tumor overexpression of PD-L1 fosters NK cell exhaustion. Blocking this axis can restore NK cell cytotoxicity, while concurrent STAT3 inhibition can lessen Tregs and MDSCs’ expansion, undermining NK cell effector functions [89, 90]. By integrating NK cell–focused therapies with established immune checkpoint blockade and other targeted interventions, it may be possible to overcome immune resistance, enhance tumor immunogenicity, and tailor precision immunotherapy approaches for TNBC and HR^+^ BC.

Taken together, these insights underscore the vital contribution of NK cells to the immunobiology of BC and underscore their therapeutic promise. A robust NK1 subset strongly correlates with an active antitumor response and improved immunotherapy outcomes, whereas an expanded NK2 population may favor immune evasion. Understanding and manipulating the NK1–NK2 balance, inhibiting immunosuppressive signaling circuits (e.g., TGF-β, CCL2, CXCR2, c-MET), and combining NK cell–activating strategies with checkpoint inhibitors represent key opportunities to convert cold BC tumors into hot, immune-infiltrated lesions. Strategies like adoptive NK cell transfer, engineered NK cells, and immunocytokine approaches further broaden the potential for NK cell-based immunotherapy. By focusing on these mechanisms, clinicians and researchers can refine therapeutic paradigms and improve clinical outcomes across diverse BC subtypes.

### Immune profile dynamics in the cold-to-hot transition of BC

Transforming immunologically cold-to-hot tumors is a critical strategy for enhancing the therapeutic potential of BC treatments, as hot tumors are inherently more responsive to immunotherapy. Achieving this transformation requires a deep understanding of the TME and immune cell dynamics specific to each BC subtype, which allows for precise therapeutic interventions to activate the immune system in cold tumors. This review highlights the importance of such detailed analysis to uncover the subtype-specific immune dynamics essential for effectively shifting cold tumors to an immunologically active state. This comprehensive analysis of immune dynamics across BC subtypes provides a foundational framework for advancing immunotherapy strategies. By delineating the differences between cold and hot tumors and identifying key immune markers and pathways, targeted approaches can be devised to reprogram the TME [91]. Understanding the specific immune challenges within each BC subtype and the role of biomarkers in patient stratification is crucial for developing precise, personalized therapies that can enhance the therapeutic response and improve patient outcomes. The insights from this analysis offer a roadmap for future research and clinical applications, highlighting the transformative potential of cold-to-hot transition strategies in BC therapy [92].

Our analysis, based on RNA-Seq data from The Cancer Genome Atlas (TCGA) [93], examined three BC subtypes: BRCA, TNBC, and HER2^+^ BC tumors (Fig. 1), along with Lum A and Lum B subtypes (Figure S1). Tumors were classified as cold or hot based on immune infiltration scores and overall gene expression profiles. Using the immune cell deconvolution tool quanTIseq [94], we identified and characterized the immune cell populations and their functional states within these subtypes, providing valuable insights into their immune landscapes [23, 95]. We found significant differences in the expression profiles of immune-related genes between cold and hot tumors. Hot tumors showed much higher levels of immune activation. Functional groups like Immune checkpoints, Immune Modulators, Immune effectors, and TME-related genes were significantly upregulated in hot tumors, as heatmaps and boxplots show their more active immune engagement.

**Fig. 2:**
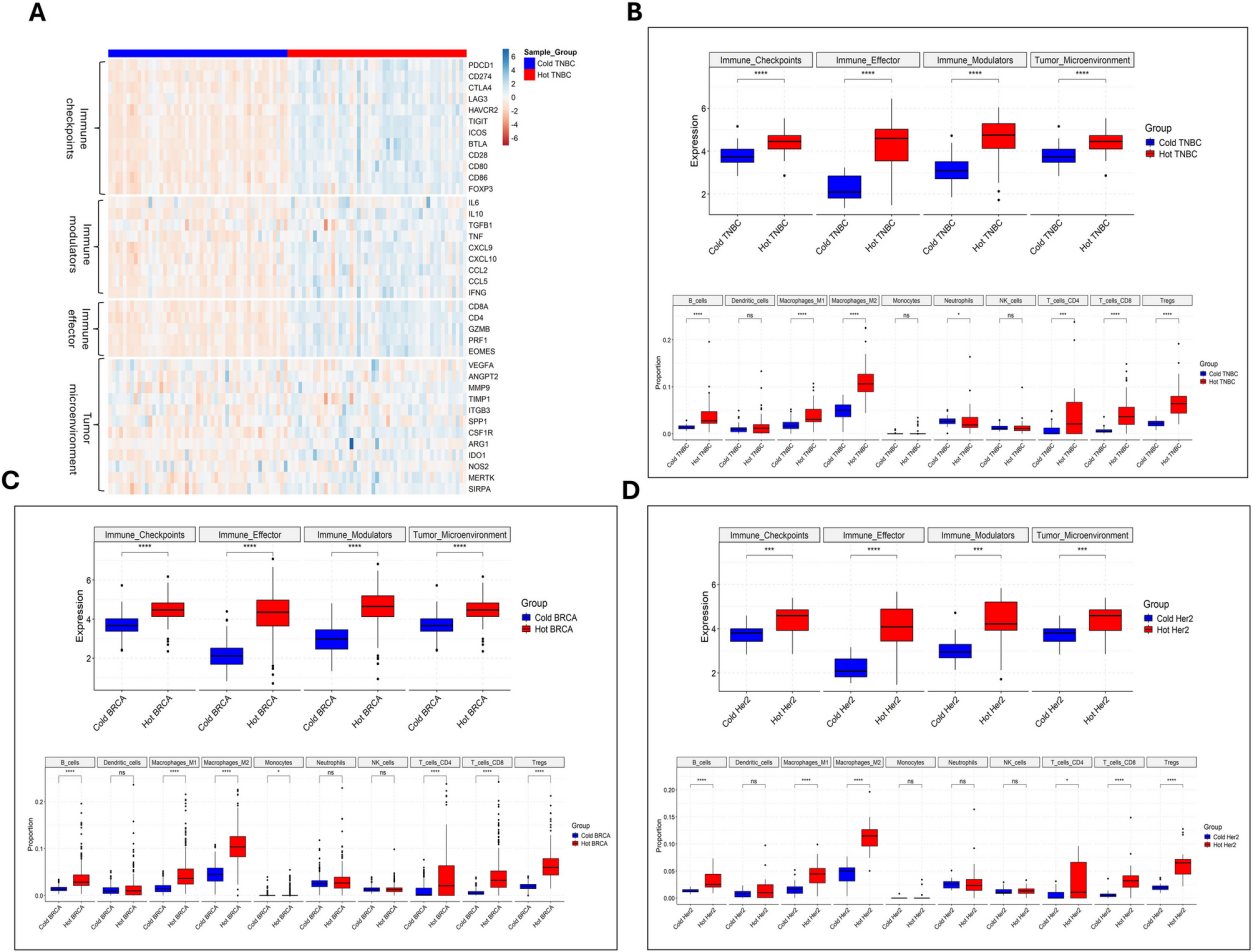
Immune Profiling of BC: Cold vs. Hot Tumors. This figure compares immune profiling in cold versus hot breast tumors based on The Cancer Genome Atlas (TCGA) data analysis. It comprises two panels: Panel **A** presents a bar graph comparing levels of various immune cell types (e.g., CD8^+^ T-cells, Tregs, and macrophages) infiltrating cold and hot BC tumors across different subtypes (luminal A, HER2^+^, and triple-negative), revealing significant differences in immune cell presence—hot tumors exhibit a higher density of effector T-cells, while cold tumors show a predominance of immunosuppressive cells. Panel **B** features a heatmap that displays the expression levels of key immune-related genes (such as PD-1, CTLA-4, and TIM-3) across various BC stages and subtypes. This visually distinguishes between hot and cold tumors, correlating gene expression profiles with clinical parameters like tumor stage, subtype, and patient outcomes, including survival rates. Annotations emphasize the significant correlations found, indicating how specific immune gene expressions are associated with favorable outcomes in hot tumors versus poor prognoses in cold tumors. This figure underscores the importance of immune profiling in BC, highlighting its implications for immunotherapeutic strategies to enhance the immune response

In TNBC, a particularly aggressive and challenging subtype, hot tumors showed intense infiltration by CTLs, M1 macrophages, and helper T-cells, key components of an effective anti-tumor immune response (Fig. 2A). In contrast, cold TNBC tumors lacked these critical immune cells. Interestingly, dendritic cells, NK cells, and monocytes showed minimal differences between cold and hot tumors, suggesting these cells could be targeted to boost immune activation further (Fig. 2B). Similarly, in BRCA tumors overall, hot tumors had higher effector cell activity. However, dendritic cells, NK cells, and neutrophils did not change much, indicating persistent immune bottlenecks that could be addressed to enhance immune response (Fig. 2C). This presents a promising therapeutic opportunity to develop strategies that increase dendritic cell recruitment or boost NK cell activity to drive immune engagement in TNBC. Additionally, biomarkers such as PD-L1 expression and tumor mutational burden (TMB) may be necessary in predicting TNBC patients’ response to ICIs. High TMB and PD-L1 positivity could help guide ICIs, offering an effective patient stratification tool for personalized therapy.

In BRCA tumors, hot tumors were characterized by higher effector cell activity, including greater CTLs and M1 macrophage infiltration. However, dendritic cells, NK cells, and neutrophils showed slight variation between cold and hot BRCA tumors, indicating persistent immune bottlenecks. Targeting these immune populations, particularly by enhancing dendritic cell activation or modulating NK cell activity, could potentially overcome these bottlenecks, leading to a more robust immune response [96]. Furthermore, PD-L1 expression could serve as a predictive biomarker for BRCA tumors, helping identify patients who might benefit from ICI therapy. In cases where PD-L1 expression is low, alternative strategies, such as combining ICIs with agents that activate NK cells or dendritic cells, may enhance therapeutic outcomes [97].

HER2^+^ tumors, despite their distinct molecular characteristics, had immune profiles similar to TNBC and BRCA. Hot HER2 tumors exhibited elevated immune activation with higher expression of immune-related genes and greater infiltration of effector cells (Fig. 2D). Yet, like other subtypes, dendritic cells, neutrophils, monocytes, and NK cells remained relatively unchanged, suggesting these populations might limit full immune mobilization. These findings highlight the importance of understanding the immune differences within BC subtypes to develop more effective therapies. TMB and HER2 expression levels could be critical biomarkers for predicting response to HER2-targeted therapies and ICIs in HER2^+^ BC patients. Higher TMB may correlate with better response rates to ICIs, while HER2 overexpression could guide HER2-targeted therapies in conjunction with immune checkpoint blockade [98].

Luminal A and B tumors, typically HR^+^, present a distinct immunological landscape [99]. Hot Luminal tumors exhibited higher immune activation with a marked increase in the infiltration of T-cells and macrophages. However, as in other subtypes, DCs and NK cells showed relatively unchanged levels, highlighting another potential bottleneck. Targeting these populations to enhance dendritic cell function or NK cell activation is a viable therapeutic strategy. Additionally, hormone receptor status and PD-L1 expression should be closely monitored in these tumors to optimize the use of ICIs. Luminal A tumors, with generally lower PD-L1 expression, might require combination therapies to enhance immune activation. In contrast, Luminal B tumors, which tend to have higher immune activity, could benefit from more direct immune modulation strategies [100].

The variations between cold and hot tumors offer insights for designing treatments that transition cold tumors to hot. Therapies can become more precise and effective by targeting immune bottlenecks, such as enhancing dendritic cell activation or modulating NK cell activity. For instance, TNBC could benefit from strategies that increase dendritic cell recruitment and the activation of NK cells. At the same time, HER2^+^ subtypes might see better outcomes by boosting effector cell activity and addressing neutrophil and monocyte dynamics. This comprehensive analysis of immune dynamics across BC subtypes provides a foundational framework for advancing immunotherapy strategies. By delineating the differences between cold and hot tumors and identifying key immune markers and pathways, targeted approaches can be devised to reprogram the TME, ultimately improving therapeutic responses and patient outcomes. The insights from this analysis offer a roadmap for future research and clinical applications, highlighting the transformative potential of cold-to-hot transition strategies in BC therapy.

Beyond the differences in immune cell infiltration, biomarker-based patient stratification is crucial for optimizing immunotherapy in BC. PD-L1 expression, a well-established predictor of response to ICIs, was significantly higher in hot tumors across TNBC, HER2^+^ BC, and Luminal BC subtypes, reinforcing its role as a key biomarker (Fig. 1). TMB, a surrogate for neoantigen load, was highest in TNBC and HER2^+^ BC tumors, supporting their potential sensitivity to immune-based therapies. Immune gene signatures, including interferon-gamma signaling and T-cell inflamed gene expression profiles, correlated strongly with immune infiltration in hot tumors, providing additional criteria for stratifying patients who may benefit from immunotherapy. Integrating these biomarkers with immune profiling offers a more refined approach to patient selection and therapeutic decision-making [40].

Despite hot tumors in each BC subtype, persistent immune suppression and resistance mechanisms necessitate novel therapeutic strategies to enhance immune activation. The observed lack of significant dendritic cell, NK cell, and monocyte differences between cold and hot tumors across subtypes suggests that these populations could be targeted to improve immune responses. Enhancing DCs priming may be particularly beneficial in TNBC and HER2^+^ tumors, where antigen presentation is critical for robust T-cell activation. Similarly, NK cell modulation could improve immune-mediated tumor clearance, given their relatively unchanged presence in hot tumors [101]. Combining ICIs with immune-modulating agents such as TGF-β inhibitors or STING agonists could help overcome residual immune suppression and facilitate cold-to-hot tumor transitions. Integrating high-dimensional immune profiling with targeted immunotherapy approaches provides a framework for refining BC treatment strategies and improving patient outcomes. By delineating the immune architecture of BC subtypes and identifying actionable immunological bottlenecks, this analysis establishes a roadmap for advancing precision immunotherapy and unlocking the full potential of immune-based BC therapies [32, 61].

### Immune checkpoint Inhibition in the cold-to-hot transition of BC

**Fig. 3:**
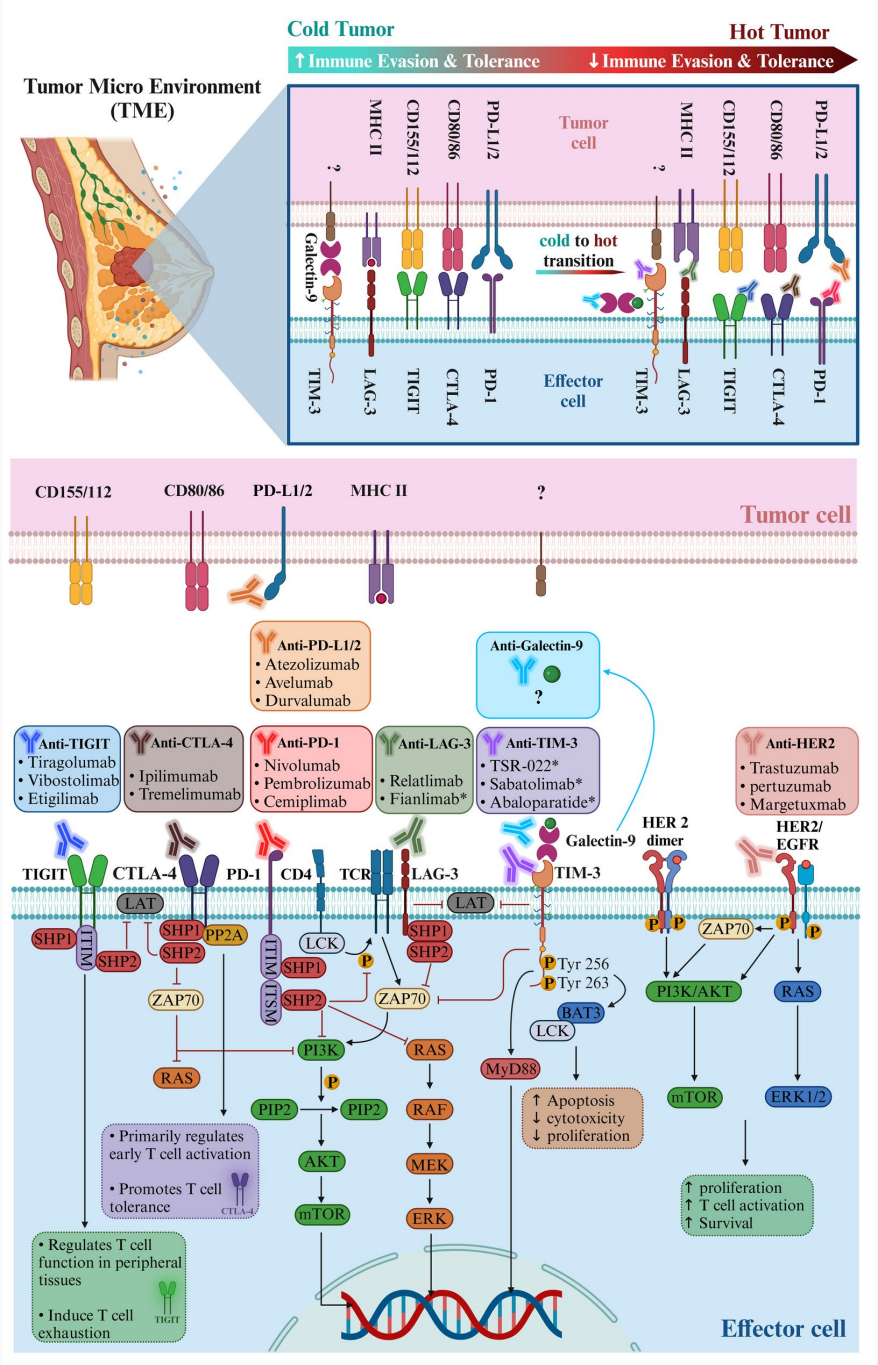
Mechanisms of ICIs in Tumor Transformation. This figure illustrates the critical mechanisms by which ICIs (such as inhibitors of TIM-3, PD-1, CTLA-4, LAG-3, and TIGIT) facilitate the cold-to-hot transition in BC. It presents a central diagram detailing the structure and function of these checkpoint proteins, showing TIM-3’s role in inhibiting T-cell activation and promoting Treg function. The PD-1/PD-L1 pathway is highlighted to illustrate how PD-1 engagement with PD-L1 on tumor cells inhibits T-cell responses, creating an immunosuppressive environment. CTLA-4 is depicted as a negative regulator of T-cell activation through its competitive inhibition of CD28. LAG-3 and TIGIT are included to show their contribution to dampening T-cell responses and supporting immune tolerance. Arrows depict the signaling pathways activated by these checkpoints. At the same time, annotations emphasize the therapeutic targeting of these pathways using monoclonal antibodies or small molecules to reverse immune suppression, thereby enhancing T-cell responses against tumors and promoting the cold-to-hot transition. This comprehensive representation informs the design of strategies to improve immunotherapy’s effectiveness in BC. *Created in BioRender. Jabbarzadeh Kaboli*,* P. (2024)*https://BioRender.com/w83p028

ICIs have emerged as a promising first-line strategy to convert cold tumors into hot tumors in BC therapy. The main ICIs studied in both monotherapy and combination therapy in BC primarily target PD-1, PD-L1, CTLA-4, TIM-3, LAG-3, and TIGIT molecules that typically suppress immune responses [19, 102–104]. These checkpoints regulate immune suppression during the cold-to-hot transition in tumors (Fig. 3). For instance, TIM-3 impedes T-cell activation and promotes the function of Tregs, while the PD-1/PD-L1 axis contributes to an immunosuppressive TME. CTLA-4 inhibits T-cell activation by competing with CD28, and both LAG-3 and TIGIT help maintain immune tolerance. By targeting these pathways, ICIs can reverse immune suppression, boost T-cell responses against the tumor, and shift the cancer from cold to hot, thus improving the efficacy of immunotherapy. In this section, we explored the molecular mechanisms and therapeutic strategies behind using ICIs in BC. We discussed how these therapies facilitate the cold-to-hot transition, improving the clinical outcomes of cancer immunotherapy.

### T-cell Immunoglobulin and mucin-domain containing protein 3 (TIM-3)

#### Structure of TIM-3

TIM-3, CD366, is a type I transmembrane protein in the IgV superfamily. It comprises 302 amino acids in humans and is encoded by the hepatitis A virus cellular receptor 2 gene on chromosome 5q33.2. This gene is part of the TIM family, which includes TIM-1, TIM-3, and TIM-4 [105, 106]. TIM-3 suppresses the response of CD4^+^ and CD8^+^ T-cells by inducing cell death or exhaustion. At the same time, it enhances the ability of macrophages to clear pathogens by binding to galectin-9 (Gal-9) [107].

The structure of TIM-3 consists of several parts: a membrane-distal N-terminal variable IgV domain, a membrane-proximal mucin domain, a stalk domain, a transmembrane domain, and a cytoplasmic tail. The IgV domain has N-linked glycosylation sites and two antiparallel chains with four conserved cysteines that form two disulfide bonds. These bonds position the CC loop towards the FG loop, creating a unique cleft [108, 109]. The mucin domain, which is small and rich in serine, threonine, and proline, contains O-linked glycosylation sites. The stalk domain, containing N-linked sugar sites, separates the mucin and transmembrane domains.

Gal-9 is a ligand for TIM-3, binding to specific carbohydrate motifs on the TIM-3 IgV domain. This interaction triggers calcium fluxes, leading to apoptosis in Th1 cells [108]. Modeling studies have shown that a bi-antennary N-glycan at position N78 on TIM-3 does not interfere with drug binding in an extended conformation. However, the glycan can interact with a triazoloquinazolinone derivative in a folded conformation to stabilize the drug-TIM-3 complex. The non-fucosylated glycan at position N78 enhances drug interaction through an additional hydrogen bond with the α3-mannose residue [110].

Unlike other immune checkpoints, TIM-3 does not have ITIM signaling motifs in its cytoplasmic tail. However, it contains five tyrosine residues, with two conserved tyrosines in humans (Y265 and Y272) and mice (Y256 and Y263) [111–113]. These tyrosine residues are part of Src homology 2 (SH2) domain-binding motifs, which interact with various SH2 domain-containing kinases such as Fyn, HLA-B-associated transcript 3 (Bat3), lymphocyte-specific protein tyrosine kinase (Lck), PI3K p85, and IL-2-inducible T-cell kinase (ITK) [113, 114]. Unlike TIM-3, TIM-4 lacks a C-terminal cytoplasmic tail with a conserved tyrosine-based signaling motif [115]. Additionally, TIM-3 can undergo ectodomain shedding by enzymes called ADAM (A disintegrin and metalloprotease), generating a soluble form of TIM-3. ADAM10 and ADAM17 are the primary enzymes responsible for this shedding, as shown by experiments using ADAM-specific inhibitors, the ADAM10 pro-domain in HEK-293 cells, and ADAM10/ADAM17-deficient murine embryonic fibroblasts [107]. TIM-3 plays a crucial role in immune regulation. It downregulates T-cell responses by inducing cell death or exhaustion and enhances macrophage pathogen clearance by binding to Gal-9. The soluble form of TIM-3, produced by ADAM enzyme cleavage, further enhances its regulatory functions in the immune system.

### Signaling pathways of TIM-3

TIM-3 is expressed on various immune cells, including CD4^+^ Th1, CD8^+^ CTL, IL-17-producing CD4^+^ effector cells (Th17), tumor-infiltrating T-cells (TILs), Tregs, and innate immune cells. It is also expressed by tumor cells [106, 116–120]. Several ligands interact with different regions of TIM-3: Gal-9 [121] and HMGB1 [122] are soluble ligands, while phosphatidylserine (PtdSer) [123] and carcinoembryonic antigen cell adhesion molecule 1 (CEACAM1) are surface ligands [124].

Upon binding of TIM-3 to its ligands (Gal-9 and CEACAM1), the Tyr256 and Tyr263 residues are phosphorylated by Src kinases or ITK, releasing Bat3 into the cytoplasm [113, 114, 124]. This release of Bat3 enables Fyn tyrosine-protein kinase to bind to the TIM-3 cytoplasmic tail, initiating inhibitory signaling. This process produces T-cell anergy by activating the transmembrane protein phosphoprotein membrane anchor with glycosphingolipid microdomain 1. This leads to the phosphorylation of Lck by recruited tyrosine kinase, ultimately suppressing T-cell activity [111, 125]. Another inhibitory signaling mechanism involves the co-localization of TIM-3 with receptor phosphatases CD45 and CD148 at the immunological synapse, which is enhanced in the presence of Gal-9 [112]. Courtney M. Smith et al. reported that short-term binding of TIM-3 to phosphatidylserine activates T-cell receptor (TCR) signaling. Further data showed that the inhibitory activity of TIM-3 or Gal-9 is mediated by Gal-3, which bundles TIM-3 and blocks its binding to PS [126]. However, the interaction between TIM-3 and PS in NK cells abrogated cytokine production and overall activity [127]. Therefore, targeting TIM-3 promotes T-cell proliferation, activation, and cytokine production, ultimately leading to immune activation [19, 128].

### Therapeutic targeting TIM-3 in BC

ICIs have significantly transformed cancer treatment. However, these benefits are often limited to a small group of patients, resulting in relatively low response rates [19]. TIM-3 is notably expressed in both immune cells and tumor cells. Its expression is higher in basal-like and HER2^+^ BC than in healthy breast tissue [120, 129–131]. In TNBC, TIM-3 is co-expressed on CD4^+^ CD25^+^ T-cells and CD4^+^ CD25^+^ FoxP3^+^ Helios^+^ Tregs, contributing to resistance against PD-1/PD-L1 inhibitors [120]. Researchers found that overexpression of TIM-3 activates the NF-κB/STAT3 signaling pathway. This leads to changes in the expression of genes like matrix metalloproteinases-1 (MMP1), VEGF, CCND1, c-Myc, TWIST, and E-cadherin and affects tight junction (TJ) dynamics by downregulating occludin, ZO-1, and ZO-2. These changes result in increased tumor cell proliferation, invasion, migration, deterioration of TJ function, and resistance to the drug paclitaxel [132]. Epigenetic modifications, such as DNA methylation and histone modifications, also play a role in the upregulation of immune checkpoints in BC. CpG motifs in the promoter regions of immune checkpoint genes like PD-1, CTLA-4, and TIM-3 are less methylated in tumor tissue than in normal tissue. Additionally, repressive histones such as H3K9me3 and H3K27me3 are reduced in the promoter regions of the PD-1, CTLA-4, TIM-3, and LAG-3 checkpoints [133].

Given the role of TIM-3 in BC, particularly in TNBC, inhibiting this checkpoint has emerged as a promising therapeutic strategy [134]. RNA sequencing studies indicate that blocking TIM-3 enhances immune cell proliferation and activation, increases T-cell cytotoxicity, and enhances anti-tumor immunity by upregulating pathways involved in acetylation, cell differentiation, immune response, apoptosis, and TGF-β signaling. This inhibition also suppresses tumor growth, metastasis, and angiogenesis by downregulating genes linked to cell proliferation, transcriptional regulation, JAK-STAT and Wnt signaling, and integrins [19]. Research has also shown that TIM-3 is overexpressed on γδ T-cells, leading to dysfunction and increased sensitivity to apoptosis. In vitro studies indicate that TIM-3 inhibitors, α-TIM-3 or TIM-3-Fc, can improve γδ T-cell function. Moreover, the combination of TIM-3 inhibitors with the bispecific antibody MT110 and γδ T-cell adoptive transfusion has been shown to enhance the anti-tumor activity of adoptively transferred γδ T-cells [134].

Moreover, preclinical studies suggest that the inhibition of both TIM-3 and PD-1/PD-L1 has a synergistic effect, leading to the re-invigoration of T-cell function and enhanced anti-tumor immunity [128, 135, 136]. In addition, TIM-3 blockade has also been shown to improve the efficacy of chemotherapy against BC. For example, TIM-3 is highly expressed by intratumoral CD103^+^ DCs. The administration of an anti-TIM-3 antibody increases CXCL9 expression by these DCs, enhances CD8^+^ T-cell function, and thereby boosts paclitaxel’s therapeutic effect in murine models of TNBC and luminal B-BCs [137]. Additionally, researchers found that combining paclitaxel with *Ganoderma lucidum* spores could improve tumor control by reactivating exhausted TILs through TIM-3 and PD-1 blockade [138].

Significant progress has been made in therapeutically targeting TIM-3, leading to the development of various strategies, including monoclonal antibodies, BsAbs that target both TIM-3 and PD-1, and combination therapies. These approaches are being tested in several clinical trials for solid tumors, such as MBG453 (NCT02608268), TSR-022 (NCT02817633), and LY3321367 (NCT03099109) [139–141], highlighting the importance of TIM-3 in BC therapy. For example, research by Jieqiong Liu et al. found that advanced TNBC patients with high plasma levels of TIM-3 or CTLA-4 benefited more from a combination of camrelizumab (an anti-PD-1 antibody) and apatinib (an anti-VEGFR-2 agent) [142]. Another clinical study involving BC patients showed that the combination of sabatolimab (MBG453, an anti-TIM-3 monoclonal antibody) and spartalizumab (an anti-PD-1 monoclonal antibody) was well tolerated and exhibited early signs of antitumor activity [143]. Further research has investigated the relationship between TIM-3 expression and neoadjuvant chemotherapy (NAC) response in patients with locally advanced TNBC. TIM-3 expression on TILs was found to be inversely correlated with NAC response. Patients with negative TIM-3 expression had a complete pathologic response, while those with TIM-3^+^ expression had poorer chemotherapy outcomes [144]. However, Hongling Liang et al. reported that TIM-3^+^ CD8^+^ cells did not correlate with a complete pathologic response to neoadjuvant therapies. Instead, PD-1^+^ CD8^+^ cells were identified as the main predictive factor among tumor-infiltrating T-cells in BC patients [145]. These findings suggest that combining ICIs with TIM-3 blockade may enhance the effectiveness of chemotherapy in BC treatment.

### Programmed cell death protein 1 (PD-1) and its ligand

#### PD-1 structure and mechanisms of action

Programmed cell death protein 1 (PD-1) is a type I transmembrane glycoprotein receptor composed of 288 amino acids belonging to the CD28 superfamily. Structurally, PD-1 includes an IgV-like extracellular domain, a stalk domain, a transmembrane domain, and a cytoplasmic tail that contains the ITIM and ITSM [146–148]. PD-1 is expressed on various cells, including activated monocytes, B cells, T-cells, NK cells, Tregs, and myeloid DCs, but not naïve T-cells [149, 150]. In addition, PD-L1, known as CD274 or B7-H1, is a transmembrane glycoprotein that is part of the B7 family. It is expressed in various cells, including monocytes, B lymphocytes, T lymphocytes, myeloid APCs, normal epithelial cells, and cancer cells [146, 151, 152]. PD-L1 features two extracellular domains, IgV – and IgC–like extracellular domains, and a transmembrane domain. Its cytoplasmic domain is short, and whether it transmits intracellular signals remains debatable [146]. When PD-1 binds to its ligand PD-L1, it leads to the phosphorylation of tyrosine residues in the ITIM and ITSM motifs, recruiting Src homology region 2 domain-containing phosphatases 2 [153]. This alteration in the signaling pathway inhibits T-cell activation and cytokine production, causing exhaustion and apoptosis of tumor-specific T-cells. The overexpression of PD-L1 in tumor cells leads to a decreased immune response and CD8^+^ T-cell exhaustion, promoting tumor cell escape from the immune system [154–156]. Figure 3 illustrates the role of PD-L1 in immune evasion in BC. By binding to PD-1 on T-cells, PD-L1 suppresses immune responses, contributing to cold tumors. Targeting PD-L1 with ICIs can help convert cold tumors into hot, immune-responsive ones.

### Monotherapy with PD-1/PD-L1 inhibitors in BC

Several clinical studies have explored the effects of monotherapy with anti-PD-1/PD-L1 drugs in BC, with promising results mainly when administered in early disease stages. TNBC is more immunogenic than other BC subtypes due to high PD-L1 expression, TIL density, and mutation rates. As a result, clinical trials for PD-1/PD-L1 blockers have primarily focused on TNBC. Several studies have demonstrated that monotherapy with PD-1 or PD-L1 inhibitors (e.g., atezolizumab as anti-PD-L1 or pembrolizumab as anti-PD-1) in metastatic TNBC has yielded unsatisfactory results, with phase I and II clinical trials reporting objective response rate (ORRs) and median progression-free survival (PFS) of 5–20% and approximately two months, respectively [157–163] as discussed below.

The KEYNOTE-012 study (NCT01848834), a phase Ib trial initiated in May 2013, assessed pembrolizumab in TNBC patients. Of 111 patients, 58.6% had PD-L1^+^ tumors. Among 27 evaluable patients, the ORR was 18.5%, with a median response duration of 17.9 weeks. Grade 3 or higher toxicities occurred in 15.6% of patients, including one treatment-related death. This study indicated that pembrolizumab is clinically active with a manageable safety profile for advanced TNBC patients [161]. Building on these findings, the KEYNOTE-086 study (NCT02447003) further evaluated pembrolizumab in PD-L1^+^ metastatic TNBC patients. In cohort A, the ORR was 5.3% overall and 5.7% in PD-L1^+^ patients, with median PFS and overall survival (OS) of 2 and 9 months, respectively. No treatment-related deaths were observed [158]. Cohort B, which included patients who had received prior neoadjuvant therapy, showed a higher ORR of 21.4% and a disease control rate (DCR) of 23.8%, with a median duration of response (DoR) of 10.4 months. These results suggest that pembrolizumab demonstrated notable anti-tumor activity with a manageable safety profile in TNBC patients [159].

In addition, the potential of pembrolizumab was also explored in ER^+^, HER2^−^ BC through the KEYNOTE-028 study (NCT02054806). In this cohort of 25 PD-L1^+^ patients, an ORR of 12% was observed, with a median DoR of 12 months. Despite grade 3–4 adverse events (AEs) in 64% of patients, no treatment-related discontinuations or deaths were reported, indicating a tolerable safety profile [162]. In parallel, the phase I trial (NCT01375842) initiated in June 2011 explored atezolizumab in metastatic TNBC patients. The trial revealed an ORR of 12% in PD-L1^+^ Tregs patients and 6% in PD-L1^−^ patients, with a median PFS of 1.4 months and an OS of 17.6 months. Notably, higher PD-L1 expression was associated with improved ORRs and extended OS, suggesting that PD-L1 status could be a predictive marker for better outcomes with atezolizumab [160].

Moreover, a phase Ib study (NCT01772004) assessed the anti-tumor efficacy of avelumab in 168 patients with metastatic BC who received a 10 mg/kg dose bi-weekly for 6–15 months. The study reported a 3% ORR, with higher responses in PD-L1^+^ tumors (16.7% vs. 1.6% in PD-L1- tumors). Notably, 27.9% of evaluable patients experienced tumor shrinkage, and the DCR was 28.0%. In TNBC patients, the ORR was 5.2%, tumor shrinkage was 45.7%, and the DCR was 31.0%. The treatment was generally well-tolerated, with grade ≥ 3 treatment-related AEs in 13.7% of patients, including two deaths. These findings suggest that PD-L1 expression correlates with improved clinical responses [163]. Additionally, a phase III trial is ongoing to evaluate nivolumab as adjuvant or post-neoadjuvant treatment in high-risk TNBC patients (NCT02926196). The exploration of PD-L1 targeting continued with the phase I trial of envafolimab in advanced solid tumors; patients received doses ranging from 0.1 to 10.0 mg/kg weekly. The study found no dose-limiting toxicities. AEs were reported in 75.3% of patients, with 24.0% experiencing immune-related reactions such as thyroid disorders and rash. The objective response rate was 11.6%, and the DCR was 43.1%, with a median response duration of 49.1 weeks. Overall, envafolimab demonstrated a favorable safety profile and promising preliminary antitumor activity (NCT02827968) [164].

### Combination therapy of PD-1/PD-L1 inhibitors with other agents

The combination of PD-1/PD-L1 inhibitors with other therapeutic agents has been extensively studied to enhance treatment effectiveness compared to monotherapy in BC. Various strategies, including chemotherapy, radiation therapy, and targeted therapies, are discussed here, with key clinical trials and their outcomes emphasized. Table 1 summarizes key clinical trials that have evaluated ICIs in BC, with a primary focus on TNBC.

**Table 1:** Clinical trials were performed to target immune checkpoints to treat BC

Study/Trial	BC Subtype	ICIs type	Intervention	Markers	Animal or Human	Findings	Outcome	Reference
Atezolizumab + nab-paclitaxel (IMpassion130)	TNBC	PD-L1 inhibitor	Chemotherapy	PD-L1^+^	Human	Improved PFS and OS in PD-L1^+^ TNBC patients	PFS: 7.5 months vs. 5.0 months; OS: 25.0 months vs. 15.5 months	[102, 103]
Durvalumab + anthracycline & taxane	TNBC	PD-L1 inhibitor	Chemotherapy	PD-L1^+^	Human	pCR 53.4% in durvalumab group vs. 44.2% in placebo	Increased response in PD-L1^+^ TNBC	[165]
Pembrolizumab + capecitabine or paclitaxel	TNBC	PD-1 inhibitor	Chemotherapy	PD-L1^+^	Human	ORR 43% (capecitabine) vs. 25% (paclitaxel)	Manageable AEs, reduced T-cell prevalence	[166, 167]
Pembrolizumab + radiotherapy (TONIC trial)	TNBC	PD-1 inhibitor	Radiotherapy	TILs, CD8^+^	Human	Enhanced TILs and CD8^+^ T-cells	Increased ORR: 33% vs. 18.5%	[169]
Leramilimab (anti-LAG-3) + spartalizumab (anti-PD-1) + carboplatin	TNBC	PD-1 inhibitor + LAG-3 inhibitor	Chemotherapy	LAG-3^+^, PD-1^+^	Human	ORR 32.4%	Increasing side effects, promising dual inhibition	[171, 172]
Durvalumab + tremelimumab (NCT02536794)	TNBC	PD-L1 + CTLA-4 inhibitors	Immune checkpoint inhibition	PD-L1^+^, CTLA-4^+^	Human	TNBC patients: ORR 43%	Better response in TNBC than ER-positive BC	[175]
Famitinib + camrelizumab + nab-paclitaxel	TNBC	PD-L1 inhibitor + multi-target therapy	Chemotherapy, VEGFR inhibitor	PD-L1^+^, VEGFR^+^	Human	ORR 81.3%, PFS 13.6 months	Well tolerated, PD-L1^+^ patients showed better response	[176]
XmAb20717	TNBC	PD-1 + CTLA-4 inhibitors	Immune checkpoint inhibition	PD-1^+^, CTLA-4^+^	Human	Targeting advanced solid tumors		[150]
TIGIT + PD-1 inhibition (preclinical)	TNBC	PD-1 + TIGIT inhibitors	Immunotherapy	TIGIT^+^, PD-1^+^	Mouse	Increased CD8^+^ T-cell proliferation	Tumor rejection in murine models	[177, 178]
Nivolumab + OCT4/SOX2 CTLs (preclinical)	BC	PD-1 inhibitor + T-cell therapy	Stem-like cells, T-cells	OCT4, SOX2	Mouse	Enhanced cytotoxic activity against BCSC	Potential therapeutic for drug-resistant BC	[179]
Vaccines + PD-1/PD-L1 inhibitors	BC	PD-1/PD-L1 inhibitors + vaccines	Immune checkpoint inhibition	CD4^+^, CD8^+^	Mouse	Enhanced T-cell responses	Activation of CD4 and CD8 responses before therapy	[180]

Abbreviations list: AE: Adverse Events; BC: Breast Cancer; BCSC: BC Stem Cells; CD4+/CD8+: Cluster of Differentiation 4 / Cluster of Differentiation 8; CTL: Cytotoxic T Lymphocyte; CTLA-4: Cytotoxic T-Lymphocyte-Associated Protein 4; ICI: Immune Checkpoint Inhibitor; LAG-3: Lymphocyte-Activation Gene 3; ORR: Objective Response Rate; OS: Overall Survival; pCR: Pathologic Complete Response; PD-1: Programmed Cell Death Protein 1; PD-L1: Programmed Cell Death-Ligand 1; PFS: Progression-Free Survival; TIGIT: T-cell Immunoreceptor with Ig and ITIM domains; TILs: Tumor-Infiltrating Lymphocytes; TNBC: Triple-Negative BC; VEGFR: Vascular Endothelial Growth Factor Receptor

In March 2019, atezolizumab received FDA approval for use with nab-paclitaxel for treating TNBC, based on the IMpassion130 study (NCT02425891) [102, 103]. In a phase III trial involving patients with unresectable locally advanced or metastatic TNBC, the combination of atezolizumab and nab-paclitaxel was compared to nab-paclitaxel with placebo. The study found that atezolizumab significantly improved PFS in both the intention-to-treat population (7.2 months vs. 5.5 months) and the PD-L1^+^ subgroup (7.5 months vs. 5.0 months). The median OS was 21.3 months with the combination treatment and 17.6 months with placebo, with the OS in the PD-L1^+^ subgroup being 25.0 months versus 15.5 months [104]. In addition, in a double-blind, placebo-controlled phase II trial (NCT02685059), the complete response (CR) of durvalumab, when combined with neoadjuvant anthracycline and taxane-based therapy, was investigated in 174 participants, 87% of whom had PD-L1^+^ TNBC. The pCR was 53.4% in the durvalumab group compared to 44.2% in the placebo group. PD-L1^+^ patients treated with durvalumabresponded significantly more than those receiving placebo. The study concluded that concurrent administration of durvalumab with chemotherapy, particularly when durvalumab was given before chemotherapy, resulted in a favorable pCR. Thyroid dysfunction was the most common immune-related AE, affecting 47% of patients [165].

In a phase Ib trial (NCT02734290) involving 29 patients with metastatic TNBC, pembrolizumab was combined with either capecitabine or paclitaxel. Patients received 200 mg pembrolizumab every three weeks, alongside a daily oral dose of 4000 mg capecitabine or a weekly intravenous infusion of 80 mg/m² paclitaxel. The capecitabine group showed an ORR of 43%, with five partial responses (PRs), two stable disease (SD) cases, and one CR. The paclitaxel group exhibited a 25% ORR, including 1 CR, 1 PR, and 3 cases of SD. Both combinations were associated with manageable AEs, and the study noted a reduction in T-cell prevalence, indicating drug efficacy [166, 167]. In addition, the phase II TONIC trial demonstrated that radiation therapy increased TILs and CD8^+^ T-cells within the TNBC TME, enhancing the TME’s immunological activity and responsiveness to PD-1 inhibitors [168]. Similarly, in another phase II trial, the combination of pembrolizumab and radiotherapy achieved a 33% ORR, surpassing the 18.5% ORR observed with pembrolizumab monotherapy [169].

When combined with PD-1/PD-L1 inhibitors, targeted therapies have shown promise in enhancing immune response and overcoming resistance in BC treatment. PD-1 or PD-L1 inhibitors in TNBC cells induce a compensatory increase in TIM-3 and LAG-3 levels on CD4^+^ T-cells, leading to a suppressive signal that depletes effector T-cells. Early clinical trials involving anti-TIM-3 antibodies have shown an acceptable safety profile and initial signs of anticancer activity. Ongoing trials are exploring the potential benefits of combining TIM-3 inhibitors with PD-1/PD-L1 inhibitors to enhance efficacy and reduce side effects, which has been extensively reviewed [170]. In the phase I/II trial (NCT02460224), the combination of ieramilimab (anti-LAG-3 antibody), spartalizumab (anti-PD-1 antibody), and carboplatin achieved a 32.4% ORR in advanced TNBC patients, though with progressively increasing side effects. This suggests that dual inhibition of LAG-3 and PD-1 may be a promising strategy for future TNBC therapies [171, 172]. Additionally, BsAbs targeting LAG-3 and PD-1/L1 are under investigation (NCT03219268, NCT03440437).

Patients with MHC-II expression treated with pembrolizumab for TNBC demonstrated superior pCR rates and a better prognosis than MHC-II-negative patients [173]. However, MHC-II expression on BC cells exerts selection pressure on LAG-3^+^ and FCRL6^+^ TILs, antagonizing MHC-II expression and suppressing antigen presentation, which promotes adaptive resistance to anti-PD-1 therapy [174]. In addition, a 2015 study (NCT02536794) treated 18 out of 25 patients with a combination of durvalumab (anti-PD-L1 agent) and tremelimumab (anti-CTLA-4 agent). Of these patients, 11 had ER^+^ BC, and 7 had TNBC. The TNBC patients exhibited a 43% ORR, with 4 showing a sustained response within 10 months, while ER^+^ BC patients had lower response rates. These results suggest that TNBC patients may benefit more from this combination therapy despite the study’s limitations, including small sample size and single-arm design [175]. In addition, A phase II study assessed the combination of famitinib, camrelizumab, and nab-paclitaxel in advanced immunomodulatory TNBC. Among 48 patients, the objective response rate was 81.3%, with a median PFS of 13.6 months. The treatment was well tolerated, with no treatment-related deaths. PD-L1 positivity correlated with better responses. The ongoing FUTURE-SUPER trial aims to validate these findings (NCT04129996) [176]. Furthermore, ongoing phase I trials (NCT03517488) investigate vudalimab, a dual PD-1 and CTLA-4 antibody, in advanced solid tumors [150].

Simultaneous inhibition of TIGIT and PD-1 effectively hinders BC progression in murine models. In vitro studies show that this combination significantly enhances CD8^+^ T-cell proliferation specific to tumor antigens, leading to tumor rejection in murine models. In mice injected with EMT-6 BC cells, the concurrent inhibition of TIGIT and PD-1 triggered robust anti-tumor immune responses, resulting in a CR [177, 178]. One study demonstrated that a PD-1 inhibitor, nivolumab, improved the cytotoxic activity of OCT4 and SOX2 CTLs, suggesting a potential therapeutic approach for BC stem-like cells (BCSCs) in drug-resistant BC models, both in vitro and in vivo [179]. Furthermore, combining vaccines with anti-PD-1/PD-L1 agents has been proposed to enhance T-cell responses, potentially offering additional benefits when CD4 and CD8 responses are activated before checkpoint inhibitor therapy [180] Table 1.

### Cytotoxic T-lymphocyte-associated antigen 4 (CTLA-4)

#### Mechanism of action and immune activation

CTLA-4, a cell surface receptor, is expressed on activated T-cells and Treg, inhibiting T-cell function. CTLA-4 plays a crucial role in modulating immune responses in TNBC by influencing tumor immune evasion mechanisms. The biological role of CTLA-4 expression in TNBC cells underscores its potential as a therapeutic target for managing the disease. Immunohistochemistry analysis of 50 TNBC tissue samples evaluated CTLA-4 expression, scoring them as TC0 (< 1%), TC1 (1–5%), TC2 (5–50%), and TC3 (> 50%). Fisher’s Test revealed no significant association between CTLA-4 expression and tumor stage, smoking history, or chemotherapy. In addition, membrane CTLA-4 expression was observed in 90% of HCC70, HCC1937, and MDA-MB-231 cells and only 4.3% of DU4475 cells. Further evaluation proved a correlation between CTLA-4 expression and its primary ligands. High CTLA-4 expression correlated with upregulation of CD86 (73%) and CD80 (68%), while tumors with low CTLA-4 expression showed lower CD86 (34%) and CD80 (38%) upregulation. In addition, CTLA-4 blockade activated AKT signaling by phosphorylating Ser473 and Thr308 in HCC1937 cells but not in MDA-MB-231 cells.

In contrast, ERK1/2 was phosphorylated in HCC1937 cells following CTLA-4 activation but remained unchanged in MDA-MB-231 cells. CTLA-4 blockade reduced the proliferation of HCC1937 and MDA-MB-231 cells in a dose-dependent manner at 48 h, while CD80 altered cell numbers only at 24 h in MDA-MB-231 cells. IL-2 secretion was significantly reduced in HCC1937 cells treated with CD80, while Ipilimumab markedly enhanced IL-2 secretion in these cells. MDA-MB-231 cells were unaffected by either treatment [181]. Figure 3 shows the role of CTLA-4 in immune suppression in BC. CTLA-4 inhibits T-cell activation, promoting immune evasion in cold tumors. Targeting CTLA-4 with inhibitors can boost T-cell activity, potentially transforming cold-to-hot tumors, where a more effective immune response can be triggered.

### Clinical trials and combination therapies in BC

The CTLA-4 studies as monotherapies and in combination with other treatments are summarized in Table 2. Combination therapy with anti-PD-1 and anti-CTLA-4 antibodies reduced tumor growth in 4T1 BC models. In addition, this therapy showed significant anti-cancer efficacy compared to both the control and monotherapies in the 4T1 model. Anti-CTLA-4 treatment significantly decreased Tregs and increased the CD8^+^ T-cells/Tregs cells ratio, whereas anti-PD-1 or combinational therapy did not alter or improve these ratios. In addition, clinical data indicated longer relapse-free survival (RFS) for BC patients receiving the combination therapy [182]. In addition, differential responses were observed following 24-hour incubation of MDA-MB-231 and MCF-7 BC cells with peripheral blood mononuclear cells (PBMCs) alone or in combination with CTLA-4 or PD-1 inhibitors. CTLA-4 inhibition resulted in a notable reduction in MDA-MB-231 proliferation, while MCF-7 cells remained unaffected. Additionally, G1/S-phase arrest in MDA-MB-231 cells was observed with PBMC + CTLA-4 inhibitor and PBMC inhibitor, whereas MCF-7 cells showed only a slight increase in G1-phase cells with PBMC + PD-1 inhibitor. Immune checkpoint protein analysis revealed dominant PD-L1 and PD-1 expression in MDA-MB-231 and MCF-7 cells, respectively. MCF-7 cells also expressed CD86. In CD8^+^ lymphocytes, PD-1 and CD80 were predominant. CTLA-4 or PD-1 inhibition significantly reduced IFN-gamma production in CD4^+^ lymphocytes and decreased IL-10 production in CD8^+^ lymphocytes. Anti-PD-1 treatment enhanced GZMB and PRF1 production in PBMC co-cultured with MDA-MB-231 cells [183].

**Table 2:** Preclinical CTLA-4 monotherapy and combination therapy in BC

BC subtype	ICIs type	Intervention	Markers	Cell lines	Animal or human	Refrence
TNBC	anti-CTLA-4, anti-PD-1	Anti-PD-1	CD45^+^, CD4^+^, CD25^+^, FoxP3^+^, CD8^+^	CT26, 4T1	6 week-old female BALB/cJRj or C57BL/6JRj mice	[183]
BC	anti-CTLA-4, anti-PD-1	Anti-PD-1	CD3, CD4, CD8, CD86, CD80	MDA-MB-231, MCF-7, PBMC	-	[184]
TNBC	ICIs	-	CD4^±^, CD25^±^, FoxP3^+^, Helios	MDA-MB-231, MDA-MB-468, MCF-7, PBMCs	-	[120]
TNBC	CTLA-4	-	CD80, CD86, CD4, CD8	MDA-MB-231, HCC1937, DU4475, HCC70	50 patients diagnosed with invasive TNBC	[182]
BC	CTLA-4 mAb	-	CD4, CD8	MDA-MB-231, SKBR3, MCF-7, T47D	-	[185]
BC	CTLA-4 blockade (C4)	HT and UnHT	-	4T1	6 to 8 weeks old male BALB/cAJcl mice	[186]
TNBC	anti-CTLA-4 + MUC1 mRNA nanovaccine	MUC1 mRNA nanovaccine	Treg, CD8^+^	4T1	6 to 8 weeks old female BALB/c mice	[278]
TNBC	anti-CTLA-4 + MUC1 mRNA Nano-vaccine	MUC1 mRNA Nano-vaccine	CD28, CD26	4T1	6 to 8 weeks old female BALB/c mice	[279]
TNBC	anti-CTLA-4 + Murine TMV vaccine	Murine TMV vaccine	CD80, CD4, CD8, IL12	MDA-MB-231, MDA-MB-453, BT-549, HCC-1187, Jurka, t E6.1, NK-92, NK-92MI	6 to 8 weeks old Female BALB/cJ mice	[342]
BC	CTLA-4 mAb + 5-aza-2’-deoxycytidine (5DC)	5‑aza‑2’‑deoxycytidine (5DC)	CD86, CD80, CD83^+^, CD1a^+^	MCF-7, MDA-MB-453, MDA-MB-231, BT549	-	[244]
HER2 BC	anti-CTLA-4 + trastuzumab deruxtecan (DS-8201a)	Trastuzumab deruxtecan (DS-8201a)	CD45, CD8, CD4	KPL-4, CT26.WT, EMT-6	5 week old female BALB/cmice	[281]
TNBC	anti-PD-L1 + anti-CTLA-4 mAbs (ipilimumab + atezolizumab)	ipilimumab and atezolizumab	CD16	MDA-MB-231, BT-549, BT-474, MCF-7	-	[282]
BC	anti-PD-1 + anti-CTLA-4 + rAd.sT	rAd.sT	CD45, CD3e, CD4, CD8a, CD44, CD62L, CD11c^+^, CD86^+^	MCF-7, MDA-MB-231, 4T1	4 to 6 weeks old BALB/c mice	[285]
TNBC	anti-PD-L1 + anti-CTLA-4 (aPC) + rAd.GM	rAd.GM	CD4, CD8, CD3, CD25, CD44, CD62L, CD197	4T1, EMT-6, MDA-MB-231	4 to 6 weeks old BALB/c mice	[286]
BC	FoxP3, CTLA-4, and GITR	-	FoxP3, CTLA-4 and GITR	PBMCs	20 women with BC and 20 healthy women	[291]
BC	anti-CTLA-4 + RON kinase	RON kinase	CD8a, CD4, CD62L, CD80, CD86	-	4 to 6 weeks old WT (FVB) and FVB RON TK-/- female mice	[292]

**Abbreviations list**: BC, breast cancer; TNBC, triple-negative BC; ICIs, immune checkpoint inhibitors; mAb, monoclonal antibody; HT, hyperthermia; UnHT, unheated; Treg, regulatory T-cells; mRNA, messenger RNA; TMV, tumor membrane-based vaccine; PBMC, peripheral blood mononuclear cells; MUC1, mucin 1; CD, cluster of differentiation; IL12, interleukin 12; FoxP3, forkhead box P3; GITR, glucocorticoid-induced TNFR family-related gene; RON, recepteur d’origine nantais; CTX, cyclophosphamide; 5DC, 5-aza-2’-deoxycytidine; rAd.sT, recombinant adenoviral vector encoding tumor-associated antigens; rAd.GM, recombinant adenoviral vector expressing GM-CSF; MDA-MB-231, MDA-MB-468, MDA-MB-453, MCF-7, SKBR3, T47D, human BC cell lines; 4T1, murine BC cell line; BALB/c, a strain of laboratory mice; C57BL/6, a strain of laboratory mice; FVB, a strain of laboratory mice; EMT-6, mouse mammary carcinoma cell line; KPL-4, HER2-positive BC cell line; EO771, murine mammary carcinoma cell line; MCaP0008, mouse mammary carcinoma cell line; MMTV-PyVT, transgenic mouse model for BC

Furthermore, the expression dynamics of IC and Treg-related markers on CD4^+^ T-cells were studied in co-culture with MDA-MB-231 cells over 24, 48, and 72 h, using anti-PD-1 and anti-PD-L1 antibodies. The percentage of CD4^+^ PD-1^+^, CD4^+^ CTLA-4^+^, and CD4^+^ TIM-3^+^ T-cells increased over the three days following PBMC activation. The rate of CD4^+^ LAG-3^+^ and CD4^+^ FoxP3^+^ Helios^+^ T-cells increased over two days and remained stable on the third day. When activated PBMCs were co-cultured with MDA-MB-231, MDA-MB-468, and MCF-7 cells, there was an increase in the expression of PD-1, CTLA-4, LAG-3, and TIM-3 in CD4^+^ CD25 − T-cells, along with a slight upregulation of FoxP3 and Helios. A similar investigation in the CD4^+^ CD25^+^ T-cell subset showed that the three BC cell lines upregulated TIM-3 expression. PD-1 expression was downregulated in MDA-MB-231 cells but upregulated in MDA-MB-468 cells. Additionally, the co-blockade of PD-1 and PD-L1 downregulated CTLA-4 expression in CD4^+^ CD25^−^ T-cells but upregulated the co-expression of TIM-3 and LAG-3 in CD4^+^ CD25^+^ T-cells within the BC cells [120].

Additionally, studies demonstrate that CTLA-4^+^ cancer cells, mainly from BC, suppress the maturation and function of DCs, critical orchestrators of anti-tumor immunity. This suppression manifests as downregulation of MHC Class II, CD86, and HLA-DR on DCs, coupled with impaired cytokine production, ultimately hindering T-cell activation and favoring Treg induction. Significantly, blocking CTLA-4 with antibodies reverses these suppressive effects, restoring DC function, promoting T-cell responses, and directly inhibiting tumor cell viability and proliferation. This highlights CTLA-4 blockade as a promising therapeutic strategy to unleash anti-tumor immunity by targeting the CTLA-4 axis within the TME [184].

The synergistic potential of combining local hyperthermia (HT) therapy with CTLA-4 blockade has been investigated in BC models, revealing promising results. Preclinical studies demonstrated that HT therapy synergized with CTLA-4 blockade to elicit potent anti-tumor effects, significantly reducing local and distant tumor growth compared to monotherapy or untreated controls. This combined treatment modality translated to a notable survival advantage, with 39% of mice exhibiting complete tumor regression and prolonged median survival compared to CTLA-4 blockade alone. Interestingly, adding FTY720, a sphingosine-1-phosphate receptor modulator, to the HT + CTLA-4 regimen unexpectedly diminished survival, underscoring the importance of carefully evaluating combination strategies. These findings highlight the potential of integrating local HT with CTLA-4 blockade as a novel therapeutic approach for BC, warranting further investigation in clinical settings [185].

Table 2.

### Breast TME composition and immunotherapy resistance

Breast tumors across all subtypes undergo metabolic reprogramming (e.g., the Warburg effect) that alters the chemical composition of the TME [186]. Highly glycolytic tumor cells consume nutrients and release metabolic byproducts that accumulate in the TME, such as lactate, protons, and ammonia, along with ROS. This creates a hostile TME characterized by low pH, hypoxia, and oxidative stress. These conditions force infiltrating immune cells to adapt metabolically, often impairing their anti-tumor functions. As a result, an immunosuppressive milieu develops, enabling tumor immune evasion and reducing the efficacy of immunotherapies [187].

### Lactate and tumor acidosis

Elevated lactate is a hallmark of metabolically reprogrammed breast tumors (particularly in aggressive subtypes like TNBC) and a major driver of immune escape. Tumor cells convert excess glucose to lactate and export it with protons, acidifying the TME. The lactic acid accumulation directly inhibits various immune cells: CTLs and NK cells have reduced cytokine production and cytolytic activity in low pH conditions. Lactate also disrupts dendritic cell function, hampering antigen presentation and T cell priming. High glycolytic activity by tumors creates a metabolic competition that starves T cells of glucose and accumulates lactate, effectively “shutting down” T cell effector functions [186]. Indeed, lactate-rich environments prevent T cells from exporting their lactate, leading to an intracellular build-up that impairs their proliferation and IFN-γ production. Studies have shown that tumors with high lactate dehydrogenase (LDH-A) expression have fewer IFN-γ^+^ CD8^+^ T-cells, indicating suppressed Th1 responses, whereas reducing lactate production (e.g., via LDH-A inhibition) can restore T-cell infiltration and tumor killing [188, 189]. Tumor-derived lactic acid also triggers mitochondrial dysfunction and excess ROS in T cells, driving them into apoptosis [188].

In a lactate-rich TME, lactate accumulation and subsequent acidification orchestrate a multi-layered suppression of immune cell functions, contributing significantly to immunotherapy resistance in BC. For CTLs, high lactate levels disrupt the lactate gradient essential for efficient lactate efflux [190]. This leads to intracellular lactate accumulation and impairs key metabolic processes required for T cell proliferation and effector functions such as IFN-γ production. Additionally, lactate-induced acidosis interferes with TCR signaling by inhibiting transcription factors like NFAT and dampening MAPK and JNK pathways, ultimately reducing the cytotoxic capacity of these T cells [191]. NK cells are similarly affected; high extracellular lactate downregulates activating receptors such as NKp46 and NKG2D and decreases the expression of cytotoxic proteins like perforin and granzyme [192]. Invariant NKT (iNKT) cells also suffer under these conditions, as lactate-mediated acidosis suppresses mTOR signaling and prevents the nuclear translocation of PLZF, a key regulator of their activation and cytokine production, thereby compromising their survival and function [193].

When exposed to lactate, DCs, particularly plasmacytoid DCs (pDCs), exhibit reduced glycolytic capacity and maturation. This is due to lactate uptake via monocarboxylate transporters (MCTs) and signaling through receptors such as GPR81, which collectively inhibit their ability to present antigens and produce type-I interferons. As a result, T cell priming is impaired, further weakening the overall anti-tumor immune response [194, 195].

Moreover, lactate promotes the development and immunosuppressive functions of MDSCs, which inhibit T cell responses and encourage Treg expansion [54]. Tregs adapt metabolically to thrive in the low-glucose, high-lactate environment by upregulating oxidative phosphorylation, thereby maintaining suppressive functions despite metabolic challenges [196]. TAMs are also re-educated by lactate. Exposure to high lactate levels induces an M2 polarization of TAMs via lactate-sensitive receptors such as GPR132, leading to activation of ERK/STAT3 pathways. These M2 macrophages secrete immunosuppressive cytokines (e.g., IL-10), promote angiogenesis, and facilitate tissue remodeling, all of which contribute to tumor progression and the expansion of Tregs [197, 198].

These lactate-induced alterations in the TME create a hostile environment for anti-tumor immune cells and foster an immunosuppressive network that limits the effectiveness of immunotherapies. Targeting these metabolic pathways—reducing lactate production, blocking lactate transport, or modulating the cellular response to acidosis—offers a promising strategy to overcome immune resistance and enhance therapeutic efficacy in BC.

### Reactive oxygen species (ROS) and breast TME

Oxidative stress, driven by excessive ROS, plays a critical role in the development and progression of BC. Elevated ROS levels, generated through various mechanisms such as enhanced tumor cell metabolism, genetic alterations in antioxidant enzymes, estrogen metabolism, and inflammation, lead to DNA damage, promote oncogenic signaling, and facilitate angiogenesis and metastasis [199]. Tumor cells in BC produce higher amounts of ROS than normal cells, which activates key signaling pathways, including MAPK, JNK, and HIF-1α, driving cell proliferation and survival while impairing apoptosis. Additionally, ROS promotes the activation of MMPs that degrade the extracellular matrix, thus supporting tumor invasion and metastasis. Although antioxidant enzymes like superoxide dismutase (SOD) and glutathione peroxidase (GPX) are upregulated as a cellular defense mechanism, their increased activity in cancer cells can paradoxically contribute to therapeutic resistance by protecting tumor cells from oxidative damage [200]. Understanding these interconnected processes underscores the potential of targeting ROS production and redox signaling pathways to overcome resistance and improve BC treatment outcomes. ROS in the breast TME influences multiple immune cells. Excess ROS drive suppressive phenotypes in myeloid cells (e.g., macrophages, neutrophils), recruit Tregs, and inhibit CTLs, collectively creating an immunosuppressive niche.

Paradoxically, while ROS hinders effector T cells, the suppressive Treg population often endures or even benefits from the oxidative TME. Tregs are attracted to tumors by ROS-inducing inflammation and MDSC-secreted chemokines. They appear to require some ROS signaling for optimal suppressive function – experiments show that eliminating ROS (e.g., with N-acetylcysteine) can reduce Treg-mediated suppression of conventional T cells. A low-ROS state is associated with Treg “hypofunction”, whereas moderate ROS helps maintain the stability of Treg suppressive molecules (e.g., via stabilization of the redox-sensitive factor SENP3) [201]. Importantly, Tregs are more resistant to ROS-induced cell death than effector T cells, partly due to higher intrinsic antioxidant levels. This allows Tregs to accumulate in oxidative, hypoxic areas of tumors and continue curbing anti-tumor immunity. Thus, ROS indirectly bolsters tumor immune evasion by creating conditions favoring robust Treg activity alongside disabled effector T cells [202].

Excessive ROS is linked to the suppression of T cell immunity through a complex array of mechanisms within TME. High levels of ROS have been correlated with the induction of activation-induced cell death in T cells, a process mediated by mitochondrial hyperpolarization and oxidative stress that results in apoptosis and diminished function of tumor-infiltrating T cells [203]. Moreover, the increased production of ROS—exacerbated by the downregulation of mitochondrial SOD2—has been shown to impair the activity of CD8⁺ T cells, with some recovery of function observed when mitochondrial ROS are scavenged [204]. The formation and maintenance of CD8⁺ memory T cells are similarly disrupted when the pentose phosphate pathway is compromised, leading to lower glutathione (GSH) levels and a subsequent rise in ROS that interferes with memory T cell development [203]. Additionally, ROS influence other components of the immune system; for example, they promote the differentiation of Tregs, thereby contributing to an immunosuppressive environment, and are involved in maintaining the immature state of MDSCs [205]. Tumor cell-derived ROS also facilitate the migration of macrophages and their polarization toward a pro-tumoral M2 phenotype via PI3K signaling. These MDSCs and M2 macrophages further exacerbate oxidative stress by inhibiting TCR expression, suppressing T cell proliferation, upregulating immune checkpoint molecules, and releasing immunosuppressive cytokines such as TGF-β and IL-10 [206]. Additionally, ROS induces fibroblasts to differentiate into pro-tumoral myofibroblasts, which is associated with excluding lymphocytes from tumor tissues. These ROS-mediated changes within the TME collectively impair effective T-cell responses and facilitate tumor immune evasion [207].

BC TAMs are usually polarized to an M2-like, immunosuppressive state. M2 macrophages inherently produce less ROS than pro-inflammatory M1 macrophages due to lower NADPH oxidase (NOX) expression and higher antioxidant enzymes. Interestingly, moderate ROS signaling is required for M2 polarization via pathways like STAT3, as adding H2O2 can boost M2 markers. However, excessive ROS can push macrophages to further immunosuppressive activity – for example, ROS accumulation (triggered by agents like glutathione inhibitors or chemotherapy) activates NF-κB in TAMs, upregulating PD-L1 and anti-inflammatory cytokines [208]. TAM-derived ROS (such as H2O2) can directly inhibit T-cell function by downregulating the CD3ζ chain of the T-cell receptor, thereby impairing T-cell activation. Through these mechanisms, ROS-conditioned TAMs support tumor immune evasion by suppressing Th1/CTL responses and promoting a tolerant environment [209].

MDSCs are potent immunosuppressive cells in BC TME that rely on ROS as effector molecules. They generate large amounts of superoxide O2^–^, hydrogen peroxide H2O2, and peroxynitrite ONOO^–^ via NOX2 and other enzymes. These ROS/RNS species blunt anti-tumor immunity by inducing T-cell anergy and nitrating T-cell receptors and chemokines, which disables TCR signaling and impairs T-cell infiltration. Despite decades of using methods to monitor tumor markers and circulating tumor cells, the early detection of cancer recurrence remains a significant clinical hurdle. A bioinformatic comparison with a single-cell RNA sequencing dataset of MDSCs revealed that CSF3R^+^ MDSCs could potentially be predictors of tumor relapse. These observations were confirmed with human peripheral blood mononuclear cell PBMC datasets, which showed that patients with elevated CSF3R levels had a stronger MDSC gene signature and poorer survival outcomes. In vitro experiments demonstrated that CSF3R^+^ MDSCs exhibited higher ROS production and robust T-cell suppressive activity [210]. However, the role of immunosuppression in TNBC was examined, and the potential of combining doxorubicin with glyceryltrinitrate (GTN), a nitric oxide (NO) donor, to overcome chemotherapy resistance was investigated. In a TNBC mouse model, enhanced anti-tumor efficacy was observed when the combination treatment was administered, as evidenced by increased intra-tumoral recruitment and activation of CD8⁺ T cells, along with a reduction in the immunosuppressive function of polymorphonuclear-MDSCs (PMN-MDSCs). Mechanistically, a ROS-dependent cleavage of STAT5 was induced by GTN, with or without doxorubicin. It is proposed that GTN, in combination with chemotherapeutic agents, should be further evaluated as an adjuvant therapy for TNBC patients experiencing treatment failure [211].

Neutrophils in the TME can adopt an N2 phenotype under tumor-derived signals. These TANs release ROS that contribute to immune suppression. Neutrophil-derived H2O2 has been shown to reduce CD3ζ expression on T cells, impairing T-cell receptor signaling and cytokine production. The same H_2_O_2_ can inhibit NK cell cytotoxic functions, diminishing tumor cell clearance. Additionally, neutrophils can undergo the formation of neutrophil extracellular traps (NETs) or NETosis in a ROS-dependent manner, which may trap tumor-specific T-cells or sequester cytokines, further contributing to an immunosuppressive environment. Through ROS and NETs, TANs help foster tumor progression by restraining effective T and NK cell activity in the BC TME [212, 213].

Overall, ROS orchestrates a shift in the breast TIME toward an immunosuppressive, pro-tumor state. Elevated ROS levels drive myeloid cells (macrophages, neutrophils, MDSCs) to suppress immunity and promote Tregs while directly incapacitating CTLs. This redox-mediated immune dysfunction is a hallmark of aggressive tumors and sets the stage for resistance to therapy.

### Ammonia accumulation in the TME

Ammonia, traditionally viewed as a toxic metabolic byproduct, is recycled in BC cells to support tumor biomass. In these cells, ammonia is assimilated primarily through glutamate dehydrogenase (GDH)–mediated reductive amination, converting ammonia and α-ketoglutarate into glutamate. This process facilitates the synthesis of other amino acids, such as proline and aspartate, thereby maximizing nitrogen utilization and accelerating cancer cell proliferation. The knockdown of GDH led to a marked decrease in the incorporation of ammonia into key metabolites, underscoring the enzyme’s pivotal role in ammonia recycling. These findings suggest that ammonia recycling is an efficient process that supports BC growth and may represent a novel metabolic vulnerability for therapeutic intervention [214].

Additionally, a recent study showed that ammonia accumulation in the TME induces T cell exhaustion in colorectal cancer. Elevated ammonia levels were found to impair T cell proliferation and activation, as demonstrated by reduced expression of activation markers such as CD25 and increased levels of exhaustion markers including PD-1 and TIM-3 on both CD4⁺ and CD8⁺ T cells. The transsulfuration pathway in T cells was disrupted by ammonia, resulting in diminished glutathione synthesis and increased oxidative stress, which was found to contribute to mitochondrial dysfunction and decreased oxygen consumption—in vitro experiments revealed that T cells treated with ammonia exhibited reduced proliferation and increased apoptosis. Furthermore, the administration of ornithine—a compound that stimulates ammonia clearance via the urea cycle—was shown to lower serum ammonia, restore T cell proliferation, and reduce tumor growth, thereby enhancing the response to immunotherapy.

Collectively, it is suggested that TME ammonia contributes to immunosuppression by reprogramming T cell metabolism and promoting T cell exhaustion and that strategies aimed at neutralizing ammonia may improve the efficacy of immunotherapies in colorectal cancer [215]. However, the role of ammonia in breast TME remains unknown, and further research is required.

### Immunosuppressive cytokines and breast TME

In BC, immunosuppressive cytokines are critical in creating a TME that hinders effective immunotherapy. Key cytokines—IL-10, TGF-β, and IL-35—are major contributors to this immunosuppressive milieu, each exerting distinct yet overlapping effects that collectively impair anti-tumor immune responses [216, 217].

IL-10 is widely produced by BC cells and by TAMs, MDSCs, and Tregs within the TME. In breast tumors, elevated IL-10 levels have been linked to poor clinical outcomes due to their ability to inhibit the function of CTLs [218, 219]. IL-10 signals through its receptor to activate STAT3, reducing the secretion of pro-inflammatory cytokines and diminishing T cell proliferation. This leads to an exhausted T cell phenotype marked by increased expression of inhibitory receptors such as PD-1 [220, 221]. Furthermore, IL-10 promotes the expansion and stabilization of Tregs, which further suppresses effector T-cell responses. Therefore, high IL-10 levels in BC are associated with low T-cell infiltration and reduced responsiveness to immune checkpoint blockade therapies [222].

TGF-β is another prominent immunosuppressive cytokine that is frequently upregulated in BC. Secreted by tumor cells and stromal elements, including fibroblasts and TAMs, TGF-β exerts multiple inhibitory effects on the immune system. In breast tumors, TGF-β directly impairs the proliferation and cytotoxic function of CD8⁺ T cells while promoting their conversion into a dysfunctional, exhausted state. TGF-β also drives the differentiation of naïve T cells into Tregs, enhancing the suppressive capacity of the TME [216, 223]. Tregs play a vital role in suppressing the activity of various immune cells—including CD8⁺ and CD4⁺ T cells, dendritic cells, and NK cells. Targeting key markers such as CD25, FoxP3, the TGF-β receptor, IDO-1, ARG1, and GLS shows promise for enhancing antitumor immunity [224].

Besides, it was also found that TGF-β activates macrophages to adopt an M2-like tumor-associated phenotype, and reciprocally, RAD18 is further activated in TNBC cells by TGF-β released from these macrophages, thereby forming a positive feedback loop that enhances tumor stemness. This loop was disrupted by the inhibition of YAP or TGF-β, reducing cancer stemness and proliferation. In vivo, tumor growth was promoted by RAD18, and RAD18 increased the recruitment of M2-type macrophages in nude mice. The RAD18-YAP-TGF-β loop is highlighted as a critical driver of tumor stemness in TNBC and is identified as a potential therapeutic target [225]. Accordingly, TGF-β influences the phenotype of macrophages by skewing them towards an M2, pro-tumoral state, which supports tumor progression and metastasis [226]. In addition, high TGF-β activity in BC has been shown to create a physical and biochemical barrier that limits T cell infiltration and activity, thereby contributing to resistance against therapies such as PD-1/PD-L1 inhibitors [227].

IL-35, a relatively new addition to immunosuppressive cytokines, has also been implicated in BC progression [228, 229]. An inverse correlation was observed in hepatocellular carcinoma patients between the frequency of checkpoint inhibitor-positive Tregs and patient age, which was associated with elevated numbers of Tregs producing IL-10 and IL-35. It was demonstrated that IFN-γ secretion and the cytotoxicity of CD8⁺ T cells were suppressed by Tregs, as assessed by a lectin-dependent cellular cytotoxicity assay using checkpoint inhibitor-negative P815 target cells. Furthermore, the inhibition of IFN-γ secretion induced by Tregs was partially reversed by neutralizing PD-1 and PD-L1 antibodies, specifically in HCC patients. It was concluded that in HCC, immune checkpoint molecules are upregulated by peripheral Tregs, and immunosuppressive cytokines such as IL-35 are secreted by these cells, thereby contributing to systemic immune dysfunction and the suppression of antitumor activity, which may facilitate tumor development at a younger age. Finally, it was found that blocking PD-L1/PD-1 interactions selectively interfered with these inhibitory Treg–T effector cell interactions, resulting in enhanced antitumor activity even against tumor cells lacking checkpoint inhibitor expression [230]. Moreover, it was demonstrated that IL-35, produced primarily by regulatory T and B cells, is also expressed and secreted by BC cells. Higher levels of IL-35 in BC cells were closely associated with poor patient prognosis and were identified as an independent unfavorable prognostic factor. It was further shown that BC cell-derived IL-35 inhibited the proliferation of conventional T cells and induced their conversion into IL-35-producing induced Tregs (iTreg35) cells. In addition, BC cell-derived IL-35 was observed to promote the secretion of the inhibitory cytokine IL-10 while significantly decreasing the production of Th1-type IFN-γ and Th17-type IL-17, and it was associated with an elevated expression of the inhibitory receptor CD73 on conventional T cells. The exhaustion of conventional T cells and the induction of iTreg35 were found to be mediated through the activation of transcription factors STAT1 and STAT3. These findings indicate that IL-35 produced by BC cells promotes tumor progression by suppressing tumor-infiltrating conventional T cells and inducing iTreg35 cells in the TME, thereby representing a potential therapeutic target for BC [228].

Strategies to counteract these cytokines in BC are currently under exploration. Preclinical studies have demonstrated that blocking IL-10 or TGF-β can alleviate immune suppression and restore CTL function, especially when combined with ICIs [231]. Similarly, targeting IL-35 may reduce Treg-mediated suppression and rejuvenate exhausted T cells. By neutralizing these cytokines, the breast TME can be reprogrammed from an immunosuppressive cold state to an immune-active hot state, making tumors more responsive to immunotherapy. However, the effects of IL-35 on immune checkpoints in breast TME need to be elucidated.

In summary, in BC, the combined actions of IL-10, TGF-β, and IL-35 establish a potent immunosuppressive environment that limits the effectiveness of immunotherapy. A better understanding of these cytokine-driven pathways is essential for developing combination therapies that target both the tumor cells and their suppressive microenvironment, thereby overcoming resistance and improving patient outcomes.

### CAR-T cell therapy in cold-to-hot breast tumor transition

The transition of cold-to-hot tumors is critical for improving chimeric antigen receptor (CAR) therapies in BC treatment. Many BC tumors, especially those with a high immune suppression, are initially cold, with few immune cells in the TME. This immune-excluded state makes them resistant to therapies like CAR-T cells. However, by targeting specific tumor antigens and utilizing strategies to alter the TME, CAR-T cell therapy can help “Heat-Up” these cold tumors. This process involves increasing immune cell infiltration and stimulating an immune response, enabling better tumor targeting. Researchers are actively exploring enhancing CAR-T cell efficacy by combining them with other immunotherapies, such as checkpoint inhibitors, to facilitate this transition. The ultimate goal is to turn immune-resistant, cold tumors into immune-active, hot tumors, significantly improving therapeutic outcomes for aggressive or metastatic BC patients [67].

Furthermore, Inducers of immunogenic cell death, such as chemotherapy, can stimulate the release of tumor antigens, thereby enhancing CAR-T cells’ ability to recognize and target cancer cells. Moreover, ICD-based interventions can help overcome immunosuppressive conditions and physical barriers within the TME, allowing CAR-T cells to infiltrate the tumor more effectively and exert their cytotoxic functions [33]. Additionally, studies of the cell surface proteome (the surfaceome) have unveiled numerous prospective therapeutic targets. A quantitative proteomic analysis of N-linked glycoproteins revealed notable remodeling of the surface and glycoproteome in breast epithelial cell lines. Cross-examination of transcriptomic and proteomic data from tumor and normal tissues pinpointed multiple cell surface molecules displaying elevated expression, indicating potential targets for CAR-T therapy [232].

By targeting specific antigens like glycoproteins and tyrosine kinase receptors, CAR-T cells can help break down immune resistance in cold tumors, facilitating their transition to a more immune-active, hot tumor environment. This transition is crucial for improving BC therapy outcomes, particularly in aggressive or metastatic cases. This section details the molecular aspects of specific tumor-associated antigens, such as glycoproteins, tyrosine kinase receptors, and immune-related proteins commonly overexpressed in malignant BC cells. These antigens target “Heat-Up” cold tumors and facilitate the immune response. The strategic targeting of these antigens could effectively redirect the immune system to attack cancer cells, transforming a cold tumor—characterized by limited immune activity—into a hot one where T-cells are activated and capable of attacking the cancer. We also detail the specifics of CAR-T cell engineering and how these targeted strategies differ across various BC subtypes: several subtypes, each with unique characteristics that influence how they respond to CAR-T cell therapy.

**Fig. 4:**
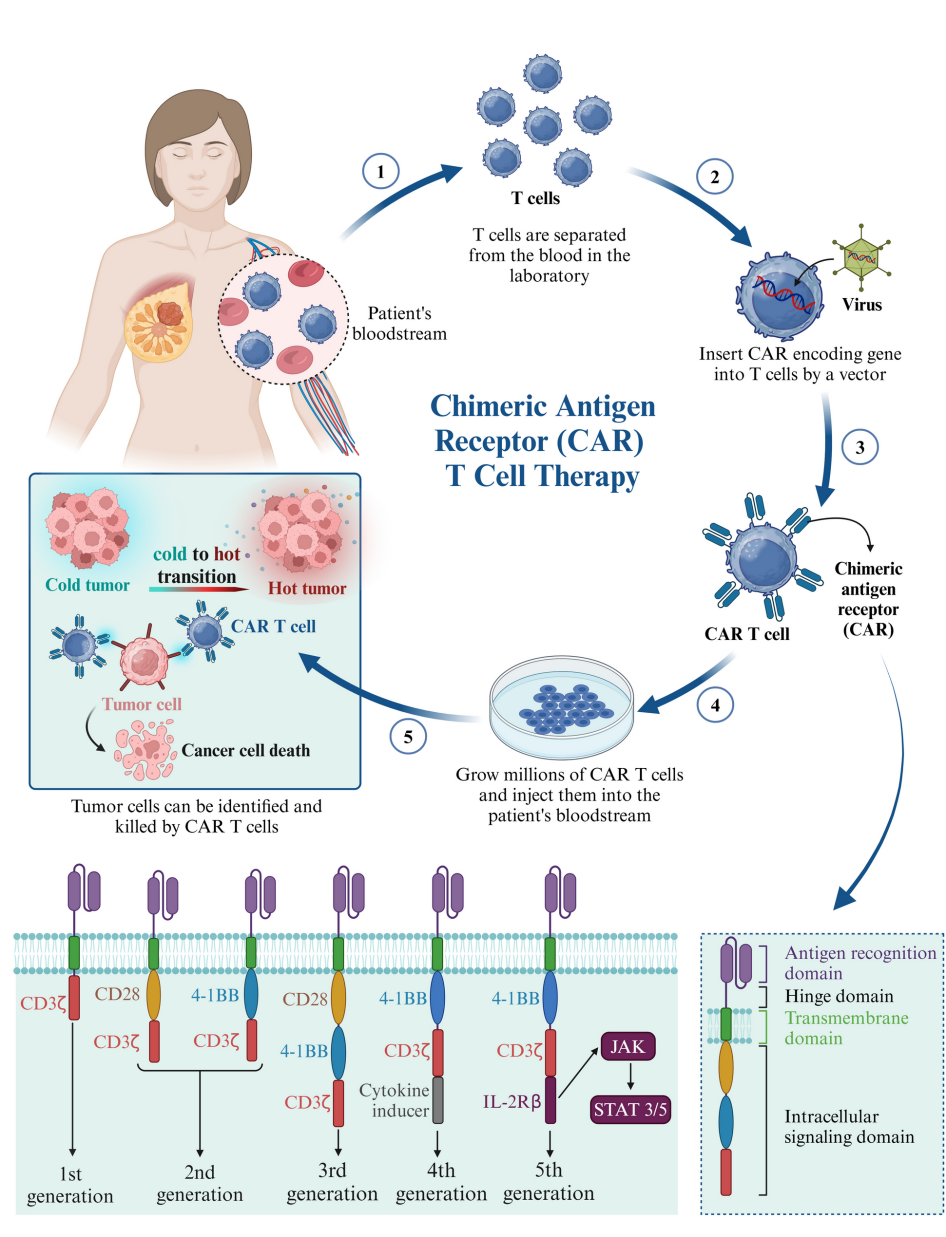
Mechanism of CAR-T cell Therapy in BC. This figure delineates the therapeutic mechanisms of CAR-T cell therapy specifically for BC treatment. It starts by illustrating the structure of the CAR, which comprises an extracellular domain that recognizes tumor antigens (such as Folate Receptor-α, MUC1, and EpCAM) and intracellular signaling domains that activate T-cells upon binding to these antigens. The figure emphasizes the targeted antigens relevant to BC, including glycoproteins and tyrosine kinase receptors, often overexpressed in tumor cells. The activation cascade that leads to T-cell proliferation and cytotoxic granule release, resulting in tumor cell lysis, is depicted. Additionally, it addresses the challenges CAR-T cells encounter within the immunosuppressive TME and outlines strategies under development to enhance their efficacy, including the incorporation of co-stimulatory signals and combination therapies. This figure provides a detailed visual overview of CAR-T cell therapy’s potential in treating different BC subtypes, underscoring ongoing research to improve patient outcomes through innovative CAR designs. *Created in BioRender. Jabbarzadeh Kaboli*,* P. (2024)*https://BioRender.com/e10t340

Figure 4 effectively illustrates the pivotal role of CAR-T cell therapy in transforming TME. It depicts the mechanism by which CAR-T cells recognize and bind to specific tumor-associated antigens, including folate receptor alpha (FRα), mucin-1 (MUC1), and epithelial cell adhesion molecule (EpCAM). This binding initiates an activation cascade within the T-cells, resulting in their proliferation and the release of cytotoxic granules that target and destroy tumor cells. Enhancing this immune response is essential for converting cold tumors into hot ones. As CAR-T cells engage these antigens on BC cells, they eliminate malignant cells and stimulate increased immune activity within the TME, thereby facilitating the “heating up” of the tumor.

### Glycoprotein targets in CAR-T cell therapy for BC

Targeting glycoproteins like CD44v6 can convert cold into hot breast tumors, improving immune recognition. CAR-T cell therapy targeting CD44v6 disrupts tumor growth and enhances immune response, particularly in PD-L1^+^ TNBC. One promising approach involves targeting the CD44v6 antigen, which is implicated in tumorigenesis, invasion, and metastasis. CD44v6 is a cell-surface glycoprotein involved in cell adhesion and proliferation. Researchers have developed a CD44v6 CAR vector through lentiviral transduction, introducing various functional domains, including IgG1, CD28, CD3ζ, a checkpoint inhibitor (PD1x), a suicide gene (HSV TK from herpes simplex virus type 1), and an IL-15 superagonist (15R15) under the control of an NFAT promoter. The efficacy of CD44v6 CARs was evaluated using 3D TNBC tumor spheroid models and Jurkat reporter cells, demonstrating significant tumor cell lysis, particularly in the presence of high PD-L1 expression, a common feature in BC cell lines [233]. Table 3 presents a comprehensive overview of monotherapy and combination approaches in CAR-T cell therapy across various BC subtypes, highlighting the diversity in targeted markers, cell lines, and animal models utilized in preclinical studies.

**Table 3:** Overview of CAR-T cell therapy characteristics for targeting BC

BC subtype	ICIs type	Intervention	Markers	Cell lines	Animal or human	Reference
TNBC	CD44v6-specific CAR	MCTS	Anti CD44v6	Jurkat, MCF7, HCC1937, MDA-MB-231, MDA-MB-468, HEK 293T, YT	-	[233]
TNBC	TF-CAR1	L-ICON	CD16	NK92MI, 293TN, 293AD, MDA-MB-231, 4T1, ADCC	4 to 6 weeks old female NSG	[234]
TNBC	ROR1-CAR-T cells	-	CD8, CD45, CD4, CD69, CD25	MDA-MB-231	-	[248]
TNBC	ROR1-CAR-T cells	SD-208	CD8^+^ and CD4^+^ T-cells	MDA-MB-231	-	[339]
TNBC	MUC28z CAR-T cells	-	CD25, CD11c, PD1	TNBC cell lines, GP2-293, AG11132, AG11134	7 to 8 weeks old female NSG mice	[235]
TNBC	CAR.MUC1.TR2.41BB T-cells	TR2.41BB receptor	CD14^+^, CD33^+^, CD11b^+^, CD4^+^, CD8^+^	PBMCs, 293T, MDA-MB-231, MDA-MB-453, SUM-159, BT-20	6 week to 8 weeks old female NSG	[236]
TNBC	EGFR CAR-T cell	THZ1	CDK7	HEK293T, MDA-MB-231, MDA-MB-468, EMT6	6 to 12 weeks old female nude mice	[242]
TNBC	EGFR CAR-T-cells	-	CD3, CD8, CD25, CD69, CD62L	MDA-MB‐231, MCF7, HS578T and SK‐BR‐3, BT474, MDA‐MB‐468, Hcc1806, T47D, MCF10A	6 to 12 weeks old female SCID mice	[243]
TNBC	EGFR-targeted CAR-T cells	radiotherapy	CD4^+^ T, CD8^+^ T	4T1, E0771, Icam1-KO	6 to 8 weeks old C57BL/6, Balb/c, and NPG mice	[245]
TNBC	EGFR-specific CAR-T cells	-	CD3, CD4, CD8	MDA-MB-231, MDA-MB-468, HS578T, MCF-7	female nude mice	[244]
TNBC	anti-MSLN CAR-T exosomes	-	CD4, CD8, CD3, MSLN, CD63, PRF1 and GZMB	BT-549, MDA-MB-231	6 to 8 weeks old B-NDG mice	[237]
TNBC	PD-L1-targeted shark VNAR-based single-domain CAR-T cell	-	anti-CD3, anti-CD28	MDA-MB-231	5 week old female NSG mice	[251]
TNBC	TEM8/ANTXR1-specific CAR-T cells	-	TEM8, CD44, CD24, monoclonal antibody L2	Hs578T, MDA-MB-231, MDA-MB-436, MDA-MB-468, SK-BR-3, HC6020	4 week old SCID/Bg mice	[250]
TNBC	ICAM1-specific CAR-T cells	-	CD3, CD4, CD45RO, CD62L, CD8	HEK-293T, A-431, Hela, MDA-MB-231, MDA-MB-468, SKBR3, MCF10a	6 week old female NSG mice	[239]
TNBC	NKG2D CAR-T cells	IL-2	CD45, CD4, and CD8 T	MCF7 and TNBC cell lines MDA-MB-231, MDA-MB-436, MDA-MB-468, MDA-MB-453, BT549, AE17, 293T	6 to 10 weeks old female NSG mice	[252]
TNBC	pan-ErbB T1E28z CAR-T cells	PET	CD3, CD4, CD8, CD155, CD112, B7-H3, B7-H4	MDA-MB-231, MDA-MB-436, MCF-7, HCC1954	5 to 6 weeks old female NSG mice	[256]
TNBC	AXL-CAR-T cells	IL-7	CD69, CD4, CD8	MDA-MB-231, MDA-MB-468, MCF-7	5 to 7 weeks old female NOD-SCID IL-2	[246]
TNBC	AXL-CAR-T cell	-	CD28, CD137, CD3, CD4	HT1080, MDA-MB-231, MCF-7, Panc1, MiapacaII, 786-O, 769-P	NSG mice	[247]
TNBC	SSEA-4-specific CAR-T cells	-	CD8^+^, ΔLNGFR	HEK293T, MCF-7, MDA-MB-231	8 to 10 weeks old female NOD/scid/IL2rγ-/- (NSG) mice	[240]
TNBC	FRα-CAR-T cells	-	CD3, CD4, CD8, CD107a	MCF-7, MDA-MB-231, MCF10A	-	[253]
TNBC	FRα-CAR-T cells	inhibitors of IL6-STAT3/AKT-PD-L1 axis	CD3, CD4, CD8, fibroblasts markers	MDA-MB-231, HCC70, CAFs, NFs	-	[255]
TNBC	Meso-CAR-T cells	rAd.sT	CD45, CD3, CD8, CD4	293T, HT1080, MIAPaCa-2, MDA-MB-231, MCF-7	6 to 8 weeks old B-NSG mice	[238]
LABC	LH28z CAR-T cells, Th/Tc17 CAR-T cells	STING	Anti-GR1 Anti-PD1 anti-CD28 mAb	3T3, NT2, PLAT-E	8 to 12-weeks old female FVB-Neu mice (FVB/N-Tg(MMTVneu)	[257]
MBC	mRNA c-MET-CAR-T cells	-	c-MET, CD3, CD4, CD8, CD68	BT20, MDA-MB-231, SK-OV-3, TB129	7–8 week-old male and female NSG mice; 4 of 6 patients had metastatic TNBC, and 2 had ER^+^HER2, MBC	[258]
MBC	CAR.MUC1 T-cells	4/7ICR and 2G CAR	CD3, CD8, Rat Anti-Mouse IgG1, anti-MUC1	PBMCs, MDA MB 468, MCF-7, 293T	6 to 8 weeks old female NSG mice	[259]
BC	HERV-K-specific CAR-T cells	Ras	CD8^+^, CD4^+^, CD3, CD25, FoxP3	MCF-7, SKBR3, MDA-MB-231, MDA-MB-435.eB1 HEK293, K562, MCF-10 A	8 week old female NOD/SCID mice	[260]
BCBMs	EGFR-CAR-NK-92 cells	Alone or in combination with HSV	mouse monoclonal anti-human EGFR	MDA-MB-231, MDA-MB-468, MCF-7, 293T, Phoenix cells, NK-92	6 to 8weeks old (NSG) mice	[265]
BCBMs	HER2-CAR-T cells	CD28 or 4-1BB	CD8, CD4, CD45RO, CD45RA, CD62L, CD95, CCR7	MDA-MB-361, MDA-MB-231, SKBR3, BT474, BBM1, LCL	female NSG mice	[266]
BRCA1/2 BC	806-28Z CAR	olaparib	CD45^+^, CD8	E0771, 4T1, 293T, 3T3	4 to 6 weeks old C57BL/6 and BALB/c mice	[262]
HER2^+^ BC	HER2-specific CAR-T cells	-	CD44	HEK 293T, N87, SK-BR-3, MDA-MB-468, CD16.176 V.NK-92	7 week old female NSG mouse	[263]

**Abbreviations list**: TNBC, triple-negative BC; MCTS, monocarboxylate transporters; CAR, chimeric antigen receptor; ADCC, antibody-dependenT-cell-mediated cytotoxicity; PBMCs, peripheral blood mononuclear cells; SCID, severe combined immunodeficient; NSG, NOD-SCID IL-2rγ-/-; FRα, folate receptor α; ICAM1, intercellular adhesion molecule 1; ER^+^, estrogen receptor-positive; HER2^−^, human epidermal growth factor receptor 2 negative; HERV-K, human endogenous retrovirus K; MBC, metastatic BC; BCBMs, BC brain metastases; EGFR, epidermal growth factor receptor; CAR-NK, chimeric antigen receptor natural killer; MDA-MB-231, human BC cell line; MDA-MB-468, human BC cell line; MCF-7, human BC cell line; BT-549, human BC cell line; SUM-159, human BC cell line; MDA-MB-453, human BC cell line; HEK 293T, human embryonic kidney cell line; IL-2, interleukin-2; IL-7, interleukin-7; IL-6, interleukin-6; STAT3, signal transducer and activator of transcription 3; AKT, protein kinase B; NPG, NOD-Prkdcγ-/- IL-2rγ-/-; 293T, human embryonic kidney cell line; 4T1, mouse mammary carcinoma cell line; T47D, human BC cell line; HCC70, human BC cell line; NOD, non-obese diabetic; FVB, Friend virus B; B7-H3, cluster of differentiation 276; B7-H4, cluster of differentiation 273; C57BL/6, mouse strain; BALB/c, mouse strain; CD137, cluster of differentiation 137; Hs578T, human BC cell line; TEM8, tumor endothelial marker 8; ANTXR1, anthrax toxin receptor 1; Ras, rat sarcoma

Tissue factor (TF), coagulation factor III, has been identified as an on-target molecule in TNBC. L-ICON, a second-generation immunoconjugate that targets TF, has shown enhanced efficacy against TNBC cells in antibody immunotherapy; however, its effectiveness is compromised by NK cells. Researchers developed TF-targeting CARs on NK cells to address this challenge and assessed their cytotoxicity against TNBC cells. In vivo studies demonstrated encouraging results, including tumor growth inhibition and reduced tumor weight in mouse models [234]. Additionally, mucin 1 (MUC1), a transmembrane mucin protein, is overexpressed and aberrantly glycosylated in over 90% of BC cases, including TNBC. A second-generation CAR specific to human tMUC1 (MUC28z) was engineered using the scFv sequence, fused with CD28 and CD3ζ signaling domains. The antitumor efficacy of MUC28z CAR-T cells has been validated both in vitro and in vivo, showing increased expression of CD25, CD11c, and PD-1, along with decreased levels of CXCR4 and CD62L, significant tumor growth reduction, and enhanced production of IFN-γ and GZMB [235].

MDSCs are also prevalent in the BC TME and contribute to immune suppression and metastasis. Targeting MDSCs using a TR2 agonistic antibody (DS-8273a) has been shown to reduce MDSC numbers in both peripheral blood and tumor sites. Leveraging this, researchers developed CAR. MUC1 T-cells with a costimulatory receptor (TR2.41BB) to enhance antitumor activity in TNBC. In vivo, studies demonstrated significant tumor clearance and inhibition of metastasis when these CAR-T cells were administered to mice bearing MDA-MB-231 tumors with MDSCs. To further assess the benefits of TR2.4-1BB expression in CAR-T cells, the study tested a combination of TR2.4-1BB and CAR. HER2 targeting MDA-MB-453 cells in the presence of MDSCs. This combination exhibited strong antitumor effects, suggesting that the TR2.4-1BB receptor can augment CAR-T cell responses against both MUC1 and HER2 antigens, effectively eliminating MDSCs and leading to improved T-cell survival, proliferation, and persistence at the tumor site [236]. Recent developments also include engineering CAR-T cells to target mesothelin, a protein overexpressed in specific TNBC subtypes. Exosomes derived from these CAR-T cells carrying PRF1 and GZMB exhibited high cytotoxicity against TNBC cell lines and reduced tumor volumes in xenograft models, highlighting the potential of exosome-mediated therapy in TNBC [237].

Mesothelin, a glycosylphosphatidylinositol (GPI)-linked protein, is usually expressed on mesothelial cells but is overexpressed in many cancers, promoting tumor growth, survival, and metastasis. Given its role in cancer progression, mesothelin has become a key target for CAR-T cell therapy. Lentiviral plasmids expressing meso-CAR were used to engineer T-cells, which were then tested against TNBC cells. These meso-CAR-T cells demonstrated enhanced cytotoxicity and elevated production of cytokines and effector molecules such as IL-2, IL-6, and IFN-γ. In an MDA-MB-231 xenograft mouse model, meso-CAR-T cells effectively inhibited tumor growth, reduced tumor weight, and prevented liver metastasis. Additionally, combining meso-CAR-T cells with the oncolytic adenovirus rAd.sT (a soluble TGFβ receptor) showed superior anti-tumor effects, with increased cytotoxicity and enhanced apoptosis of tumor cells in vivo [238].

TNBC cells also exhibit high levels of ICAM1, a transmembrane glycoprotein receptor associated with metastasis and poor prognosis. ICAM1-specific CAR-T cells were engineered to target this using an mG2-scFv scFv, Myc-tag, CD28, 4-1BB, and CD3ζ intracellular domains. In vitro, these ICAM1-CAR-T cells killed over 50% of MDA-MB-231 and MDA-MB-468 cells and 20% of MCF-7 cells, with elevated IFN-γ and IL-2 levels compared to control CAR-T cells. In vivo, they effectively reduced tumor size in MDA-MB-231 xenograft models, accompanied by increased cytokine levels (IL-2, IL-6, TNF-α, IFN-γ) and many CD3^+^ cells. At the same time, histological analysis showed decreased ICAM1 expression in treated tumors [239].

SSEA-4 is associated with a chemoresistant and highly metastatic subpopulation of TNBC. To target SSEA-4, second-generation CARs with different spacer regions, including the IgG4 hinge-CH2CH3 (L spacer), CD8α hinge (S spacer), and IgG4 hinge (XS spacer) CAR constructs, were developed. In vitro tests with MCF-7 and MDA-MB-231 cell lines showed varied SSEA-4 expression levels, with XS spacer CAR activating about 20% of CD8^+^ T-cells, compared to over 40% and 50% activation by the L and S spacer CARs, respectively. Cytokine secretion was higher for the L and S spacer CARs, with the S spacer CAR being the most reactive. However, in vivo studies revealed a weak antitumor response and adverse effects, particularly with the S spacer CAR, which led to decreased body weight and increased levels of human IL-2 and TNFα in the blood [240].

Nevertheless, CAR-T cell therapy can increase the likelihood of T-cell malignancies if retroviral or lentiviral vectors integrate close to tumorigenic regions within the genome. Persistent T-cell activation and an intensified inflammatory milieu following CAR-T infusion may further spur the emergence of malignant clones. In addition, many CAR-T recipients are immunocompromised, rendering them more susceptible to secondary cancers. Pre-infusion interventions, such as bridging therapies and lymphodepletion, also heighten this risk by introducing DNA mutations. Because of these concerns, vigilant and ongoing monitoring for potential or newly characterized adverse effects is vital to predict, mitigate, or avert unintended outcomes. Early detection and appropriate intervention are key to preserving the safety profile of CAR-T therapy and ensuring its continued advancement in cancer treatment [241] Table 3.

### Tyrosine kinase receptor targets in CAR-T cell therapy for BC

Tyrosine kinase receptors, such as EGFR, are commonly overexpressed in TNBC cells. These receptors act as signals for cell growth and survival, which makes them ideal targets for CAR-T cell therapy. By designing CAR-T cells to recognize and bind to EGFR on cancer cells, researchers can help convert cold tumors, which generally lack immune cell infiltration, into hot tumors that attract immune activity. The result is an environment more conducive to an immune response, potentially enhancing the efficacy of immunotherapies against BC.

Combining EGFR CAR-T cells with CDK7 inhibitors like THZ1 improved therapeutic outcomes by attenuating the expression of immunosuppressive genes induced by IFN-γ signaling [242]. In addition, engineering EGFR-targeted CAR-T cells for TNBC using an Fc-EGFR CAR lentivirus showed promising results. These CAR-T cells, when incubated with TNBC cells (MDA‐MB‐231 or MDA‐MB‐468), demonstrated significant activation, evidenced by increased CD69^+^/CD8^+^ and CD25^+^/CD8^+^ subpopulations and elevated cytokine production (TNFα, IL‐2, IFN-γ). They showed enhanced tumor cell-killing activity in a dose- and time-dependent manner while activating multiple signaling pathways, including granzyme–PRF1–PARP, Fas–FADD–caspase, and IFN-γ pathways. Gene expression analysis revealed up-regulation of genes associated with naïve T-cells and down-regulation of effector T-cell-related genes, highlighting the therapeutic potential of EGFR-targeted CAR-T cells, which was further confirmed in the TNBC xenograft mouse model [243].

Furthermore, a study comparing EGFR expression levels revealed that TNBC cell lines (HS-578T, MDA-MB-468, MDA-MB-231) exhibited 3 to 17 times higher EGFR RNA and protein levels compared to the non-TNBC MCF-7 cell line. Two types of EGFR-specific CAR-T cells (EGFR-CAR-1 and EGFR-CAR-2) were developed, effectively inducing TNBC cell lysis in vitro. This was accompanied by a significant increase in the secretion of Tc1 (IFN-γ, IL-2) and Tc2 (IL-4) cytokines, thereby enhancing cytotoxicity. In patient-derived xenograft (PDX) mouse models, treatment with EGFR-specific CAR-T cells inhibited tumor growth without affecting body weight, demonstrating their potential efficacy against TNBC (Table 3) [244].

Additionally, a study combining CAR-T cells with radiotherapy to enhance anti-tumor efficacy against TNBC revealed that irradiating TNBC cells before co-culturing with EGFR-targeted CAR-T cells increased cytotoxicity. Although there was no significant difference in tumor growth inhibition or body weight between CAR-T cell infusions at 4 h versus 48 h post-irradiation, the 4-hour infusion led to prolonged survival in mice. RNA-seq analysis showed increased CD8^+^ T-cells and NK cells in tumors treated with CAR-T cells and radiation, with upregulation of leukocyte transendothelial migration genes, particularly Icam1. Additionally, irradiation activated the Icam1 promoter and NF-κB signaling, further contributing to the therapeutic effect [245]. AXL, an overexpressed RTK in BC, is linked to poor survival outcomes. AXL-targeting CAR-T cells, especially when coexpressed with C7R, have demonstrated antitumor solid activity against AXL^+^ TNBC cells, such as MDA-MB-231 and MDA-MB-468. These CAR-T cells showed increased cytotoxicity, cytokine production (IL-2, IL-4, IL-6, IFNγ, and TNF-α), CD69 expression, and proliferation. Both AXL-CAR.C7R-T and AXL-CAR-T cells effectively reduced tumor size in MDA-MB-231 xenografts in animal models. Immunohistochemical analysis revealed that AXL-CAR.C7R T-cells had enhanced persistence and antitumor activity, marked by higher CD3 and GZMB levels in treated tumors [246].

In addition, the growth arrest-specific protein 6 (GAS6) interacts with AXL, leading to AXL dimerization, autophosphorylation, and activation of downstream signaling pathways. The AXL/GAS6 axis is highly expressed in tumor cell lines and BC tissues and contributes to critical processes such as proliferation, apoptosis, survival, migration, inflammation, and angiogenesis. In experiments, co-culturing MDA-MB-231 cells with AXL-CAR-T cells resulted in increased cytokine levels and significant tumor growth inhibition in xenograft models. The presence of human CD3^+^ T-cells exclusively in the tumor tissues of the AXL-CAR-T cell group indicates effective targeting and anti-tumor activity [247].

The further study explored the antitumor effects of ROR1-CAR-T cells in micro physiologic 3D tumor models of TNBC, specifically the MDA-MB-231 cell line. This study revealed a significant increase in tumor mass in dynamic culture conditions and an initial spike in apoptosis induced by ROR1-CAR-T cells within the first 24 h of treatment, followed by a reduction in subsequent days. High levels of IFN-γ, IL-2, CD25, CD69, CD45^+^, and PD-1 were expressed, indicating robust antitumor activity [248]. Another study investigated the impact of TGF-β on ROR1-CAR-T cells, given that TGF-β is a typical immunosuppressive cytokine in the TNBC microenvironment. The binding of TGF-β to T-cells activates SMADs, leading to apoptosis and reduced CAR-T cell efficacy. **T**he study proposed treating ROR1-CAR-T cells with SD-208, a TGF-β receptor signaling blocker. This approach preserved the antitumor function of ROR1-CAR-T cells in vitro and within 3D tumor models [249].

Besides RTKs, targeting tumor endothelial marker 8 (TEM8), also known as anthrax toxin receptor 1 (ANTXR1), with specific CAR-T cells has also been explored as a therapeutic strategy for TNBC. Second and third-generation TEM8-specific CAR-T cells were developed, with the latter showing superior antitumor activity and survival benefits in preclinical models. These findings underscore the potential of TEM8 as a target for TNBC therapy [250].

### CAR-T cell targeting immune-related proteins in BC

Innovative approaches using single domain shark variable new antigen receptor (VNARs) antibodies have also shown promise in targeting PD-L1. CARs incorporating VNAR B2, which blocks the interaction between PD-L1 and PD-1, demonstrated effective lysis of PD-L1^+^ TNBC cells in vitro and tumor regression in xenograft models. This method holds potential for broader applications across different cancer types [251]. NKG2D ligands, commonly expressed on primary tumor cells and immunosuppressive cells within the TME, have been targeted using NKG2D-specific CAR-T cells. Researchers developed three CAR-T cell generations: first-generation GFP-NKG2D-z, second-generation GFP-NKG2D-BBz, and third-generation GFP-NKG2D-27z. When stimulated with NKG2DL^+^TNBC cells, the second and third generations secreted high levels of IFN-γ and exhibited enhanced cytotoxicity against TNBC cell lines, including BT549, MDA-MB-436, and MDA-MB-438. In vivo studies further confirmed that NKG2D-BBz and NKG2D-27z CAR-T cells effectively inhibited tumor growth and demonstrated significant antitumor activity, making them promising candidates for TNBC treatment [252].

Furthermore, folate receptor α (FRα) is overexpressed in several malignancies, including TNBC, making it an attractive target for CAR-T cell therapy. FRα expression in BC can be modulated by steroid hormones, particularly estrogens, with a negative correlation observed between ER and FRα expression. Specifically, ER-negative BC samples are more likely to express FRα. A FRα-specific CAR-encoding lentivirus, comprising the anti-human FRα-specific MOv19 scFv, was constructed and tested. Co-culture of FRα CAR-bearing T-cells with FRα^+^TNBC cell lines led to Th1 cytokine secretion and upregulation of CD137 (4-1BB). FRα CAR-T cells demonstrated robust and specific cytotoxic activity against FRα^+^MDA-MB-231 cells. In vivo studies showed that mice treated with FRα-specific CAR-T cells had higher levels of human CD4^+^ and CD8^+^ T-cells in the blood, reduced tumor growth, and stable FRα expression [253]. Additionally, MDA-MB-231 cells engineered to overexpress FRα exhibited significantly enhanced CAR-T cell-mediated lysis, reducing tumor volume and lowering residual tumor burden in mice [254]. A subsequent study confirmed the potential of FRα-CAR-T cell therapy against BC. Co-culture of FRα-CAR-T cells with BC cells led to CD107a upregulation, indicating T-cell activation. The cytotoxicity of FRα-CAR-T cells was assessed in both 2D cell cultures, where they demonstrated significant T-cell lysis, and in 3D spheroid models, where they reduced spheroid size and caused structural disruption [253].

Furthermore, CAFs play a significant role in the TME, mainly through the secretion of cytokines like IL-6, which can influence immune cell function. The study examined the impact of CAF-derived IL-6 and PD-L1 on FRα-CAR-T cell efficacy in TNBC cell lines. Conditioned media from CAFs had significantly higher IL-6 levels than media from normal fibroblasts, with most CAF samples showing elevated IL-6. High IL-6 levels in CAF media induced PD-L1 expression in TNBC cells and contributed to doxorubicin-induced apoptosis. Elevated IL-6 activated PD-L1 expression through the pSTAT3 and pAKT signaling pathways. When FRα-CAR-T cells were co-cultured with FRα-expressing cancer cells in 2D and 3D cultures, CAF-derived IL-6 reduced CAR-T cell cytotoxicity by increasing PD-L1 levels [255].

The application of CAR-T cells in clinical trials has encountered limitations, including challenges with tumor infiltration and severe side effects. To improve CAR-T cell therapy, researchers have suggested using whole-body in vivo imaging, particularly positron emission tomography (PET), for tracking CAR-T cell activity. A sensitive, non-invasive PET tracking platform was applied to T1E28z-CAR-Targeting the ErbB receptor family in TNBC xenograft models. Three CAR-T cell variants were tested: T4NT (functional with a reporter), T4ΔNT (non-functional), and T4 (functional without a reporter). The radiotracers [99mTc]TcO4 – and [18 F]BF4 – showed no significant impact on the cytotoxicity, uptake, or viability of T4NT CAR-T cells. While T4NT CAR-T cells showed higher retention and stronger signals in MDA-MB-436 tumors, they did not affect tumor size in MDA-MB-231 models. Additionally, higher levels of immune checkpoint and B7-Class inhibitory molecules (PD-L1, CD112, B7H4) were found in MDA-MB-231 cells, with lower CD155 expression compared to MDA-MB-436 cells [256].

### CAR-T cell therapy in different BC subtypes

This section discusses the detailed therapeutic approaches and outcomes based on running trials for CAR-T cell therapy across different BC subtypes. The strategies and mechanisms of each subtype-TNBC, HER2^+^ BC, BRCA1/2 mutant BC, and brain metastatic BC differ according to their unique TME and immunological profiles. TNBC is often considered a cold tumor. However, clinical trials targeting specific antigens like EGFR or CD44v6 have shown promise in improving immune cell recognition, transitioning the tumor into a hot immune environment. In HER2^+^ BC, trials targeting HER2 with CAR-T cells have demonstrated effectiveness, especially when combined with ICIs, enhancing T-cell activity. Trials for BRCA1/2 mutant BC explore how defects in DNA repair and higher mutation rates might increase the immunogenicity of these tumors, offering new avenues for CAR-T cell therapy. Finally, trials in brain metastasis BC focus on overcoming the blood-brain barrier, with localized CAR-T cell delivery or engineered T-cells designed to cross the barrier, potentially enabling the cold-to-hot transition in these uniquely challenging tumors. Each approach considers the specific immunophenotypic features of the cancer subtype, making tailored CAR-T cell therapies a promising frontier in personalized treatment for BC.

Figure 4 provides a comprehensive view of how CAR-T cell therapy can target BC by focusing on specific tumor antigens like FRα, MUC1, and EpCAM, often overexpressed in BC cells. This activation leads to T-cell proliferation and the release of cytotoxic granules, resulting in targeted tumor cell destruction. Additionally, the figure emphasizes the challenges CAR-T cells face in the immunosuppressive TME—a Cold tumor. To overcome these barriers, the figure outlines emerging strategies to enhance CAR-T cell functionality, such as incorporating co-stimulatory domains that amplify T-cell activation and the use of combination therapies to modulate the microenvironment.

### Locally advanced BC

In a recent study, second-generation murine CAR constructs targeting the proto-oncogene Neu were developed, utilizing costimulatory domains from CD28 (LH28z) or CD137. CAR-T cells have successfully treated patients with solid tumors, notably through the migration and persistence of T helper 17 (Th17) and CD8^+^ T 17 (Tc17) cells, which release IL-17 A. These cells exhibit antitumor effects by increasing levels of CD4^+^ and CD8^+^ in TME and enhancing short-term survival in vivo. However, for long-term results, the study demonstrated that the administration of the STING agonist DMXAA in combination with anti-PD-1 and anti-GR-1 mAbs therapy significantly improves persistence, OS, cytotoxicity, and antitumor activity of Th/Tc17 CAR-T cells within the TME [257].

### Metastatic BC (MBC)

The HGF receptor, c-MET, is commonly expressed in MBC, leading to the hypothesis that c-MET-targeted CAR-T cells could be an effective therapy for this disease. Initially, c-MET-CAR-T cells effectively killed tumor cells in TNBC and HER2^+^BC lines in vitro, significantly controlling tumor growth in vivo. Furthermore, an open-label phase 0 clinical trial involving six patients with metastatic BC showed promising results, with c-MET-CAR-T cell therapy leading to tumor necrosis, hemorrhage, and significant infiltration of T-cells (CD3⁺, CD4⁺, and CD8⁺) and macrophages (CD68⁺) at the injection sites. The trial also monitored pharmacokinetics, AEs, dose-limiting toxicity (DLT), and maximum tolerated dose (MTD), affirming the safety and feasibility of this approach. These findings collectively suggest that c-MET-CAR-T cells hold significant potential as a targeted treatment for MBC, with demonstrated efficacy in preclinical and early-phase clinical settings (NCT01837602) [258].

Researchers engineered a first-generation human CAR (1G) explicitly targeting and killing MUC1-expressing tumors further to investigate CAR-T cell therapies for metastatic BC. Acknowledging the immunosuppressive TME, they also developed an inverted cytokine receptor (ICR) that combines the exodomain of the IL-4 receptor with the IL-7 receptor (designated as 4/7ICR). While the 1G.4/7ICR T-cells exhibited superior cytolytic capacity in the presence of recombinant IL-4, they did not demonstrate enhanced anti-tumor activity against MDA-MB-468 BC cells. A second-generation CAR-T cell (2G.4/7ICR) was constructed to overcome this limitation, which maintained effective tumor cell killing even after prolonged exposure. Notably, in vivo experiments showed that all mice treated with 2G.4/7ICR T-cells remained tumor-free, indicating durable anti-tumor activity [259]. In another study, researchers identified the HERV-K envelope protein as a promising target for CAR-T cell therapy in BC. HERV-K, a family of human endogenous retroviruses, expresses viral proteins in malignant cells, triggering immune responses. A HERV-K-specific CAR (K-CAR) was developed using a single-chain monoclonal antibody (6H5). In vitro, K-CAR^+^ T-cells from BC patients inhibited tumor growth and specifically lysed cancer cells, particularly in MDA-MB-231. The release of IFN-γ, TNF-α, and GZMB further enhanced tumor cell killing. In vivo, K-CAR-T cells significantly reduced mouse tumor weight and size, downregulated HERV-K expression, upregulated p53, and reduced metastases. Immunohistochemistry confirmed the absence of HERV-K and H-Ras in treated tumors and metastatic cells [260].

A Phase II open-label, multicenter basket trial investigated the combination of ipilimumab and nivolumab in 17 patients with unresectable or metastatic metaplastic BC (MpBC), with a median age of 60. The primary endpoint was the ORR, encompassing confirmed complete responses (CR) and partial responses (PR), while secondary endpoints included toxicity, OS, and PFS. The trial results indicated a 6% CR rate and an 18% ORR, with durable responses observed at 28^+^, 33^+^, and 34^+^ months. However, AEs were significant, with 65% of patients experiencing AEs, 18% encountering grade 3–4 AEs, and one patient suffering a grade 5 AE. Notably, 47% of participants reported immune-related AEs (irAEs), such as liver function test abnormalities, adrenal insufficiency, and rash. The median PFS and OS were 2 and 12 months, respectively (NCT02834013) [261].

In conclusion, developing c-MET-targeted CAR-T cells represents a promising therapeutic avenue for MBC, particularly for aggressive subtypes such as TNBC and HER2^+^ BCs. Preclinical and early-phase clinical trials have demonstrated the potential of c-MET-CAR-T cells to effectively target and kill tumor cells, with encouraging signs of tumor control and immune response at treatment sites. This innovative approach underscores the feasibility and safety of c-MET-directed immunotherapy. Further advancements in CAR-T cell technology, including the engineering of CARs targeting MUC1 and the incorporation of inverted cytokine receptors, have shown sustained efficacy in preclinical models, paving the way for more robust and durable cancer treatments. Additionally, targeting the HERV-K envelope protein presents another promising strategy, with preclinical results indicating effective tumor inhibition and immune activation. Despite these advancements, challenges remain, particularly with combination therapies such as ipilimumab and nivolumab, which, while showing some efficacy, are associated with significant AEs. These findings highlight the need for continued research to optimize the safety and effectiveness of CAR-T cell therapies and other immunotherapeutic strategies, ultimately aiming to improve outcomes for patients with MBC.

### BRCA1/2 mutated BC

Olaparib, a potent PARP1 inhibitor, disrupts DNA repair in tumor cells and has been shown to induce CD8^+^ T-cell infiltration and activation and inhibit angiogenesis in BC. Veliparib, another PARP inhibitor, also shows potential, especially with chemotherapy. EGFRvIII, a common mutant form of EGFR, is implicated in cancer metastasis, including BC. This study combined olaparib with EGFRvIII-targeting CAR-T cells (806-28Z CAR) in a murine model. The results showed that olaparib enhanced the cytotoxicity, proliferation suppression, and apoptosis induction of CAR-T cells, improving their persistence and antitumor activity. Olaparib also increased the presence of CD45^+^ immune cells and reduced MDSCs in tumors. Additionally, olaparib treatment downregulated CXCR4 in EGFRvIII^+^ tumor cells and SDF1α in CAFs, reducing MDSC migration and potentially improving therapeutic outcomes [262].

### HER2^+^ BC

Trastuzumab (Herceptin^®^) is an FDA-approved monoclonal antibody for HER2^+^BC that improves progression-free and OS, especially when combined with other drugs. However, resistance to trastuzumab is common, prompting exploration of HER2-specific CAR-T cells. The CAR-T cells, engineered with a trastuzumab-derived scFv, showed enhanced activation and IFN-γ release when co-cultured with HER2^+^ targe cells (MDA-HER2 or JIMT-1) or CD16.176 V.NK-92 cells, indicating T-cell activation. In 3D cultures, HER2-CAR-T cells exhibited significant cytolytic activity and penetrated the spheroid core, while trastuzumab-bound CD16.176 V.NK-92 cells did not. In vivo, treatment with HER2-CAR-T cells in NSG mice led to complete and long-lasting tumor regression in HER2^+^ MDA-HER2.ffLUC and JIMT-1 models [263].

In addition, resistance mechanisms like MUC4 and the CD44/Hyaluronan complex impact trastuzumab efficacy. To address this, HER2-CAR-T cells were constructed with a trastuzumab-derived scFv, an IgG1 CH2-CH3 stalk, a CD28 costimulatory domain, and a CD3ζ effector domain. In vitro, these CAR-T cells showed increased IFN-γ secretion and cytotoxicity against JIMT-1 cells, a trastuzumab-resistant line. In vivo, a low dose of HER2-CAR-T cells improved OS, complete tumor regression, and long-term tumor-free survival in mice [264].

### BC brain metastatic (BCBMs)

Given the poor prognosis for patients with BC Brain Metastatic (BCBMs), innovative therapies are urgently needed. One study engineered NK-92 and primary NK cells to express a second-generation EGFR-CAR, targeting the highly expressed EGFR in BCBMs. When co-cultured with BC cell lines, these EGFR-CAR-NK cells showed increased IFN-γ secretion and cytotoxicity. Additionally, oncolytic herpes simplex virus type 1 (HSV-1) was identified as a promising vector for cancer gene therapy, capable of activating NK cells, limiting viral replication, and improving BCBM treatment. The combination of EGFR-CAR-NK-92 cells and HSV-1 in vitro led to rapid lysis of BC cells and, in vivo, reduced tumor growth and extended survival to 80 days in a mouse model [265].

Another preclinical study targeted brain metastasis through intraventricular delivery of HER2-CAR-T cells using CD28-containing (HER2-28ζ) and 4-1BB-containing (HER2-BBζ) designs. Both HER2-CAR-T cells showed robust activity against BBM1 cells, with HER2–28ζ T-cells exhibiting higher IFN-γ and CD107a expression and more excellent exhaustion markers than HER2-BBζ T-cells. In vivo, HER2-BBζ CAR-T cells demonstrated better proliferation, leading to complete tumor regression and extended survival in mice with BBM1 cells. Intraventricular delivery of HER2-BBζ CAR-T cells also showed promise in eradicating multifocal and leptomeningeal HER2^+^ central nervous system (CNS) disease [266].

In a separate trial utilizing a Simon two-stage design, 26 patients with BC brain metastases were treated with tremelimumab in combination with CNS RT, with or without trastuzumab. The cohort, with a median age of 50, included 20 HER2^−^ and 6 HER2^+^ patients. The primary endpoint focused on the non-CNS DCR, while secondary endpoints assessed non-CNS response rates, toxicity, PFS, and OS. The study reported a non-CNS DCR at week 12 of 10% in HER2^−^ and 17% in HER2^+^patients. CNS response rates were more promising, with a 15% response rate in the HER2^−^ group and 33% in the HER2^+^ group at week 12. Median OS was 4.9 months for HER2^−^ and 8.0 months for HER2^+^cohorts, with median PFS of 3.0 and 3.1 months, respectively. Treatment-related AEs were prevalent, affecting 79% of patients, with fatigue and nausea being the most common. HER2^+^ patients experienced a higher incidence of AEs, including one grade 3 AE, but no severe AEs (NCT02563925) [267].

In conclusion, the prognosis for patients with BC brain metastatic (BCBMs) remains dire, necessitating innovative therapeutic approaches. Promising advancements include the engineering of EGFR-CAR-NK cells and the use of oncolytic herpes simplex virus (HSV-1), which have shown enhanced cytotoxicity and extended survival in preclinical models. Additionally, HER2-CAR-T cells delivered intraventricularly have demonstrated robust anti-tumor activity and prolonged survival, particularly with the HER2-BBζ design. Clinical trials, such as the combination of tremelimumab, CNS radiotherapy, and trastuzumab, have also shown potential, especially in HER2^+^ patients, despite significant treatment-related AEs. Continued research is crucial to optimize these therapies and improve patient outcomes.

### Microbiome-based therapy in cold-to-hot tumor transition of BC

**Fig. 5:**
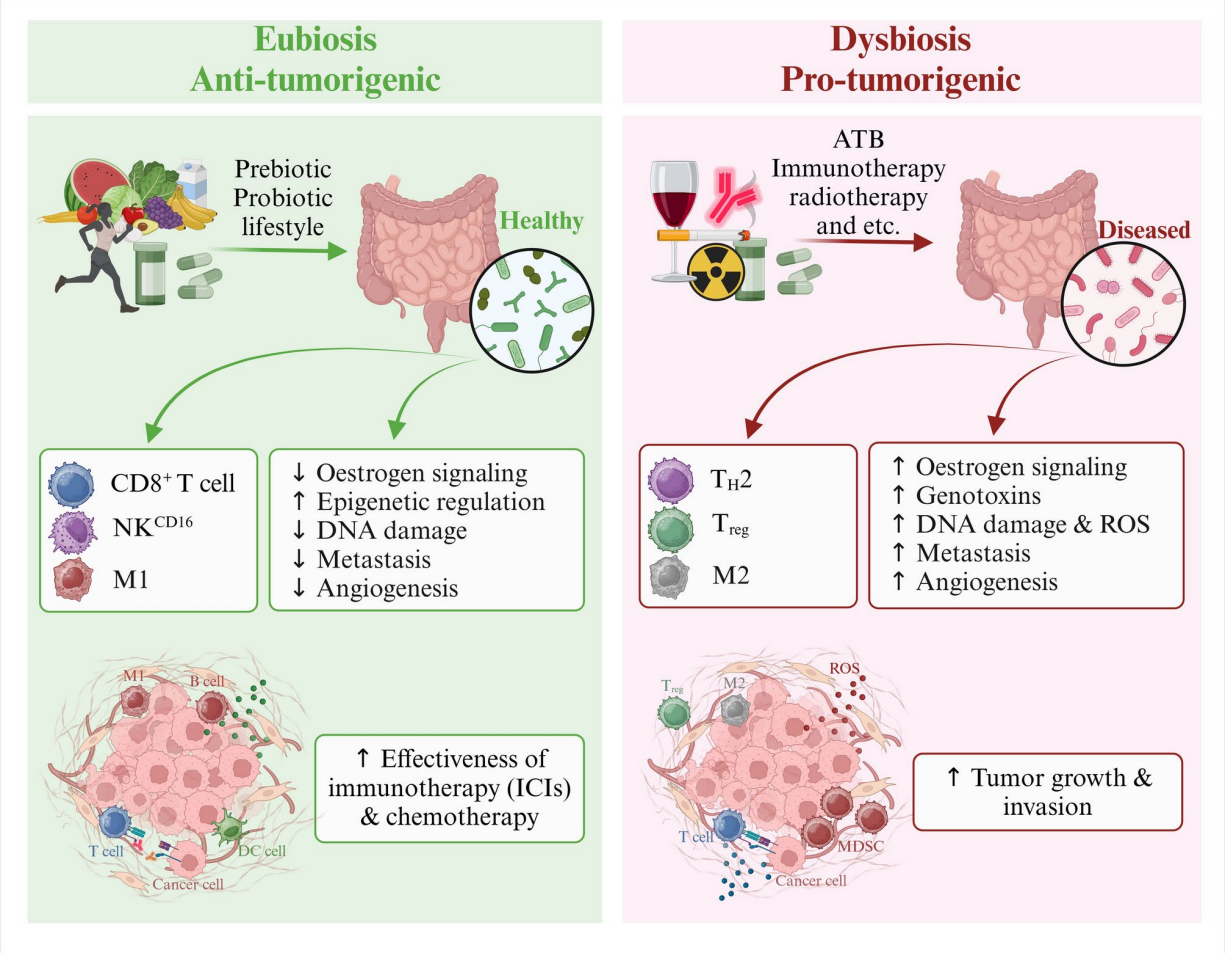
Impact of Microbiome on TME. This figure visually represents the multifaceted influence of the microbiome on the TME in BC. It illustrates how gut microbiota regulates systemic inflammation and estrogen metabolism, which can significantly impact BC progression. The figure highlights the interaction between gut microbiota and the immune system, showing how specific microbial populations can enhance or suppress immune responses, influencing tumor behavior. Furthermore, it depicts the epigenetic regulation of miRNAs by the microbiota, which can alter immune cell function and tumor dynamics. In addition, the role of microbial metabolism in bile acid synthesis and its effects on inflammation and immune modulation within the TME are illustrated. Annotations emphasize emerging research on the gut-tumor axis, underscoring the implications of microbiome composition for immune modulation and the development of therapeutic strategies in BC. This comprehensive overview will assist designers in effectively conveying the intricate relationship between microbiota and tumor immunity. *Created in BioRender. Jabbarzadeh Kaboli*,* P. (2024)*https://BioRender.com/v59d625

The microbiome, a complex community of microorganisms within the body, plays a key role in immune modulation and overall health [268]. In BC, an imbalanced microbiome (dysbiosis) can significantly impact the TME, often contributing to a cold tumor state with low immune cell infiltration and immune evasion. BC patients commonly show alterations in their gut microbiome, which influence tumorigenesis and estrogen metabolism. Dysbiosis in the TME impairs immune function, maintaining a cold TME (Fig. 5) [269].

Emerging evidence suggests that modifying the microbiome may help transition cold-to-hot ones. Restoring a balanced microbiome or targeting specific microbial signatures can enhance immune cell infiltration, increase tumor antigenicity, and “Heat” the TME. This shift may improve the efficacy of CAR-T cell therapy and ICIs, overcoming the immunosuppressive tumor environment and enhancing treatment responses in BC. Studies have shown that the gut microbiome in BC patients differs significantly from that of healthy individuals. This altered microbiome can also impact estrogen metabolism, a key factor in the pathogenesis of BC. Alterations in bacterial composition can also impair immune function, potentially leading to tumor development. Emerging evidence suggests that polymorphic microbiomes may serve as novel cancer markers, detectable through techniques such as sequencing and metagenomics [270].

Evidence indicates that the gut microbiome may also influence the effectiveness of chemotherapy by regulating drug translocation, metabolism, and immune response. For example, certain Bacteroides species, specifically *B. thetaiotaomicron* and *B. fragilis*, have been shown to enhance the therapeutic effect of anti-CTLA-4 antibodies. During immunotherapy, patients may experience increased levels of T-cells within the tumor environment, which can contribute to a more robust anticancer response [268].

Conversely, the microbiome can promote cancer development or mimic its presence by impairing metabolism and initiating inflammatory conditions. The composition of gut microbiota has been linked to an individual’s susceptibility to BC, with alterations in estrogen modulation and inflammatory responses within the TME being key factors. Reducing microbial diversity and losing keystone species can further contribute to carcinogenesis and immune dysregulation. For example, *Propionibacterium* has been found to activate oncogenic growth factors and T-cell-related genes, thereby reducing antitumor responses [271].

The gut and tumor microbiome are critical in immunological responses to BC development. The tumor cells and local microorganisms can activate pro-inflammatory and anti-inflammatory mediators, promoting tumor growth, cancer cell invasion, and metastasis. As inflammation and angiogenesis increase, the abundance of microorganisms in the TME also rises, further facilitating tumor progression [272]. In addition, microbes can also alter the pharmacodynamics of anticancer treatments and affect crucial cancer hallmarks, including tumor development and progression. Poor microbiome health can also impair estrogen production, leading to additional inflammation [272].

Moreover, microbiome metabolites are another crucial element that can significantly influence the modification of the TME. These metabolites can impact critical processes by impacting inflammation, proliferation, and cell death [273]. A reduction in *Faecalibacterium* levels leads to increased phosphocholine synthesis. *Faecalibacterium* also regulates cancer cell proliferation and invasion by inhibiting the IL-6/STAT3 pathway. Lithocholic acid, a significant metabolite, can influence cell proliferation by activating the Takeda G-protein-coupled receptor 5 (TGR5) [273].

Additionally, the composition of the gut microbiome differs between postmenopausal and premenopausal women, affecting metabolite production [274]. The gut microbiota plays a critical role in shaping immune responses, directly impacting the transformation of tumors from cold to hot states in BC. Changes in microbial composition can reduce the differentiation of pathogenic T-cells, mitigate inflammatory diseases, or increase Tregs cells, enhancing immune tolerance. This section explores how microbiome-based therapies can reshape the TME in different BC subtypes. By shifting the TME from cold to hot, these approaches can optimize therapeutic strategies and improve outcomes.

### Gut microbiota in Estrogen level regulation of tumor transformation

Dysregulation of steroid hormones, particularly estrogen, is a key factor in BC development. Studies have shown that gut bacteria significantly impact hormone metabolism and circulation. This influence may contribute to the transformation of tumors from cold to hot ones. Within the gut, microbial enzymes, particularly β-glucuronidase, facilitate the deconjugation of estrogen, allowing its reabsorption as free estrogen, which then circulates to various tissues, including the BC.

In cases of gut dysbiosis, increased β-glucuronidase activity can elevate estrogen reabsorption, leading to higher circulating estrogen levels in BC patients compared to healthy individuals. This elevation of estrogen may promote tumor growth and foster a pro-inflammatory hot TME by stimulating immune cell infiltration and altering local cytokine levels. Moreover, the interplay between BC’s dysregulated steroid hormones and bone metastasis further underscores gut microbiota’s role in shaping tumors’ immune landscape and metastatic potential [269]. These dynamics highlight the significant impact of gut microbiota on disease progression and therapeutic responses. There is also evidence that shows a strong relationship between steroid hormones and bone metastasis in BC [275].

Figure 5 illustrates the microbiome’s impact on the TME, particularly in BC, highlighting its potential role in transforming cold-to-hot tumors. The figure portrays how gut microbiota influence systemic inflammation and estrogen metabolism, both crucial in BC progression. It visualizes how specific microbial populations interact with immune cells, suppressing or enhancing immune responses in ways that could impact tumor aggressiveness and responsiveness to therapy. Additionally, the figure details how microbiota-driven epigenetic regulation of miRNAs influences immune cell function, shaping immune surveillance within the tumor. A section also illustrates how microbial metabolism contributes to bile acid synthesis, affecting inflammation and immune modulation in the TME. Annotations emphasize new research on the gut-tumor axis, linking microbiome composition to immune modulation. This figure thus underscores emerging therapeutic avenues focused on modifying the microbiome to reprogram cold tumors, potentially enhancing the efficacy of immunotherapies in BC.

### Gut microbiota in immune response and inflammation

Changes in gut bacteria can lead to a decrease in the differentiation of pathogenic T-cells, which are involved in inflammatory diseases, or an increase in the production of Tregs, which mediate immune tolerance. Studies have also identified an estrogen-independent link between BC patients’ gut microbiota and immune responses. Patients with IgA^+^ microbiota exhibit decreased fecal microbiota richness and α-diversity compared to those with IgA^−^ microbiota. These findings suggest that gut microbiota may significantly influence BC risk by altering immunological, metabolic, and estrogen-recycling processes [269]. The mucosal surface of the gut acts as a barrier, promoting symbiosis between host and microbes. When this barrier is breached, microbes can disrupt the immune system’s response to tumors, potentially creating immunosuppressive or proinflammatory microenvironments. This disruption can exacerbate BC by generating chronic inflammation, primarily by overexpressing toll-like receptors (TLRs) and the NF-κB pathway—a key inflammation player. This pathway triggers the production of various cytokines, such as IL-6, IL-18, IL-12, and IL-17, and activates TNF-α, which prolongs inflammation in surrounding tissues. Prolonged activation of TLRs can promote tumor cell proliferation, invasion, and metastasis by regulating cytokines, metalloproteinases, and proinflammatory integrins [269].

### Gut microbiota in epigenetics modifications of cold-to-hot BC transformation

The gut microbiome contributes significantly to BC progression, particularly in transforming child-to-hot BC transformation. Gut microbiota produces bioactive metabolites like butyrate and folate, which influence DNA methylation and histone modifications in tumor cells, activating or silencing genes involved in cancer development. The gut microbiota, which interacts with the tumor physiologically and ecologically, is a key factor in epigenetic dysregulation. These microbes produce low molecular weight bioactive compounds, such as folates, biotin, and short-chain fatty acids (butyrate and acetate) that participate in epigenetic processes. Additionally, gut microbiota influences the absorption and excretion of cofactors, such as iodine and zinc, which are crucial for enzymes involved in epigenetic modifications. While a direct causal link between bacterial epigenetic activation and breast tumor formation has not yet been established, gut bacterial influence can induce hypermethylation and epigenetic reprogramming in humans, contributing to tumor processes [269].

Microbiota dysbiosis can also alter biological processes, including protein and gene expression, and miRNA regulation, which are linked to cancer. The gut microbiota can influence miRNA through changes in gene expression or the MyD88-dependent pathway. In BC, gut microbiota can induce the overexpression or suppression of specific miRNAs, such as miR-21, miR-106a, miR-155, miR-126, miR-199a, and miR-335. These miRNAs are associated with tumor development and sex hormone expression [274].

### Microbiome and bile acids in cold-to-hot Bc transformation

Bile acids play a dual role in cancer, acting as both protective and carcinogenic agents. While bile acids can prevent BC, they can also be carcinogenic in gastrointestinal cancers. Bile acid metabolism has been linked to an elevated risk of BC, particularly concerning specific metabolites. A comprehensive study of three cohorts identified distinct microbial compositions. In groups with heightened bile acid metabolism, there was a higher abundance of *Gammaretrovirus*,* Hymenobacter*,* Anaerococcus*,* and Collimonas*. Conversely, groups with lower bile acid metabolism, which were characterized by enriched cell proliferation-associated genes, showed higher abundances of *Lactobacillus*,* Ruegeria*, and *Marichromatium*. This observation underscores the potential significance of microbial diversity in modulating bile acid metabolism and its impact on BC risk. Additionally, a reduction in specific microflora in BC patients is associated with decreased levels of secondary bile acids, such as lithocholic acid and cadaverine, in blood circulation [276].

In conclusion, the gut microbiota plays a pivotal role in regulating estrogen levels and influencing tumor transformation in BC. The gut microbiome’s impact on hormone metabolism, mainly through enzymes like β-glucuronidase, can elevate circulating estrogen levels, fostering a pro-inflammatory TME that promotes tumor growth and immune cell infiltration. Dysbiosis and increased β-glucuronidase activity in the gut can lead to higher estrogen reabsorption, which is linked to more aggressive BC phenotypes and enhanced metastatic potential. Research highlights the significant relationship between gut microbiota and immune responses in BC. For instance, IgA^+^ microbiota has been associated with decreased microbial diversity and altered immune responses, suggesting a complex interplay between the gut and systemic immunity. This influence extends to inflammatory pathways, notably through TLRs and the NF-κB pathway. These can exacerbate chronic inflammation and contribute to BC progression by regulating cytokines and other pro-inflammatory mediators.

Furthermore, the gut microbiome contributes to epigenetic modifications in BC, affecting DNA methylation and histone modifications through metabolites like butyrate and folate. These changes can activate or silence genes involved in cancer development, highlighting the microbiome’s role in tumor epigenetics. Dysbiosis can lead to alterations in miRNA expression, influencing tumor behavior and sex hormone regulation, which are crucial in BC pathogenesis. Bile acid metabolism, influenced by gut microbiota, also plays a dual role in cancer. Studies have shown that microbial diversity affects bile acid metabolism, with specific microbial populations linked to higher or lower BC risk.

Overall, the intricate relationship between gut microbiota and BC underscores the potential for microbiome-based therapeutic interventions. By targeting the gut-tumor axis, future therapies could aim to modulate the microbiome to transform cold tumors into hot ones, enhancing the efficacy of immunotherapies and improving outcomes for BC patients. This emerging field holds promise for developing novel strategies to combat BC through microbiome modulation, emphasizing the need for continued research and clinical exploration.

### Additional Immunomodulatory strategies for cold-to-hot tumor transition

#### Vaccine immunotherapy

Recent advancements in immunotherapy have highlighted the potential of combining vaccines and ICIs to enhance anti-tumor responses in BC, particularly in TNBC. One promising approach involves using a lipid/calcium/phosphate (LCP) nanoparticle-based mRNA vaccine expressing the complete MUC1 protein combined with an anti-CTLA-4 mAb to enhance immunity against TNBC. This combination therapy has shown considerable efficacy in remodeling the tumor TME by reducing tumor-infiltrating immune cells such as Tregs and MDSCs while decreasing cytokines like IL-6, TGF-β, and TNF-α. There was a notable increase in CD8^+^ T-cells, IL-12, and IFN-γ, enhancing the cytotoxic effect against tumor cells. The therapy also reduced levels of p-STAT3 and STAT3 in the TME, decreased the density of α-smooth muscle actin (α-SMA) and CD31 markers, and further increased CD8^+^ T-cell infiltration, collectively contributing to reduced tumor size, enhanced tumor cell apoptosis, and inhibited tumor growth [277]. Similarly, mRNA vaccines encoding MUC1-hemagglutinin (HA) Tag in LCP nanoparticles have demonstrated successful expression in 4T1 cells and lymph nodes, augmenting tumor-infiltrating CD8^+^ T-cells and reducing tumor size when combined with anti-CTLA-4 in mouse models. Notably, this combination therapy was associated with minimal toxicity, as evidenced by a slight reduction in white blood cell counts without significant effects on hemoglobin or kidney and liver function [278].

A new personalized immunotherapy strategy for metastatic TNBC has shown promising results. Using a protein transfer method, this approach incorporates GPI-B7-1 and GPI-IL-12 molecules onto tumor membrane vesicles (TMVs) derived from the patient’s tumor tissue. When tested in the 4T1 TNBC mouse model, the TMV vaccine, especially when combined with the anti-CTLA-4 antibody, significantly boosted serum levels of several cytokines and chemokines, including TNF-α, IFN-γ, IL-2, IL-4, IL-12, IL-18, IL-31, ENA-78, CXCL-1, MIP-2, MIP-1β, and LIF.

Another promising approach targets melanoma-associated antigen (MAGE), part of the cancer-testis antigen family, for BC immunotherapy. Researchers combined the demethylating drug 5-aza-2’-deoxycytidine (5DC) with CTLA-4 blockade to enhance the immune response against MAGE-A-expressing tumor cells. MCF-7 cells showed the highest MAGE-A expression among the tested BC cell lines, followed by MDA-MB-453 and MDA-MB-231 cells. Treatment with 5DC increased MAGE-A expression in a dose-dependent manner, and when combined with CTLs, it led to more effective tumor cell lysis. The combination of anti-CTLA-4 antibody and 5DC significantly elevated IFN-γ levels and improved the lytic ability of CTLs. These findings suggest that these innovative strategies could enhance immune responses and improve outcomes for patients with metastatic TNBC and other BCs expressing MAGE-A [279].

HER2-targeting therapies have also been explored, with trastuzumab deruxtecan (DS-8201a), an antibody-drug conjugate, showing potent antitumor activity. Combined with an anti-CTLA-4 antibody in a mouse model, this therapy demonstrated superior antitumor effects compared to monotherapy, significantly increasing CD45^+^, CD4^+^ T, and CD8^+^ T-cell numbers and extending OS. Notably, a CR rate of 85% was achieved, and experiments confirmed immune memory formation through this combination therapy [280]. Additionally, the efficacy of other monoclonal antibodies targeting CTLA-4 and PD-L1, identified as ID-1 and PD-L1_1, respectively, has been evaluated compared to FDA-approved ipilimumab and atezolizumab. The novel mAbs demonstrated superior activation of NK and Pan T-cells, with higher cytokine release and cell lysis than their FDA-approved counterparts, suggesting their potential for more effective cancer immunotherapy [281]. A recent study explored metronomic chemotherapy regimens combined with anti-CTLA-4 therapy in various BC models. Combining low-dose cyclophosphamide (CTX) with anti-CTLA-4 resulted in significant tumor growth inhibition, although sequential treatment with gemcitabine yielded the most significant antitumor effect. These findings suggest that combining CTLA-4 blockade with chemotherapeutic agents like gemcitabine, either sequentially or concomitantly, may offer improved therapeutic outcomes [282].

To sum up, recent advancements in immunotherapy show promise for treating BC, particularly TNBC. A new strategy involves using an LCP nanoparticle-based mRNA vaccine that expresses the MUC1 protein. Combined with an anti-CTLA-4 antibody, this therapy remodels the TME, reduces Tregs and MDSCs, and increases CD8^+^ T-cells and cytokines like IL-12 and IFN-γ, enhancing tumor cytotoxicity. Another approach uses TMVs with GPI-B7-1 and GPI-IL-12 from patient tumor tissue. In a TNBC mouse model, this TMV vaccine and anti-CTLA-4 significantly boosted cytokines and chemokines, leading to 70% survival beyond 66 days with reduced lung metastasis. Combining the demethylating drug 5DC with CTLA-4 blockade also targets MAGE in BC, enhancing CTL activity and increasing IFN-γ levels. These strategies highlight the potential of combining vaccines and ICIs to improve immune responses and outcomes in metastatic TNBC.

Moreover, the success of cancer vaccines hinges on eliciting strong T-cell responses, supported by diverse neoantigen vaccine systems—such as virus-like particles, engineered bacteria, and nanomaterials—that enhance antigen presentation. Adjuvants, including IFN-γ, IL-2 variants, and TLR agonists, further activate APCs and T cells while countering immune suppression (e.g., Tregs, MDSCs, TIM-3). Immunogenicity also depends on the vaccination route: subcutaneous administration elicits vigorous T-cell activity, whereas intravenous delivery increases memory-precursor cells and strengthens synergy with checkpoint inhibitors. Complementary approaches involve manipulating epigenetics to boost neoantigen expression, harnessing the gut microbiome for immune cell maturation, and regulating inflammation to maximize vaccine-based immunotherapy [283].

### rAd.sT oncolytic adenovirus

rAd.sT is an engineered oncolytic adenovirus that inhibits aberrant TGFβ signaling in cancer. In a 4T1 BC model, rAd.sT significantly reduced tumor weight, inhibited lung metastasis, and downregulated angiogenesis- and metastasis-related genes such as VEGFA, CXCR-4, and E-cadherin. The treatment also shifted the immune response from Th2 to Th1 by decreasing TGFβ-1, IL-6, and IL-10 levels while increasing TNF-α, IL-2, IL-12, and IFN-γ. This led to enhanced CD8^+^ T-cell activation, reduced MDSCs and Tregs, and increased memory T-cells. When combined with anti-PD-1 and anti-CTLA-4 therapies, rAd.sT further reduced tumor volume suppressed metastatic nodules, and showed potential for reprogramming the TME [284].

Building on the success of rAd.sT, researchers developed rAd.GM, an oncolytic virus carrying the GM-CSF gene, enhances immune activation in TNBC. The combination therapy also enhanced CD8^+^ T-cells, reduced CD4^+^ T-cells, decreased Treg infiltration, and reprogrammed TAMs. Additionally, it upregulated Th1 cytokines and cytotoxicity-related genes while downregulating Th2 cytokines, highlighting the potential of rAd.GM enhances immune responses and inhibits tumor progression more effectively [285].

### Cancer stem-like cells

It has been demonstrated that cancer stem cells (CSCs) are key drivers of tumorigenesis and relapse. A growing body of evidence reveals that the tremendous capacity of CSCs to resist innate and adaptive anti-tumor immune responses is directly mediated by their immunomodulatory properties. The TIME is controlled by CSCs, which can hijack the anti-tumor functions of immune cells, thereby providing self-protection from immune attack while enhancing the infiltration and activity of pro-tumor immune cells. To date, cancer immunotherapy strategies have primarily been developed without accounting for the immunosuppressive properties of CSCs, resulting in altered clinical efficacy and perpetuating tumor progression and relapse. Therefore, it is considered essential that the signals underlying CSC immune evasion be targeted to improve the effectiveness of immunotherapy and reduce tumor relapse [286].

It has been observed that CSCs suppress CD8⁺ T-cell infiltration while promoting the recruitment of M2 macrophages and the activity of N2 neutrophils. A positive association has been reported between CSC expansion and high PD-L1 expression in the TME, with PD-L1 levels found to be higher in CSCs than in cancer cells. PD-L1 expression in metastatic cancer cells induces a dedifferentiation program by stimulating an epithelial-mesenchymal transition (EMT) profile, replenishing the CSC population within the tumor. Targeting CSCs is thus considered essential for effective tumor eradication and the reduction of tumor recurrence following immunotherapy. Numerous signaling pathways have been implicated in the enrichment of CSCs within the tumor, with TGFβ particularly interesting. TGFβ is known to induce a dedifferentiation program, and its function as a bridge between EMT and increased PD-L1 levels provides a rationale for the combined use of TGFβ and anti-PD-L1 inhibitors to reinvigorate immune activity in patients undergoing ICI therapy [287].

CSCs play a critical role in TNBC, contributing to therapeutic resistance, metastasis, and recurrence. The CHI3L1/MAF/CTLA4 signaling pathway in triple-negative BCSCs (TN-BCSCs) was studied using single-cell transcriptomics, revealing 14 cell clusters, including B cells, T cells, and stem cells. CHI3L1 was highly expressed in TN-BCSCs and was shown to enhance TNBC stemness by increasing CD44^+^/CD24^−^ cells and promoting proliferation, migration, and resistance to apoptosis. Co-culture experiments indicated that TN-BCSCs upregulate CTLA4 expression in T-cells, leading to immune escape via the CHI3L1/MAF pathway. An in situ transplantation model showed that inhibiting CHI3L1 reduced tumor growth and downregulated MAF and CTLA4, while BCSCs increased S100A4 levels, further supporting the role of TN-BCSCs in immune escape [288].

In another study, eosinophils were identified as a potential target to improve immune checkpoint blockade therapy in BC. Anti-CTLA4 therapy increased CD8a^+^ and CD4^+^ T-cell infiltration in EO771 and MMTV-PyVT models but not in MCaP0008. Tumor-associated eosinophils (TAEs) showed an 85% sensitivity to anti-CTLA4 therapy in EO771 tumors. TAEs depletion reversed the antitumor effects of anti-CTLA4 treatment, reducing the expression of genes related to antitumor immunity and angiostasis, although it did not affect T lymphocyte infiltration. Additionally, anti-CTLA4 therapy upregulated CCL5, IL5, and CCL11, which are chemoattractants for T lymphocytes and eosinophils [289]. Furthermore, PBMCs from 20 healthy women and 20 BC patients were analyzed for Treg cell markers following phytohemagglutinin (PHA) activation. Gene expression levels of FoxP3, CTLA4, and GITR were significantly higher in both unstimulated and PHA-stimulated PBMCs from BC patients compared to healthy controls, indicating heightened Treg cell activity that may contribute to tumor progression by suppressing anti-tumor immune responses [290]. RON, also known as macrophage stimulating-1 receptor, is an RTK often overexpressed in breast tumors and linked to metastasis and poor patient outcomes. This study explored the effects of treating macrophages from wild-type (RON^WT^) and RON knockout (RON^−/−^) mice with recombinant macrophage-stimulating protein (MSP). In WT macrophages, MSP treatment increased the expression of CD80 and PD-L1. However, this upregulation was blocked when treated with RON inhibitors, BMS777607 and merestinib (LY2801653), through the MAPK and PI3K pathways. Combining RON inhibitors with anti-CTLA-4 therapy showed a 92% clinical benefit in PyMT-NP tumor models, including significant tumor shrinkage [291]. In contrast, combining RON inhibitors with anti-PD-1 was less effective. In RON^−/−^ mice, the combination of RON inhibition and anti-CTLA-4 did not reduce tumor growth more than anti-CTLA-4 alone. However, it did enhance anti-tumor T-cell responses, increasing the presence of PD-1^+^ CD62L^low^ CD4^+^ and CD8^+^ T-cells and the production of PRF1, TNF-α, and IFN-γ. The combination of RON inhibitors and anti-CTLA-4 was particularly effective for lung metastasis, reducing the metastasis area and increasing CD8^+^ T-cell infiltration. Notably, 42% of macro metastases were cleared entirely, and there was a significant reduction in the area occupied by metastases in RON TK^−/−^ mice [291].

### Future directions

#### Potential side effects

One of the major barriers to the widespread adoption of immunotherapies is the risk of irAEs. These events arise as collateral damage when the immune system is activated to combat tumor cells, leading to autoimmune toxicities that may affect any organ system. Common manifestations include colitis, pneumonitis, hepatitis, dermatitis, and endocrinopathies [292, 293]. The onset, severity, and resolution of irAEs are highly variable, necessitating individualized management approaches. Preclinical and clinical studies have shown that the modulation of innate immune pathways, such as the activation of type I interferon signaling or the STING pathway, can reprogram the breast TME [294, 295]. While these strategies hold promise for achieving robust antitumor responses, they may simultaneously exacerbate systemic immune activation, thereby increasing the likelihood of irAEs [293].

Furthermore, approaches combining ICIs with agents targeting tumor-intrinsic factors—such as HER2-directed therapies—present unique challenges in balancing efficacy and safety. To address these complexities, dynamic biomarker monitoring is critical to predict the risk of irAEs while evaluating the extent of immune activation [296]. When coupled with personalized therapeutic regimens, such strategies may optimize the benefits of cold-to-hot translation in BC while mitigating the associated toxicities [293]. A recent systematic review and meta-analysis of nine studies involving 4687 participants demonstrated that ICIs in BC treatment were associated with higher frequencies of any grade and grade 3–5 AEs and irAEs than conventional therapies. Notable irAEs included hyperthyroidism, hypothyroidism, and adrenal insufficiency, while non-immune AEs such as increased aspartate aminotransferase and cough also showed significant risk elevations with ICIs [297].

Current management strategies are primarily centered on glucocorticoid administration, often effective but insufficient for steroid-refractory cases such as colitis, myocarditis, and pneumonitis, which carry high mortality rates [298]. Alternatives such as infliximab and IVIG are being explored through clinical trials, including investigations of vedolizumab for steroid-refractory colitis (NCT04407247) [299] and infliximab/IVIG combinations for pneumonitis (NCT04438382) [300]. These studies aim to establish evidence-based protocols for managing severe irAEs. Recent advances in the field emphasize the role of predictive biomarkers in stratifying irAE risk. Cytokine profiles, HLA genotypes, and gut microbiome composition have emerged as promising avenues. For instance, specific HLA alleles have been implicated in irAE susceptibility, with trials such as NCT04107311 focusing on autoimmune panels and intestinal microbiomes as predictors of irAEs requiring immunosuppression [301].

Furthermore, gut microbiota modulation—such as using Bifidobacterium or fecal microbiota transplantation—has shown potential for mitigating and preventing irAEs without compromising therapeutic efficacy. Emerging preclinical models also provide deeper mechanistic insights. For example, a genetic mouse model mimicking ICI-induced myocarditis has highlighted the roles of CTLA-4 and PD-1 interactions and suggested abatacept as a therapeutic candidate [293]. These models may bridge critical gaps in understanding irAE biology, guiding therapeutic innovation.

Future research must aim to refine predictive biomarkers for irAEs and develop more selective checkpoint inhibitors and precision-engineered CAR-T cells with tunable activation thresholds to minimize off-target effects. Unlike highly immunogenic cancers such as melanoma or lung cancer, BC is often considered an immunologically cold tumor, characterized by lower levels of TILs and reduced neoantigen load. This creates a distinct hurdle, as strategies to enhance immune activation, such as ICIs, may face reduced efficacy and exacerbate irAEs due to the need for more muscular immune stimulation to convert cold tumors into hot ones [302]. The future of immunotherapy in BC thus requires a careful balancing act—developing combination therapies or biomarkers that selectively activate anti-tumor immunity without triggering excessive autoimmune toxicity. On the other hand, advances in single-cell sequencing, immune phenotyping, and systems biology are poised to enhance further the ability to profile and mitigate irAE risks at the individual BC patient level [298]. The integration of multidisciplinary immune-related toxicity teams has been transformative in improving irAE diagnosis, management, and research. These teams enable the timely recognition of irAEs and support the development of sophisticated care models. As our understanding evolves, the interplay between irAEs and therapeutic outcomes will likely uncover opportunities to optimize the efficacy and safety of immunotherapies.

### Immunosculpting and immune resistance

Immunosculpting describes how tumor-specific and TME-mediated resistance mechanisms create spatial transcriptomic barriers that define immune landscapes within tumor regions, thereby revealing distinct resistance niches [11, 303]. In cold tumors, areas of immune exclusion have been identified, and these regions are being targeted through the localized delivery of immunostimulatory agents using nanoparticle-based carriers. Such systems can co-deliver ICIs, cytokines, or tumor antigens to stimulate immuneevasive regions selectively. Although these approaches have been extensively studied in TNBC—where the TME is often heterogeneous—it is expected that similar strategies may be applied to other BC subtypes to shift the balance toward effective immune activation [22, 304].

The TME in BC has been recognized as a key determinant of resistance to immune checkpoint therapies. It is increasingly appreciated that resistance is mediated by both tumorintrinsic factors and tumorextrinsic influences that shape the TME. While much of the research has focused on TNBC, recent investigations have highlighted the unique resistance mechanisms in luminal A, B, and HER2^+^ subtypes. These subtypes are often characterized by cold tumors, in which immune cell infiltration is low and immunosuppressive factors dominate, thereby limiting the efficacy of immune-based treatments.

### Mechanistic insights

It has been demonstrated that tumorintrinsic mechanisms—such as genetic and epigenetic alterations—contribute to resistance by reducing antigenicity and disrupting antigen processing and presentation. In luminal breast tumors, a low tumor mutation burden and reduced neoantigen formation have been observed [305]. At the same time, estrogen receptor (ER) signaling has been implicated in downregulating MHCI molecules [306]. In addition, alternative inhibitory pathways, such as the upregulation of B7H4, have been reported despite low PDL1 expression [307]. In HER2^+^ breast tumors, despite a higher degree of inherent immunogenicity, resistance further depends on HER2-driven signaling that activates pro-tumor pathways, such as PI3K/AKT, and by adaptive mechanisms, including HER2 downregulation or shedding [308]. The resulting upregulation of PDL1 in response to IFNγ released by activated T cells has been documented, contributing to immune escape [309].

Tumorextrinsic resistance is primarily imposed by the TME, which is populated by immunosuppressive cell types, including Tregs, MDSCs, and TAMs. These cells are enriched by hormonal influences in luminal BC and oncogenic signals in HER2^+^ tumors. Tregs and MDSCs are recruited to the TME by soluble factors such as IL-10 and TGFβ, which dampen CTL responses [310]. TAMs, mainly, are highly abundant in TNBC, often comprising over 50% of the TME, and are predominantly polarized toward an M2-like phenotype. M2TAMs are characterized by the expression of CD68 and CD163 and are known to secrete immunosuppressive cytokines (e.g., IL-10, CCL17, CCL22) that promote angiogenesis, matrix remodeling, and tumor cell migration. It has been reported that anti-PDL1 antibodies may partially reverse M2 polarization, yet TNBC cells have been shown to strongly induce M2 differentiation, thereby enhancing resistance to both immunotherapy and chemotherapy [311].

At the tumor-specific resistance level, genomic aberrations affecting tumorsuppressor genes such as ATM, PTEN, P53, LKB1, and CHEK2 have been reported, allowing tumor cells to bypass cellcycle checkpoints and proliferate uncontrollably. In TNBC, driver mutations in DNA repair genes, including NBS1, BRCA1, and BRCA2, confer additional tumorigenic advantages and may facilitate immune evasion by disrupting antigen presentation. Tumors have been found to downregulate immunogenic antigens and to interfere with antigen-processing pathways by reducing the expression of proteins such as TAP1, TAP2, and TAPBP [312]. Moreover, loss of heterozygosity and epigenetic silencing of MHCI molecules have been associated with poor clinical outcomes. These disruptions have been proposed as potential targets for DNA methyltransferase inhibitors to restore immune recognition [313].

At the TME level, tumors’ ability to block effective immune responses has been attributed to reduced chemokine production and the establishment of a local immunosuppressive milieu. Tumors have been shown to limit TILs recruitment and to promote the formation of an environment that favors tumor progression and metastasis [314]. In TNBC, the balance between immunosuppressive cells and activated effector Tcells is critical for therapeutic response. Mature dendritic cells and macrophages enhance anti-tumor immunity; immature or M2-polarized cells contribute to immune evasion. Tumors exploit this process by inducing macrophage polarization into TAMs and skewing NK cells toward an immunosuppressive phenotype when checkpoint molecules such as PDL1 are expressed. Neutrophils also play a paradoxical role; although they initiate inflammation and phagocytosis, NETs have been implicated in promoting tumor cell adhesion and metastasis, with an accumulation of TANs correlating with poor prognosis [315].

### Therapeutic strategies

Therapeutic strategies to overcome resistance to immunotherapy in BC subtypes other than TNBC are being actively developed. In luminal BC, combination therapies have been investigated in which ICIs are administered alongside neoadjuvant chemotherapy. It has been observed that chemotherapy can induce ICD, releasing tumor antigens and danger signals that promote dendritic cell maturation and T-cell activation. This process is believed to convert the TME from cold to “hot,” resulting in enhanced Tcell infiltration and improved clinical responses.

Traditional cytotoxic agents and targeted therapies (like bevacizumab and sunitinib) have limited efficacy. ICIs, though promising due to TNBC’s high mutational burden and immune cell infiltration, yield only modest responses when used alone. Resistance arises from complex interactions within the TME, including immune cell-mediated signaling pathways that drive both primary and acquired resistance. Ongoing research seeks to identify biomarkers and optimize combination therapies to overcome these resistance mechanisms [74]. Anti-PD-1 therapy has marked a breakthrough in TNBC treatment, yet only a few patients achieve durable remissions. To maximize the benefits of ICIs in TNBC, There are three strategies: refining patient selection through multifaceted biomarkers to predict response and resistance better; combining ICI with targeted therapies or chemotherapies—such as inhibitors of the PI3K/AKT or RAS/MAPK/ERK pathways—to enhance synergistic effects; and exploring alternative immunotherapy strategies beyond the PD-1 axis [316]. Furthermore, TNBC exhibits multiple mechanisms of resistance to therapy.

Endocrine therapies have been shown to influence the TME for luminal BC by upregulating MHCI expression on tumor cells, potentially enhancing immune recognition. Trials combining ICIs with endocrine therapy have suggested that such combinations may extend response durations in patients with endocrine-resistant disease [317]. In HER2^+^ BC, the integration of HER2-targeted treatments (e.g., trastuzumab) with ICIs has been evaluated, with evidence indicating that the combination may improve outcomes in patients with PDL1^+^ tumors. However, patient selection based on immune markers and molecular profiling remains critical, as resistance mechanisms such as the shedding of HER2 and upregulation of alternative checkpoints may undermine therapeutic efficacy [318].

Beyond conventional checkpoint blockade, novel approaches have been explored. Emerging agents targeting alternative checkpoints—such as LAG-3, TIGIT, and B7H4—have shown potential in reinvigorating exhausted Tcells. Therapeutic modulation of the TME is also being pursued by employing agents designed to deplete immunosuppressive myeloid cells, such as CSF-1R inhibitors and oncolytic viruses that provoke localized inflammatory responses [319]. Epigenetic therapies have been used to upregulate antigen presentation machinery. In contrast, adoptive cell therapies, including TIL and CART cell therapies, are being refined to enhance their efficacy in the context of a suppressive TME [320].

In addition, localized immunosculpting approaches have been proposed to target immuneevasive niches, specifically within cold tumors. The TME is anticipated to be reshaped into a more hot immuneactive state by using nanoparticle-based carriers to deliver immunostimulatory agents directly into these regions. This multifaceted strategy aims to convert immune-resistant tumors into ones more responsive to immunotherapy, thereby improving clinical outcomes [321].

Considering the potential for immunosculpting and immune resistance to affect immunotherapy responses in BC, the future success of ICI therapy in cold or immune-cold BC depends on two critical factors: identifying reliable biomarkers and optimizing patient selection. Currently, eligibility for ICI therapy is primarily based on PD-L1 expression. However, many PD-L1^+^ tumors do not respond well to ICI, highlighting the urgent need for additional biomarkers to predict which patients will benefit from these therapies more accurately.

BC patients, particularly those with metastatic TNBC, often undergo chemotherapy or radiation, which can significantly alter the TME [135]. These therapies can either promote immunosuppressive populations or enrich immune-inflammatory populations, impacting the success of subsequent immunotherapy [322]. Chemotherapy may induce ICD and T-cell infiltration but also lead to chemotherapy-induced immune editing, which could limit ICI efficacy [323]. Understanding these changes is vital for determining the most effective immunotherapeutic combinations while balancing risks of toxicity and cost. Clinical trials indicate that ICI therapy has shown promise in some settings. However, the molecular mechanisms underlying adaptive resistance, such as the upregulation of other immune checkpoints (LAG-3, TIM-3, TIGIT), need further exploration [135]. Tumors may also downregulate antigen presentation machinery in response to ICI, contributing to immune evasion [324]. These findings emphasize the importance of molecular profiling pre- and post-treatment to understand resistance mechanisms better. For the future, a more nuanced approach to ICI therapy in cold BC is needed, incorporating better patient stratification, deeper molecular analyses, and potentially earlier or combination-based strategies. This will maximize ICI efficacy while minimizing adverse effects, offering a more personalized and precise treatment for BC patients.

### Combining therapies and long-term immune surveillance

Clinically, the evolution of cancer immunotherapy emphasizes integrating diverse therapeutic approaches to overcome tumor resistance and improve patient outcomes synergistically. Recent insights underscore this shift, as their meta-analysis of randomized clinical trials demonstrated significant improvements in OS and PFS with combination immunotherapies compared to monotherapies across Phase I-IV trials. These combinations, encompassing chemotherapy, radiation, targeted inhibitors, hormonal therapy, and endocrine therapy, provide a multi-pronged strategy for addressing BC, significantly advancing the management of resistant tumors and improving patient survival outcomes [325].

Emerging strategies explore the combination of anti-PD-1 and anti-PD-L1 agents with CAR-T cells targeting TAAs [326, 327]. A Phase I study evaluated CRISPR-Cas9-engineered mesothelin-specific CAR-T (MPTK-CAR-T) cells with PD-1 and TCR deficiencies in a dose-escalation trial involving 15 patients with solid tumors. The treatment showed no dose-limiting toxicity or unexpected AEs, with stable disease observed in 2 out of 15 patients. MPTK-CAR-T cells peaked in circulation between days 7–14 but became undetectable after one month. TCR^+^ CAR-T cells were predominantly detected in patient samples post-infusion, highlighting the reduced persistence of TCR-deficient CAR-T cells [328]. Animal models further confirmed the critical role of the natural TCR in CAR-T cell persistence. While the approach is feasible and safe, further optimization is needed to enhance its efficacy in solid tumors [327].

Furthermore, the integration of microbiome-based therapies into combinatorial regimens has garnered attention [329]. Studies reveal that modulating the gut microbiota can reshape systemic immunity, converting immunologically cold tumors, which evade immune recognition, into hot tumors capable of eliciting robust immune responses. Such approaches enhance the efficacy of existing therapies and expand their applicability to previously unresponsive cancer types. Recent studies have highlighted the significant role of the microbiota in modulating the effectiveness and side effects of cancer immunotherapies. Research into the relationship between gut microbiota composition and the clinical outcomes of immunotherapy has revealed distinct microbial signatures associated with favorable or unfavorable responses to the treatment of melanoma and lung cancer [330–332].

In the recent narrative meta-analysis, the role of gut microbiota in influencing the outcomes of BC treatment with ICIs is explored through 13 relevant studies, including both clinical and pre-clinical research [333]. The findings suggest that the diversity and composition of gut microbiota are linked to patient responses to immunotherapy. Clinical studies showed that specific microbiota profiles may predict treatment success. In contrast, pre-clinical studies emphasized the impact of dysbiosis—imbalanced gut microbiota—caused by factors such as obesity, antibiotics, and diet, which can impair immune responses and affect the efficacy of PD-L1 inhibitors. Furthermore, microbiota-modulating treatments, like probiotics, have been shown to potentially enhance immunotherapy responses, suggesting their role as adjunct therapies in BC management. A post-hoc analysis showed microbiome samples from 44 patients with ER^+^ HER2^−^ BC and assessed the effects of pembrolizumab in combination with eribulin. Microbiome samples were sourced from fecal samples and analyzed using 16 S rRNA sequencing and metagenomic shotgun sequencing. The study evaluated the safety and efficacy of chemotherapy combined with an ICI, while also investigating drug-related microbial toxicity and microbiome composition [334].

Additionally, a retrospective analysis within the AMTEC trial (NCT03801369) found a significant correlation between gut microbiome diversity and tumor biomarkers, including PD-L1 expression, tumor immune cell density, TILs, mutation signatures, and interferon gene signatures, among 10 patients with TNBC receiving olaparib and durvalumab. This study aimed to understand clinical responses to therapy better. Similarly, a post-hoc analysis of the I-SPY2 trial (NCT01042379) involving 66 patients with TNBC and ER^+^, HER2^−^ subtypes, assessed the impact of antibiotic use during treatment with pembrolizumab, paclitaxel, doxorubicin, and cyclophosphamide. Although microbiome samples were analyzed, the specific analysis method was not detailed. The primary outcomes included residual cancer burden and pathologic CR rates [335]. These studies collectively highlight that gut microbiota composition may serve as a predictive biomarker for immunotherapy responses in BC and suggest that modulation of the microbiota could enhance the effectiveness of chemo-immunotherapy. However, the evidence remains limited, and more extensive prospective studies are required to fully understand the microbiota’s role as both a biomarker and a therapeutic target in BC treatment. Importantly, these studies emphasize the need for a nuanced understanding of the interplay between microbiota and cancer therapies. While a diverse and balanced microbiota enhances clinical responses, certain microbial species have been identified as beneficial and detrimental, underscoring the complexity of this relationship. Integrating microbiota profiling into cancer treatment strategies could pave the way for personalized approaches that optimize immunotherapy efficacy and minimize adverse effects.

Innovative therapeutic modalities such as bispecific T-cell engagers (BiTEs) and antibody-drug conjugates (ADCs) offer additional dimensions in cancer treatment. BiTEs link T-cells directly to tumor cells, facilitating precise immune attacks, while ADCs deliver cytotoxic drugs specifically to cancer cells, minimizing off-target effects and enhancing treatment specificity [336]. A recent study highlights that the conjugated αPD-1-(iRGD)2, a PD-1 antibody-iRGD cyclic peptide conjugate developed through glycoengineering, enhances tissue penetration while simultaneously targeting tumor cells and PD-1^+^ T-cells. This dual targeting promotes tumor-specific T-cell activation with minimal effects on non-specific T-cells. In syngeneic mouse models, αPD-1-(iRGD)2 effectively reduces tumor growth with favorable biosafety. Additionally, flow cytometry and single-cell RNA-seq reveal that αPD-1-(iRGD)2 remodels the TME and expands “better effector” CD8^+^ T-cells with a hot tumor phenotype, expressing stem- and memory-associated genes, suggesting its potential as promising novel cancer immunotherapy [337]. These strategies provide avenues for personalized and precision oncology, leveraging the strengths of immune targeting and pharmacological innovation.

While essential for sustained tumor suppression, persistent immune activation carries the risk of chronic inflammation, which can paradoxically create a microenvironment conducive to tumorigenesis. Addressing this requires a delicate balance: therapies must promote durable immune memory to provide long-lasting protection without continuous stimulation. Cutting-edge research focuses on enhancing immune memory by incorporating cytokine signaling modulators, long-lived memory T-cell generation, and novel vaccine platforms designed to sustain immune activity against residual cancer cells without eliciting excessive inflammation [338–340]. By refining these combinatorial approaches and addressing the complexities of immune surveillance, the future of cancer immunotherapy promises to achieve more durable and practical outcomes while ensuring patient safety and quality of life.

## Conclusion

Cold tumors, characterized by low TILs and reduced neoantigen load, require strategies to enhance immune activation. This involves the use of combination therapies or biomarkers that selectively activate anti-tumor immunity without triggering excessive autoimmune toxicity. The efficacy of ICIs and other immunotherapies can be significantly improved by cold-to-hot transition, with a robust immune presence. Considering the potential for immunosculpting and immune resistance to affect immunotherapy responses, the future success of ICI therapy in cold BC depends on identifying reliable biomarkers and optimizing patient selection. Current eligibility criteria based on PD-L1 expression are insufficient, and additional biomarkers are needed to predict therapeutic responses more accurately. The impact of chemotherapy and radiation on the TME also must be considered, as these treatments can alter immune populations and affect therapy outcomes. Overall, the future of BC immunotherapy requires a nuanced approach that balances immune activation to convert cold tumors into hot ones without triggering excessive autoimmune toxicity. Continued research and clinical trials will be essential in establishing evidence-based protocols and advancing the field toward more effective and safer treatments for BC patients.

## Electronic supplementary material

Below is the link to the electronic supplementary material.

Supplementary Material 1

## Data Availability

No datasets were generated or analysed during the current study.
