# Supplementary figures and images for: Reprogramming the breast tumor immune microenvironment: cold-to-hot transition for enhanced immunotherapy
Source: J Exp Clin Cancer Res. 2025 Apr 25;44:131. doi: 10.1186/s13046-025-03394-8 (PMC12032666; doi:10.1186/s13046-025-03394-8)

A

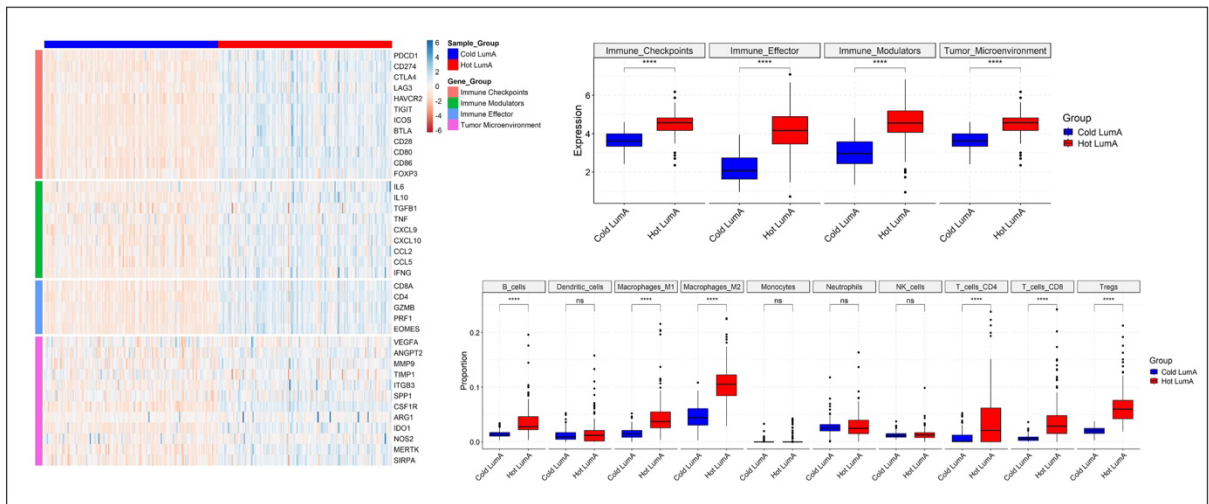

B

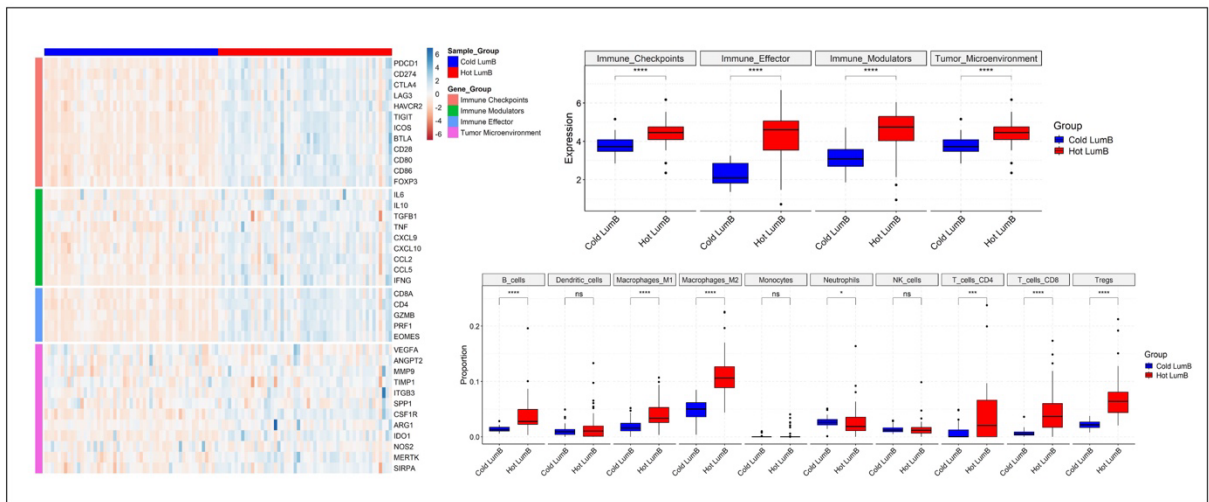

Figure S1.

Supplement: Supplementary file 1 — Supplementary Material 1 [file 13046_2025_3394_MOESM1_ESM.pdf]
